# A cyclic bis[2]catenane metallacage

**DOI:** 10.1038/s41467-020-16556-3

**Published:** 2020-06-01

**Authors:** Yiliang Wang, Yicheng Zhang, Zhixuan Zhou, Ryan T. Vanderlinden, Bin Li, Bo Song, Xiaopeng Li, Lei Cui, Jian Li, Xueshun Jia, Jianhui Fang, Chunju Li, Peter J. Stang

**Affiliations:** 1 School of Materials Science and Engineering, Center for Supramolecular Chemistry and Catalysis and Department of Chemistry, Shanghai University, Shanghai, 200444 China; 2 Department of Chemistry, University of Utah, Salt Lake City, UT 84112 USA; 3 Department of Chemistry, University of South Florida, Tampa, FL 33620 USA; 4 Key Laboratory of Inorganic-Organic Hybrid Functional Material Chemistry, Ministry of Education, Tianjin Key Laboratory of Structure and Performance for Functional Molecules, College of Chemistry, Tianjin Normal University, Tianjin, 300387 China

**Keywords:** Coordination chemistry, Supramolecular chemistry

## Abstract

Catenated cages represent chemistry’s challenging synthetic targets because a three-dimensional assembly is necessary for their formation. Herein, a cyclic bis[2]catenane is constructed through the coordination-driven self-assembly of the interlocked bis-metallacage, by the 90° Pt(II) heteroligation of the endo-functionalized double-bridged tweezer bearing pyridyl moieties and the tetra-carboxylated linker. NMR spectrometry, X-ray crystallography and mass spectrometry confirm the formation of a cyclic bis[2]catenane with “∞”-shaped topology via a 14-component self-assembly. Particularly, reversibly responsive transformation between the bis[2]catenane and the bis-metallacage can be realized by guest exchange, concentration effect and solvent effect. This work represents a novel example of a cyclic cage-based [2]catenane oligomer.

## Abstract (web summary)

Catenated cages are challenging synthetic targets in chemistry. Here, the authors employ a multi-component coordination strategy using a Pt(II) heteroligation to construct a cyclic bis[2]catenane metallacage, which could be reversibly transformed between the catenated structure and the bis-metallacage.

## Introduction

In the past decades, chemists have shown considerable interest in interlocked supramolecular architectures^1–3^, because these topologies are widely found in DNA^4^ and proteins^5^ and have been applied in molecular machines and other molecular devices^6^. In particular, catenanes^7–9^, which consist of two or more interlocked macrocycles or cages, are considered as one of the important type of assembled structures. Although their preparation was a synthetic challenge in early stages, the utilization of metal ion templates^10,11^ and reversible covalent bonds^12,13^ has resulted in high-yield and facile syntheses. Moreover, coordination-driven self-assembly has also been proven as an effective strategy for the synthesis of supramolecular coordination complexes (SCCs)^14,15^ with catenated structures^16–20^ with high synthetic yield.

Most catenanes are formed by macrocyclic molecules; interlocked assemblies of three-dimensional cage species are more synthetically challenging and comparatively rare. Since Fujita et al. first reported the catenated coordination cages in 1999^21^, several examples of the interpenetrated cages have been reported^12,13,22–27^. Although existing cyclic [2]catenane oligomers are known via bis-macrocycles^28–31^, there is no facile strategy for the formation of cage-based [2]catenane oligomers. Moreover, only a few reports provide insight into the reversible transformation between cage monomers and catenated structures^32^, which limit the application of these structures in the stimuli-responsive systems for sensing, information displays and molecular machines^33^.

Here, we employ the multi-component self-assembly strategy by using the Pt(II) heteroligation^34^ of the endo-functionalized double-bridged tweezer **3** and the tetra-carboxylated ligand to form the bis-metallacage. The resulting structure of interpenetrated cyclic bis[2]catenane metallacage with “∞”-shaped topology is characterized by NMR spectrometry, single crystal X-ray diffraction analysis and mass spectrometry. Due to the guest exchange, concentration effect, and solvent effect, the SCCs could be reversibly transformed between the bis-metallacage and the cyclic catenated structure.

## Results

### Synthesis of double-bridged tweezer

**Fig. 1:**
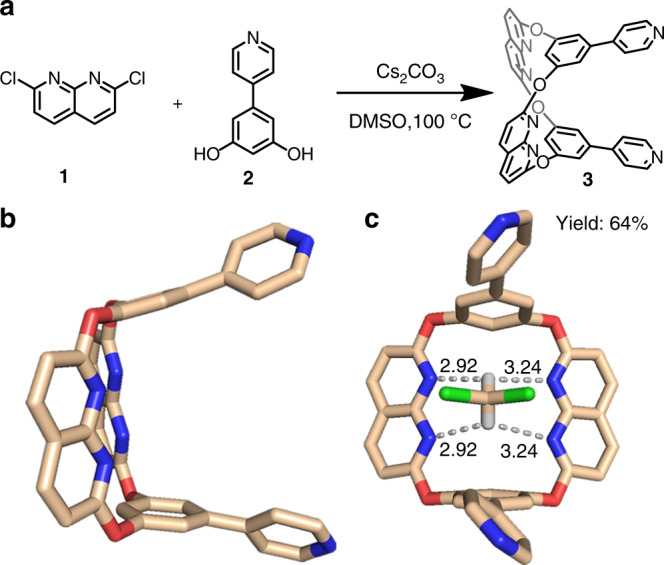
Synthesis and single-crystal structures of ligand 3. **a** Synthesis of endo-functionalized molecular tweezer **3**. **b** Side view of the solid-state structure of **3**: (hydrogen atoms are omitted for clarity). **c** Multiple C–H···N hydrogen bonds between the H atoms of DCM and the N atoms of the naphthyridyl groups: C–H···N distances (Å).

Ligand **3** with naphthyridyl spacers and pyridyl moieties was synthesized by the cyclocondensation of 2,6-dichloro-1,8-naphthyridine **1** and 5-(4-pyridinyl)-1,3-benzenediol **2** in the presence of Cs_2_CO_3_ at 100 °C (yield: 64%, Fig. 1a). **3** can be viewed as an oxacalixarene, and should exhibit a tweezer-like structure^35–37^. The naphthyridyl spacers can separate two aromatic walls at a distance of ~7 Å, permitting a suitable rigid cavity for π···π stacking. And the N atoms from naphthyridyl groups could be oriented in the endo-cavity and provide binding sites for hydrogen bonds. Moreover, two pyridyl moieties make **3** suitable for coordination-driven self-assembly with transition-metal acceptors. Single crystals of **3** were grown by the slow evaporation of **3** in a dichloromethane (DCM) and *n*-hexane (v/v = 1:1) solution, and analyzed by single-crystal X-ray diffraction. As expected, **3** adopts a 1,3-alternate conformation in solid-state, which can be assigned to a double-bridged tweezer (Fig. 1). The benzene-pyridine arms are tilted at 20.68° from each other, with a centroid–centroid distance of 7.09 Å for two aromatic planes (Supplementary Fig. 28). The nitrogen atoms separated from the pyridyl groups by a distance of 10.18 Å (Supplementary Fig. 28), thereby making **3** suitable for the coordination-driven self-assembly with a 90° Pt(II) acceptor and carboxylate ligands. The torsion angle between the naphthyridine ring planes is 120.51°, and the nitrogen atoms are oriented in the interior of the cavity, placing potential hydrogen bond binding sites inside the binding pocket. Also, one DCM molecule is found in the cavity (Fig. 1c), which suggests a solid-state DCM ⊂ **3** host-guest complex with multiple C–H···N hydrogen bonds between the H atoms of the DCM and the N atoms from naphthyridyl groups. The solid-state structure indicates that **3** can be a building block for SCCs, with endo-functionalized hydrogen bond binding sites within the coordination framework and leads to potential applications in host-guest systems^38–40^ and interlocked structures^21–27^.

### Self-assembly of bis-metallacage/bis[2]catenane

**Fig. 2:**
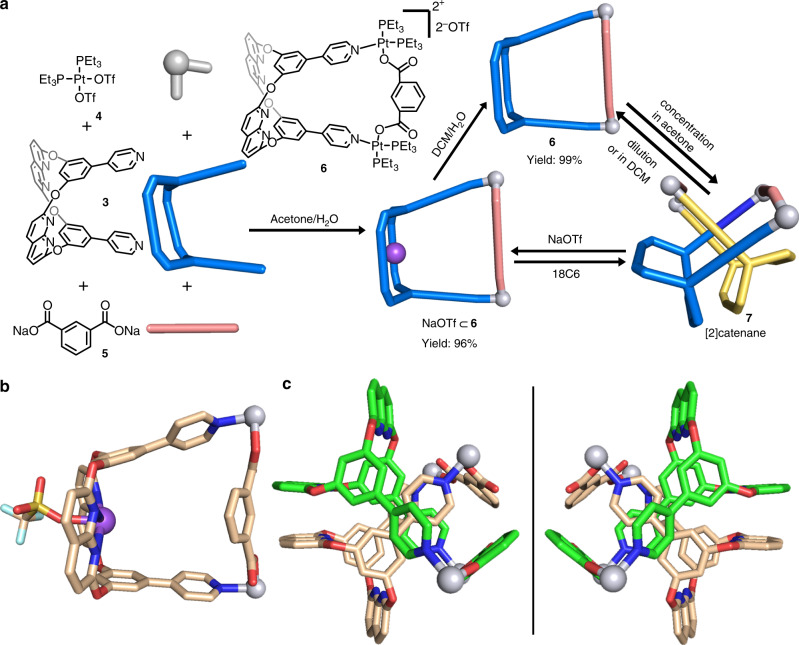
Self-assembly, reversible transformations, and single-crystal structures of metallacage 6, NaOTf ⊂ 6, and 7. **a** Self-assembly and reversible transformations of metallacage **6**, NaOTf ⊂ **6** and [2]catenane **7**. **b** Single crystal structure of NaOTf ⊂ **6** (hydrogen atoms, PEt_3_ groups and some of OTf anions are omitted for clarity). **c** Single crystal structure of enantiomers of [2]catenane **7** (hydrogen atoms, PEt_3_ groups and OTf anions are omitted for clarity).

**Fig. 3:**
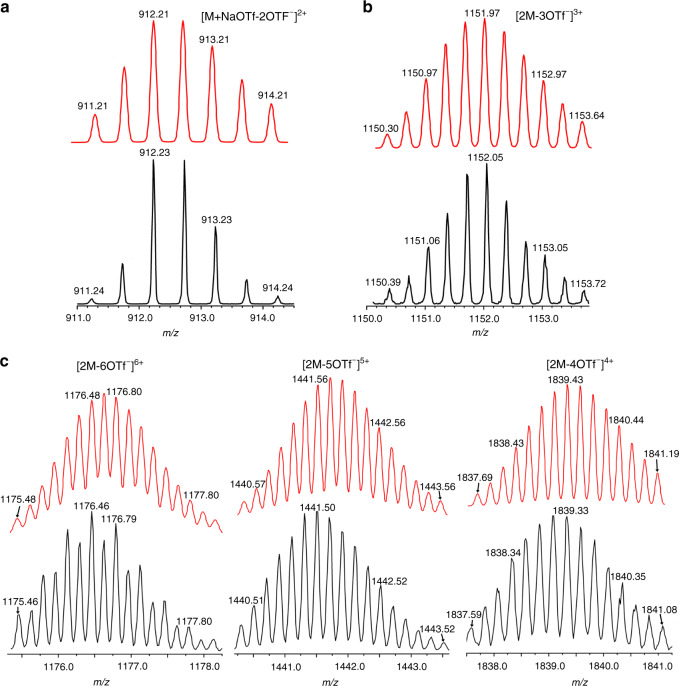
ESI-TOF-MS spectra of NaOTf ⊂ 6, 6/7 and 9/10. **a** Experimental (black) and calculated (red) ESI-TOF-MS spectra of NaOTf ⊂ **6** ([M + NaOTf-2OTf^−^]^2+^). **b** Experimental (black) and calculated (red) ESI-TOF-MS spectra of **7** ([2M-3OTf^−^]^3+^). **c** Experimental (black) and calculated (red) ESI-TOF-MS spectra of **10**. For full spectra, see Supplementary Figs. 16, 21 and 26.

A bis-metallacage or cage-based [2]catenane oligomer might be formed by 90° Pt(II) heteroligation^34^ of ligand **3** and a tetra-carboxylated linker. To test this postulate, we first investigate the self-assembly of a simple mono-metallacage or [2]catenane through 90° Pt(II) heteroligation of the ligand **3** and the dicarboxylate ligand **5**. As shown in Fig. 2, a mixture of **3**, *cis*-(PEt_3_)_2_Pt(OTf)_2_ **4** (OTf, OSO_2_CF_3_), and dicarboxylate ligand **5** in a 1:2:1 ratio in acetone/H_2_O (v/v = 5:1) was expected to assemble endo-functionalized metallacage **6** or [2]catenane **7**. The ^31^P{^1^H} and ^1^H NMR spectra (Supplementary Figs. 14 and 15) as well as a DOSY NMR spectrum (*D* = 9.00 × 10^−10^ m^2^ s^−1^, Supplementary Fig. 17) of the mixture indicate a single, discrete assembly with high symmetry. The ^31^P{^1^H} spectrum has two sets of coupled doublets (δ = 6.96 and 1.66 ppm, ^2^*J*_P−P_ = 21.3 Hz), with concomitant ^195^Pt satellites that correspond to two distinct phosphorus environments. This indicates that the Pt(II) center possesses a heteroligated coordination moiety with both carboxylate and pyridyl moieties and breaks the symmetry of the two capping phosphine ligands^34^. The electrospray ionization time-of-flight mass spectrometry (ESI-TOF-MS) of **6** showed a [M – 2OTf^−^]^2+^ peak that corresponds to the [1 + 2 + 1] assembly with charge states resulting from the loss of the OTf counterions (Supplementary Fig. 16). The ESI-TOF-MS also suggests the presence of a by-product, NaOTf, coordinated with **6** (NaOTf ⊂ **6**), which has a peak of [M + NaOTf – 2OTf^−^]^2+^ (Fig. 3a).

Single crystals of NaOTf ⊂ **6** suitable for X-ray diffraction analysis were grown by the slow diffusion of diethyl ether into an acetone solution of NaOTf ⊂ **6**. As shown in Fig. 2b, the X-ray data unambiguously confirms the structure of **6**, where NaOTf is coordinated in the middle of two naphthyridyl groups. Four coordination bonds exist between the naphthyridyl groups and Na^+^ with Na···N distances of 2.62, 2.66, 2.67 and 2.68 Å, respectively. Moreover, the angle between two naphthyridine ring planes is 50.58^o^ due to the presence of the NaOTf. The distance between the two N atoms in the pyridyl groups of **3** is 9.59 Å, as a result of the 90^o^ Pt(II) heteroligation^41^.

By dissolving the NaOTf ⊂ **6** in DCM and extracting the NaOTf with water, free **6** without NaOTf was obtained. The ^31^P{^1^H} spectra (Supplementary Fig. 34) of NaOTf ⊂ **6** and free **6** in CD_2_Cl_2_ have similar sets of coupled doublets, indicating that the self-assembly framework remains after extraction. Moreover, the proton signals of the naphthyridyl groups exhibit an upfield shift in the NMR due to the removal of NaOTf (Supplementary Fig. 35, ∆δ = −0.08 and −0.07 ppm, respectively).

Single crystals of metallacage **6** were obtained by the slow diffusion of isopropyl ether into an acetone solution of **6**. The crystal structure had only one metallacage without NaOTf. However, the interlocked structure of two metallacages ([2]catenane, **7**) was also observed after symmetry operations. As shown in Fig. 2c, an endo-functionalized cavity of each cage was penetrated by the benzene-pyridine arm of the partner cage, and a pair of enantiomers of **7** was found in the crystal (Fig. 2c). Moreover, two aryl ring planes of dicarboxylate ligands were nearly perpendicular to each other, with a torsion angle of 88.44°. Multiple C–H···N hydrogen bonds between H atoms from the aromatic rings and the N atoms from the naphthyridyl groups were observed (Supplementary Fig. 30), with C–H···N distances from 2.82 to 3.01 Å. The efficient π···π stacking between two of four naphthyridine planes was also found with a centroid–centroid distance of 3.59 Å (Supplementary Fig. 31). Although the locking process is entropically disfavored, these supramolecular interactions between monomers act as the main energy source to overcome the entropy loss during the catenation. The crystal structures of NaOTf ⊂ **6** and **7** suggested that the endo-functionalized cavity of **6** may dominate the formation of catenane species, which was further supported by chemical-stimuli-responsive control experiments in solution, as described below. Furthermore, ESI-TOF-MS suggested that the catenated dimer **7** also exists in the acetone solution of **6**, and the results were in good agreement with its theoretical distributions ([2 M – 3OTf^−^]^3+^, Fig. 3b).

**Fig. 4:**
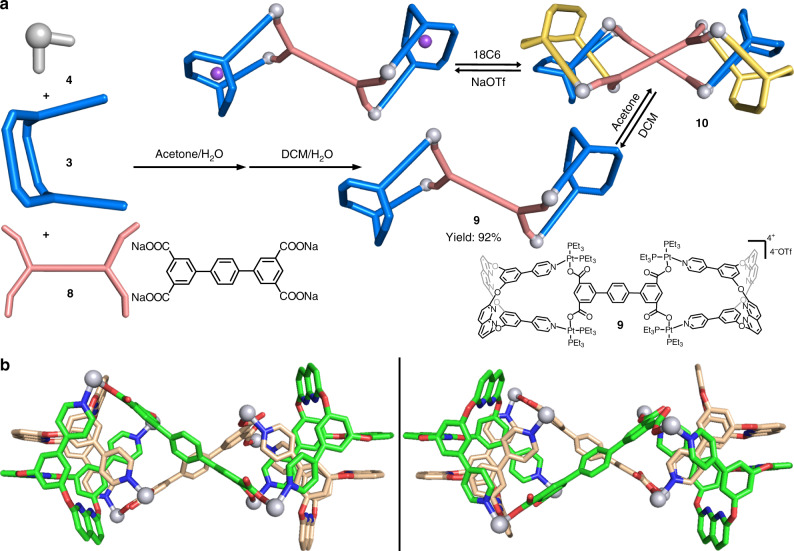
Self-assembly, reversible transformations, and single-crystal structures of 9 and 10. **a** Self-assembly and reversible transformations of bis-metallacage **9** and **10**. **b** Single crystal structure of enantiomers of **10**. (PEt_3_ groups are omitted for clarity).

The generation of **7** motivated us to investigate if a tetra-carboxylated ligand could lead to the self-assembly of a bis-metallacage, which may further assemble into more complicated frameworks (such as poly-[2]catenane or cyclic [2]catenane oligomers). As shown in Fig. 4a, bis-metallacage **9** was assembled from **3**, 90° Pt(II) acceptor **4**, and tetra-carboxylate ligand **8** in a ratio of 2:4:1, and the by-product NaOTf was removed from the system by extraction. The ESI-TOF-MS for **9** in acetone shows three peaks ([2 M–4OTf^−^]^4+^, [2 M–5OTf^−^]^5+^ and [2 M–6OTf^−^]^6+^) which correspond to those attributed to the dimer **10** formation, with charge states resulting from the loss of OTf counterions (Fig. 3c and Supplementary Fig. 26). Moreover, the solid-state structure of **10** was confirmed by single-crystal X-ray diffraction analysis and is a cyclic bis[2]catenane with “∞”-shaped topology (Fig. 4b).

The X-ray diffraction data for single crystals of **10** only reflected a 1.5 Å resolution due to size and complexity of the compound. This issue was addressed by using the rigid body approach^42–44^. The solid-state structure of **9** shows two independent bis-metallacages in different conformations (**I** and **II**, see supplementary Fig. 32), with different Pt···Pt distances (15.28 and 10.73 Å; 11.53 and 17.03 Å, respectively) between the two metallacage frameworks in the bis-metallacage. After symmetry operation, a pair of enantiomers of **10** was formed as cyclic bis[2]catenane with “∞”-shaped topology. Each bis[2]catenane has a conformation **I** and a conformation **II** of **9**, corresponding to a 14-component self-assembly. As shown in Fig. 4b, two [2]catenane frameworks in bis[2]catenane were interlocked in a similar way as those in the single [2]catenane, as previously discussed, and linked to each other by two tetra-carboxylate linkers with an oriented skew line shape^45^. To the best of our knowledge, this is the first example of a [2]catenane oligomer formed from three-dimensional cages.

### Reversible structural transformations

Next, we investigated the chemical-stimuli-responsive reversible structural transformations between [2]catenane/bis[2]catenane and metallacage/bis-metallacage in solution by ^31^P {^1^H} NMR and ^1^H NMR spectra. The self-assembly of **6** was concentration-dependent. When we evaluated the NMR spectra of **6** at low concentration (2.0 mM, acetone-*d*_6_), ^31^P{^1^H} NMR (Supplementary Fig. 36) and ^1^H NMR (Supplementary Fig. 37) spectral analyses revealed the formation of monomeric metallacage **6**, as evidenced by the similarities between the NMR spectra of **6** and NaOTf ⊂ **6**. The proportion of **6** at 2.0 mM was calculated to be 89.3% (Supplementary Table 6). Increasing the concentrations of **6** in acetone-*d*_6_ from 2.0 to 30 mM led to gradual transformation of the metallacage into catenane **7**. For example at the concentration of 30 mM, the percentage of **7** reached 81.8% (Supplementary Table 6). At 30 mM, at least four sets of coupled doublets are observed by the ^31^P{^1^H} NMR spectrum (Supplementary Fig. 36), which corresponds to the four distinct phosphorus environments of **7**. In the DOSY spectrum (Supplementary Fig. 55), interlocked **7** showed lower diffusion coefficient (*D*) of 5.70 × 10^−10^ m^2^ s^−1^ than that for monomeric **6** (6.73 × 10^−10^ m^2^ s^−1^), also indicating the assembled catenation. The ^1^H NMR (Supplementary Fig. 37) spectrum exhibited complex spectra at high concentration, the high-field shift^17^ of the pyridyl proton at δ = 5.97 ppm is characteristic of the inside unit due to the inclusion-induced shielding effect. That is, at lower concentrations, the chemical equilibrium leans toward the monomeric metallacage **6**, however, at higher concentrations, **7** is the dominant species in solution in accord with the Le Chatelier principle.

In contrast, bis-matallacage **9** tends to assemble into bis[2]catenane **10** even at very low concentrations. At 2.0 mM in acetone-*d*_6_, for example, the ^31^P{^1^H} NMR (Supplementary Fig. 44) and the ^1^H NMR (Supplementary Fig. 45) spectra suggest the formation of the asymmetrical catenated structure (**10**, 77.7%, Supplementary Table 9), with high-field shift of the proton signal at δ = 6.21 ppm and four sets of coupled doublets of phosphine signals. That is, bis[2]catenane **10** could be formed at a much lower concentration comparing with the monomeric [2]catenane **7**. This is reasonable due to the synergistic effect of two catenated metallacages in **10**. At higher concentration of 15 mM, the percentage of **10** increased to 94.9% (Supplementary Table 9), with a *D* value of 4.42 × 10^−10^ m^2^ s^−1^ (Supplementary Fig. 56).

**Fig. 5:**
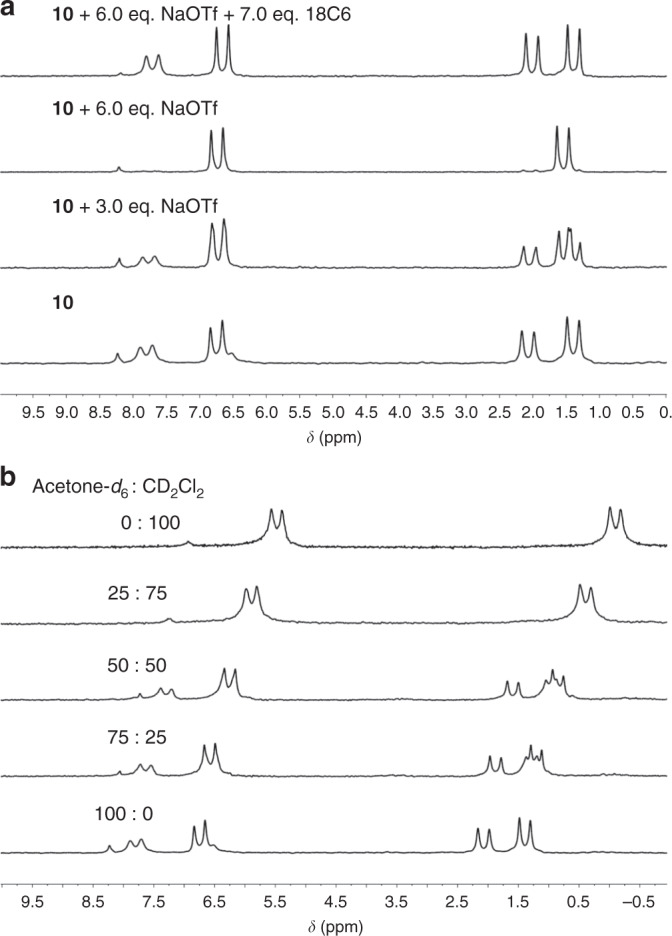
Partial ^31^P{^1^H} NMR spectra (121 MHz, 298 K). **a** ^31^P{^1^H} NMR spectra of **10** in acetone-*d*_6_ in the absence and presence of NaOTf and 18C6. **b** ^31^P{^1^H} NMR spectra of **10** in a mixing solvent of acetone-*d*_6_/CD_2_Cl_2_ (v/v). The concentrations are 15 mM for bis-matallacage **9**.

To understand the role of endo-functionalized naphthyridyl groups in the formation of the interlocked [2]catenane/bis[2]catenane, NaOTf was chosen as a competitive guest to block the endo hydrogen bond binding sites. In the synthesis of **6**, a complex with NaOTf was yielded (Fig. 2), suggesting their strong complexation. From ^1^H NMR titration experiment, the association constant was determined to be (1270 ± 30) M^−1^ in acetone-*d*_6_ (Supplementary Figs. 59 and 60). Upon addition of 6.0 equivalent (eq.) of NaOTf, the ^31^P{^1^H} NMR (Fig. 5a and Supplementary Fig. 46) spectra of **10** (15 mM for bis-matallacage **9**) show a clear transformation from four sets of coupled doublets for **10**, into two sets of coupled doublets for 2NaOTf ⊂ **9**. And the ^1^H NMR (Supplementary Fig. 47) spectra also indicate a structural transformation from **10** to 2NaOTf ⊂ **9** (Supplementary Table 10). Instead, the addition of 3.0 eq. NaOTf could not completely transform **10** to 2NaOTf ⊂ **9** due to the stronger stability of the cyclic bis[2]catenane stuctures. The complexation towards sodium cations inhibits the C-H···N hydrogen bonding and π···π stacking interactions in the interlocked structures, and therefore dissembles the bis[2]catenanes with high fidelity. Then, we expected that the Na^+^ can be removed from the cavities of **9** with the assistance of 18-crown-6-ether (18C6). The removal of Na^+^ could make naphthyridyl moieties available for hydrogen-bonding receptors, resulting in the re-assembly of catenane **10**. When we added 7.0 eq. 18C6 to the mixture of **9** and NaOTf, the NMR signals almost recovered the resonances of **10** (Fig. 5a and Supplementary Fig. 48), indicating that the NaOTf- and 18C6-triggered structural inversions are reversible (Fig. 4a). The reversible structural transformation between [2]catenane **7** and mono-metallacage **6** can be realized in a similar way. Comparing with bis[2]catenane **10**, smaller amount of NaOTf (1.5 eq.) can completely transform [2]catenane **7** into NaOTf ⊂ **6**, which is reasonable because there is no synergistic effect in the formation of **7** (Supplementary Figs. 38–41 and Supplementary Table 7).

We also studied the solvent-induced structural transformations between **10** and **9** (Fig. 4a). When the proportion of CD_2_Cl_2_ in an acetone-*d*_6_/CD_2_Cl_2_ solution of **10** increase, the ^31^P{^1^H} NMR (Fig. 5b and Supplementary Fig. 49) and ^1^H NMR (Supplementary Fig. 50) spectra clearly show that the nuclear resonances of **10** are gradually replaced by the signals for **9**. While **9** is dissolved in CD_2_Cl_2_ at 15 mM, only the resonances for the bis-metallacage **9** is observed by NMR spectroscopy (Fig. 5b and Supplementary Fig. 50), indicating that catenated metallacages could not be formed in CD_2_Cl_2_. DCM may play a role of a competitive guest to block the cavities of **9** from interpenetration, as evidenced by the DCM ⊂ **3** host-guest complexes observed in the solid-state structure (Fig. 1c). The solvent-induced transformations were also found between monomeric [2]catenane **7** and metallacage **6** in the CD_2_Cl_2_/acetone-*d*_6_ solution (Supplementary Figs. 42 and 43). Furthermore, we examined the catenation in other solvents. [2]Catenane **7** and bis[2]catenane **10** are preferentially formed in methanol-*d*_4_ (Supplementary Figs. 51–54, Supplementary Tables 8 and 11). However, in highly dipolar solvents such as acetonitrile-*d*_3_ and DMSO-*d*_6_, cages **6** and **9** are the dominated products due to the inhibition of hydrogen bonds and complementary π–π-stacking through efficient solvation of the cages (Supplementary Figs. 51–54, Supplementary Tables 8 and 11).

## Discussion

In summary, a novel “∞”-shaped cyclic bis[2]catenane metallacage was successfully formed by Pt(II) heteroligation, as evidenced by NMR spectrometry, single crystal X-ray diffraction and mass spectrometry. The endo-functionalized double-bridged tweezer **3** provided suitable cavity size (7.09 Å height) and multiple hydrogen bond binding sites (naphthyridyl moieties) in the metallacage frameworks, which play a dominant role in promoting the formation of the complicated interlocked structure. The responsive disassembly and reassembly processes of the interlocked structure were investigated in solution; guest exchange, solvent effect, and concentration effect facilitated the reversibility of the self-assembly. The ability to assemble the cyclic bis[2]catenane structure suggested that the present strategy may have a role in the future construction of complex interlocked architectures and smart materials. For example, the use of triangular or rhombic carboxylated ligands would produce catenated structures with significantly high complexity. Also, the introduction of stimuli-responsive moieties to carboxylated ligands could afford interlocked materials those are responsive to light-irradiation, heating or mechanical stress. Studies directed to exploring these possibilities are underway.

## Supplementary information

Supplementary Information

## Data Availability

The X-ray crystallographic coordinates for structures reported in this study have been deposited at the Cambridge Crystallographic Data Centre (CCDC), under deposition numbers 1939272, 1939276, 1946245, and 1947413. These data can be obtained free of charge from The Cambridge Crystallographic Data Centre via www.ccdc.cam.ac.uk/data_request/cif. The authors declare that the data supporting the findings of this study are available within the paper and its Supplementary Information. All data are available from the authors on reasonable request.
