# Supplementary material for: A cyclic bis[2]catenane metallacage
Source: Nat Commun. 2020 Jun 1;11:2727. doi: 10.1038/s41467-020-16556-3 (PMC7264200; doi:10.1038/s41467-020-16556-3)
Supplement: Supplementary file 1 — Supplementary Information [file 41467_2020_16556_MOESM1_ESM.pdf]

Supplementary Information

A cyclic Bis[2]catenane Metallacage

Wang et al.

Supplementary Methods

All reagents were commercially available and used as supplied without further purification. Deuterated solvents were purchased from Cambridge Isotope Laboratory (Andover, MA). Compound **1**[1], **4**[2], *p*-Terphenyl-3,3'',5,5''-tetracarboxylic acid[3], 1-iodo-3,5-dimethoxy-benzene[4] was prepared according to the published procedures. ^{1}H NMR, ^{13}C NMR NMR spectra were recorded on a Varian Inova 400 MHz spectrometer and JEOL JNM-ECZ400SL NMR spectrometer. ^{31}P{^{1}H} NMR spectra were recorded on a Varian Unity 300 MHz spectrometer and JEOL JNM-ECZ400SL NMR spectrometer. DOSY spectra were recorded on a Varian Inova 500 MHz spectrometer and Bruker AVANCE III HD 500MHz spectrometer. COSY spectra were recorded on a Bruker AVANCE III HD 600MHz spectrometer. ^{1}H and ^{13}C NMR chemical shifts are reported relative to residual solvent signals, and ^{31}P{1H} NMR chemical shifts are referenced to an external unlocked sample of 85% H_3PO_4 (δ = 0.0). Mass spectra were recorded on a Micromass Quattro II triple-quadrupole mass spectrometer using electrospray ionization with a MassLynx operating system. The single crystals data were collected on an Oxford Diffraction Xcalibur Atlas Gemini captra.

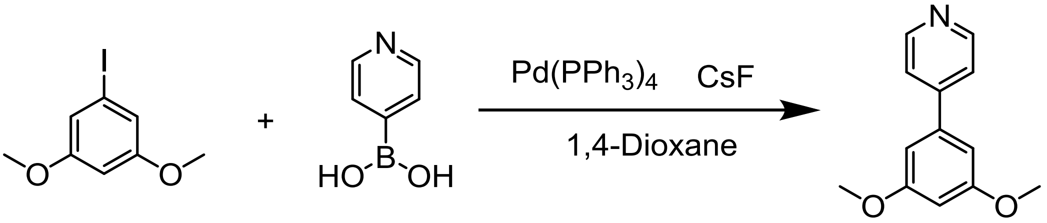

Supplementary Figure 1. The synthesis of 4-(3,5-dimethoxyphenyl)-pyridine.

Synthesis of 4-(3,5-dimethoxyphenyl)-pyridine. 1-iodo-3,5-dimethoxy-benzene (2.64 g, 10 mmol), 4-Pyridineboronic acid (1.48 g, 12 mmol), CsF (3 g) and tetrakis(triphenylphosphine)palladium(0) (300 mg) were mixed in a 250 mL flask connected to a condenser. The flask was degassed under vacuum for 2 h, and then 100 mL of degassed 1,4-dioxane were added to the flask. The reaction mixture was heated to reflux under nitrogen atmosphere for 48 h. After removal of the organic solvent under vacuum, the residue was washed with water and extracted with CH_2Cl_2 three times. Combined organic layers were dried over $MgSO_4$ and filtered. The solvent was removed and the crude product was purified by column chromatography on silica gel (ethyl acetate/hexane: 1/5) to give 1.25 g 4-(3,5-dimethoxyphenyl)-pyridine as a white solid. Yield: 58%. The ^{1}H NMR spectrum of 4-(3,5-dimethoxyphenyl)-pyridine is shown in Supplementary Figure 2. ^{1}H NMR (400 MHz, $CDCl_3$, 298 K) δ (ppm): 8.64 (d, J = 5.4 Hz, 2H), 7.46 (d, J = 6.0 Hz, 2H), 6.74 (d, J = 2.2 Hz, 2H), 6.53 (t, J = 2.3 Hz, 1H), 3.84 (s, 6H). The ^{13}C NMR spectrum of 4-(3,5-dimethoxyphenyl)-pyridine is shown in Supplementary Figure 3. ^{13}C NMR (100 MHz, $CDCl_3$, 298 K) δ (ppm): 161.45, 150.31, 148.48, 140.43, 121.84, 105.40, 100.84, and 55.57. HRMS (ESI-ToF) m/z: $[M+H]^+$ Calc'd for $[C_{13}H_{14}NO_2]^+$, 216.1019; Found 216.1025.

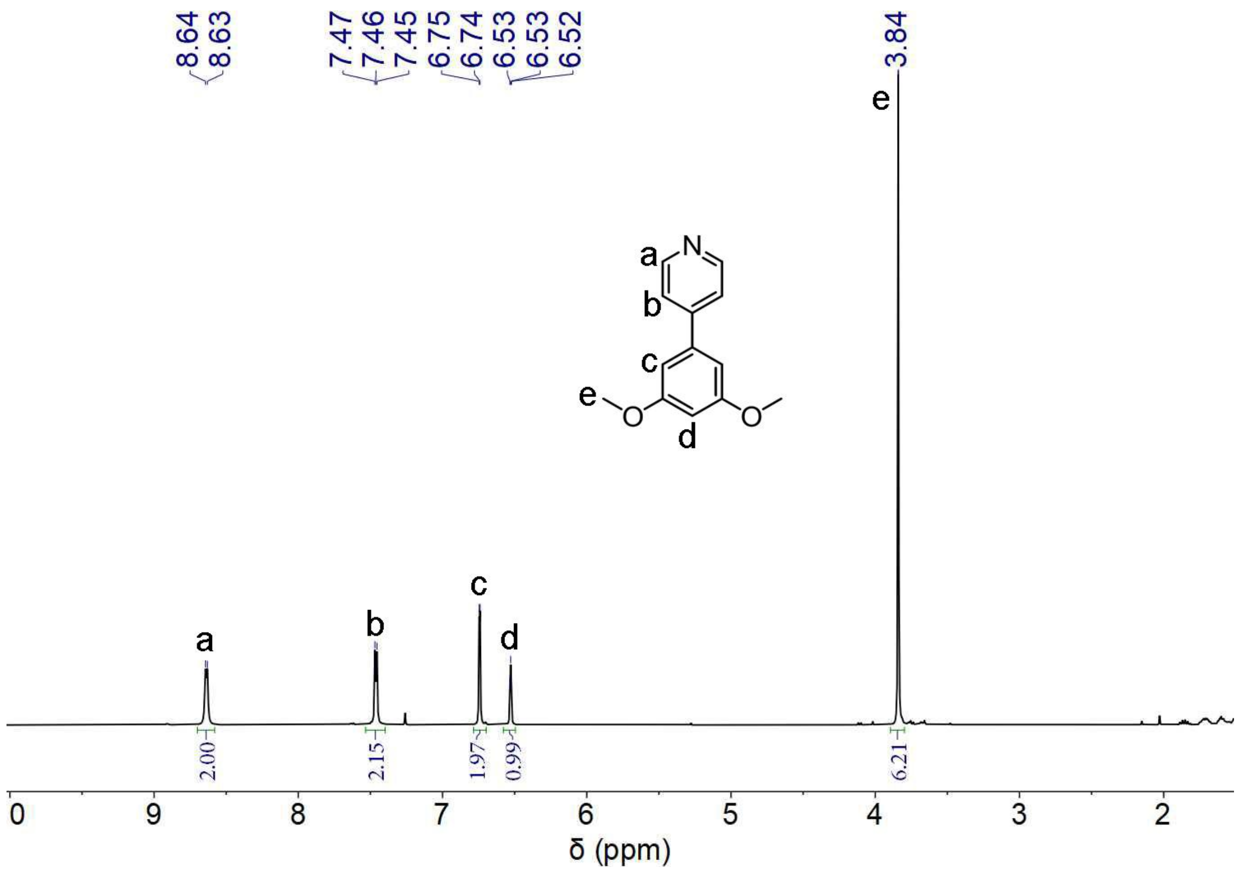

Supplementary Figure 2. ^{1}H NMR spectrum (400 MHz, $CDCl_3$, 298K) of 4-(3,5-dimethoxyphenyl)-pyridine.

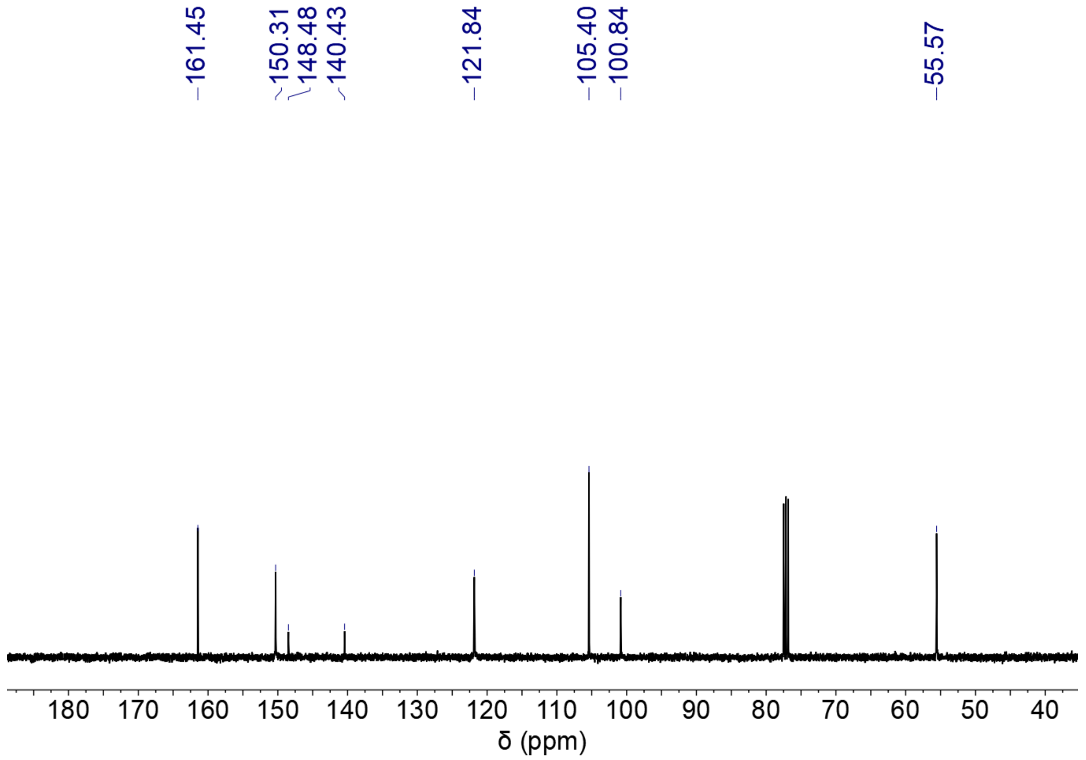

Supplementary Figure 3. ^{13}C NMR spectrum (100 MHz, $CDCl_3$, 298K) of 4-(3,5-dimethoxyphenyl)-pyridine.

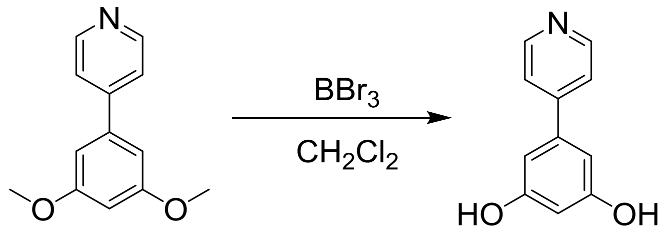

2

Supplementary Figure 4. The synthesis of compound **2**.

Synthesis of 2. Boron tribromide (3 mL) was added to a CH_2Cl_2 (20 mL) solution of 4-(3,5-dimethoxyphenyl)-pyridine (1.08 g, 5 mmol). The mixture was stirred at room temperature for 6 h. Then the mixture was poured into ice water. Combined aqua layers and concentrated, then the pH value of **2**·HBr solution was adjusted to 7.0 with standard sodium hydroxide solution. The resulting precipitate was collected by filtration and washed with water to give 870 mg **2** as pale-yellow solid, yield: 93%. The ^{1}H NMR spectrum of **2** is shown in Supplementary Figure 5. ^{1}H NMR (400 MHz, DMSO-d_6, 298 K) δ (ppm): 9.97 (s, 2H), 9.03 (s, 2H), 7.99 (s, 2H), 7.01 (d, J = 2.1 Hz, 2H), 6.76 (t, J = 2.1 Hz, 1H). The ^{13}C NMR spectrum of **2** is shown in Supplementary Figure 6. ^{13}C NMR (100 MHz, DMSO-d_6, 298 K) δ (ppm): 159.50, 150.36, 148.05, 139.51, 121.63, 105.28, and 103.78. HRMS (ESI-ToF) m/z: $[M+H]^+$ Calc'd for $[C_{11}H_{10}NO_2]^+$, 188.0706; Found 188.0712.

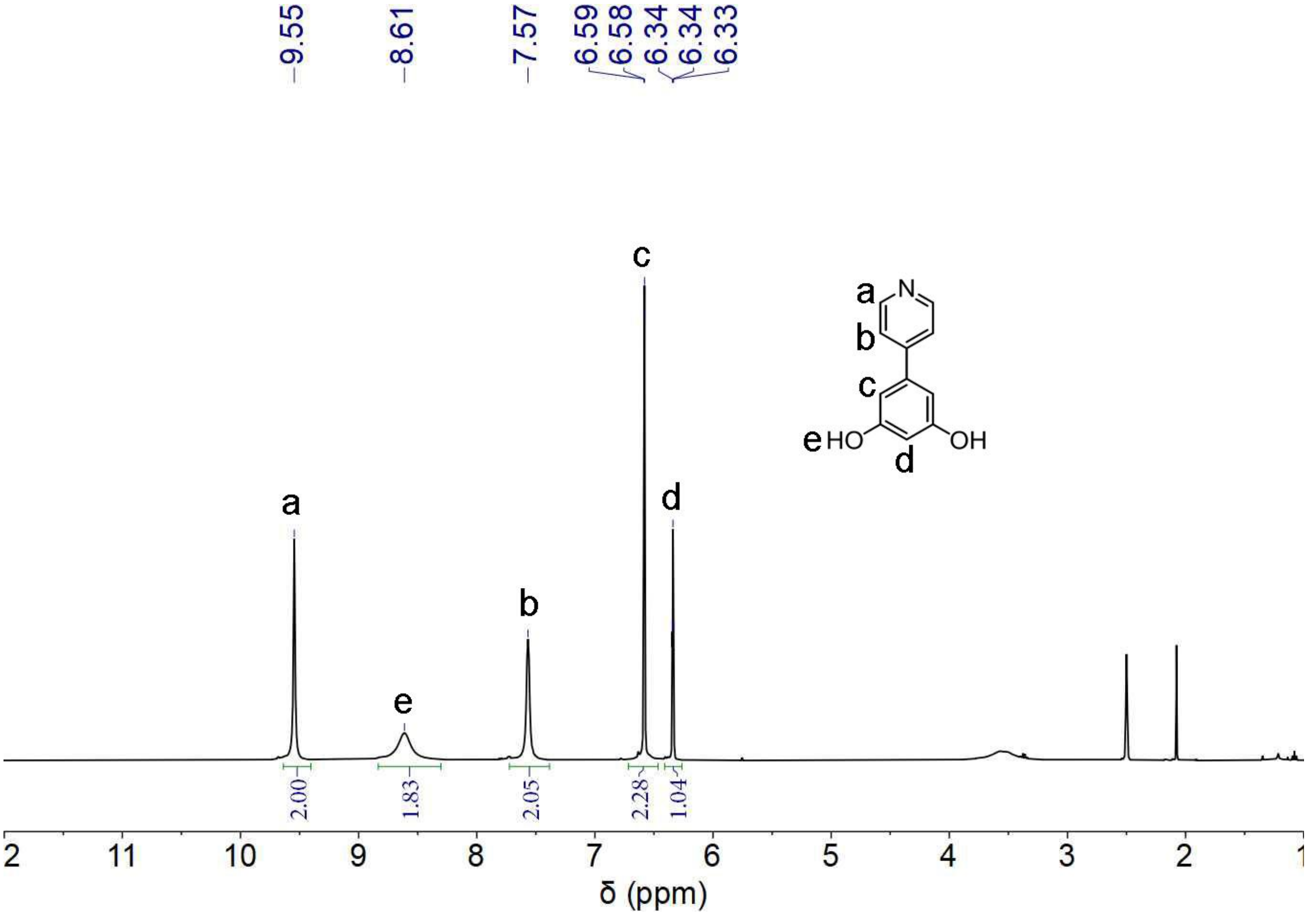

Supplementary Figure 5. ^{1}H NMR spectrum (400 MHz, DMSO-d_6, 298K) of compound **2**.

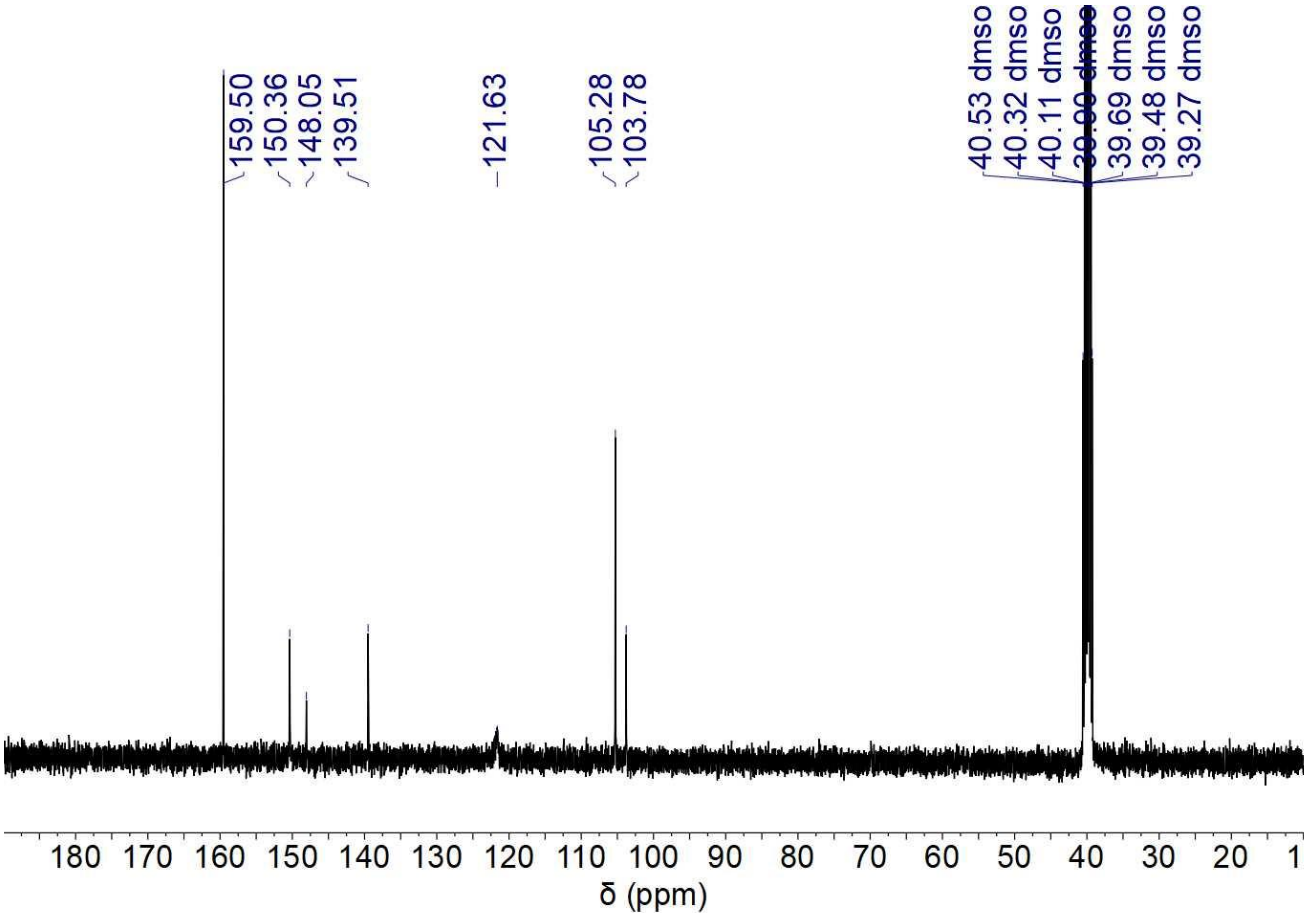

Supplementary Figure 6. ^{13}C NMR spectrum (100 MHz, DMSO-d_6, 298K) of compound **2**.

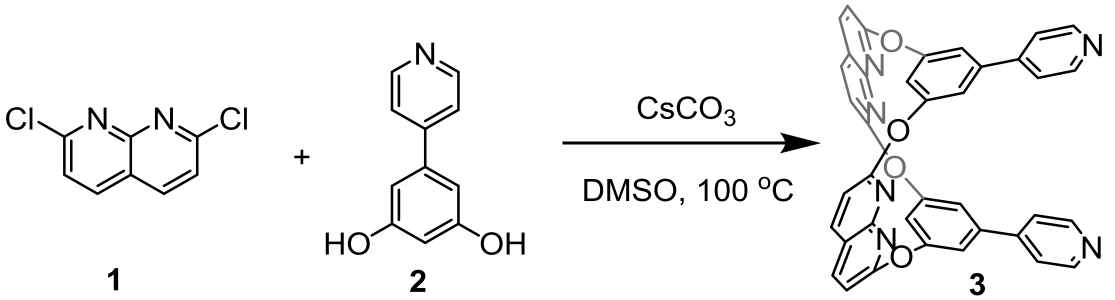

Supplementary Figure 7. The synthesis of compound **3**.

Synthesis of 3. 2,7-Dichloro-1,8-naphthyridine (**1**) (1.33 g, 6.7 mmol, 1 equivalent (eq.)), **2** (1.25 g, 6.7 mmol, 1 eq.), and Cs_2CO_3 (5.45 g, 16.7 mmol, 2.5 eq.) were combined under an nitrogen atmosphere. DMSO (100 mL) was added, and the reaction was heated to 100 ^{o}C and stirred vigorously for 18 hr. The reaction mixture was diluted with 200 mL H_2O causing formation of a grey precipitate. The solid was collected by filtration, washed with 100 mL H_2O, dissolved in CH_2Cl_2, dried over anhydrous $MgSO_4$, filtered, and concentrated in vacuum. The product was then purified via column chromatography by silica gel (ethyl acetate) and recrystallization in CH_2Cl_2/hexane to yield 1.34 g **3** as a pale-yellow solid. Yield: 64%. The ^{1}H NMR spectrum of **3** is shown in Supplementary Figure 8. ^{1}H NMR (400 MHz, DMSO-d_6, 298 K) δ (ppm): 8.55 (d, J = 5.8 Hz, 4H), 8.44 (d, J = 8.6 Hz, 4H), 7.71 (d, J = 5.9 Hz, 4H), 7.56 (d, J = 1.9 Hz, 4H), 7.30 (d, J = 8.6 Hz, 4H), 7.17 (t, J = 1.9 Hz, 2H). The ^{13}C NMR spectrum of **2** is shown in Supplementary Figure 9. ^{13}C NMR (100 MHz, DMSO-d_6, 298 K) δ (ppm): 164.20, 154.39, 153.30, 150.02, 145.21, 140.77, 140.06, 121.33, 118.71, 117.57, 116.81 and 111.16. HRMS (ESI-ToF) m/z: $[M+H]^+$ Calc'd

for $[C_{38}H_{23}N_6O_4]^+$, 627.1775; Found 627.1783.

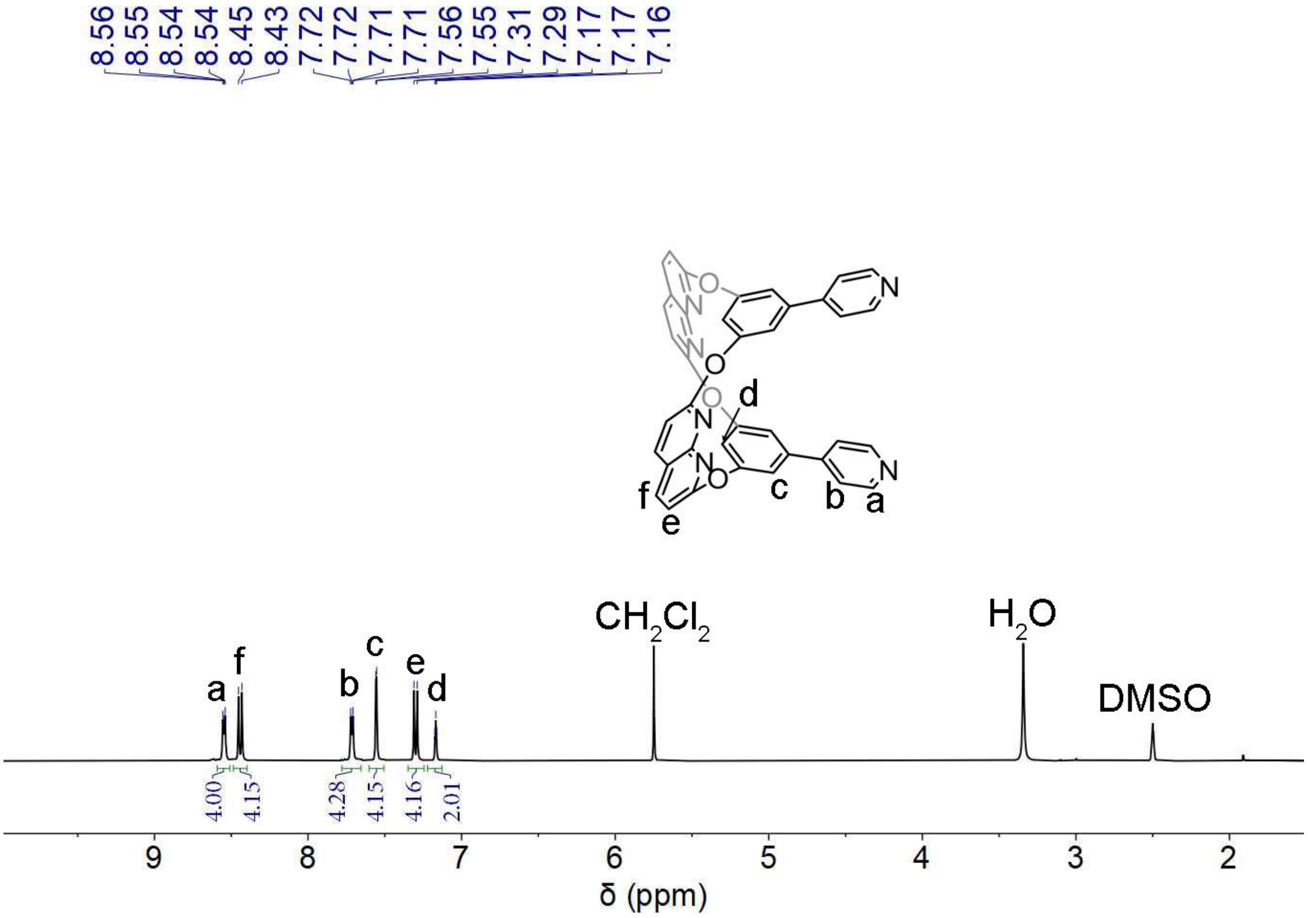

Supplementary Figure 8. ^1H NMR spectrum (400 MHz, DMSO-d_6, 298K) of compound **3**.

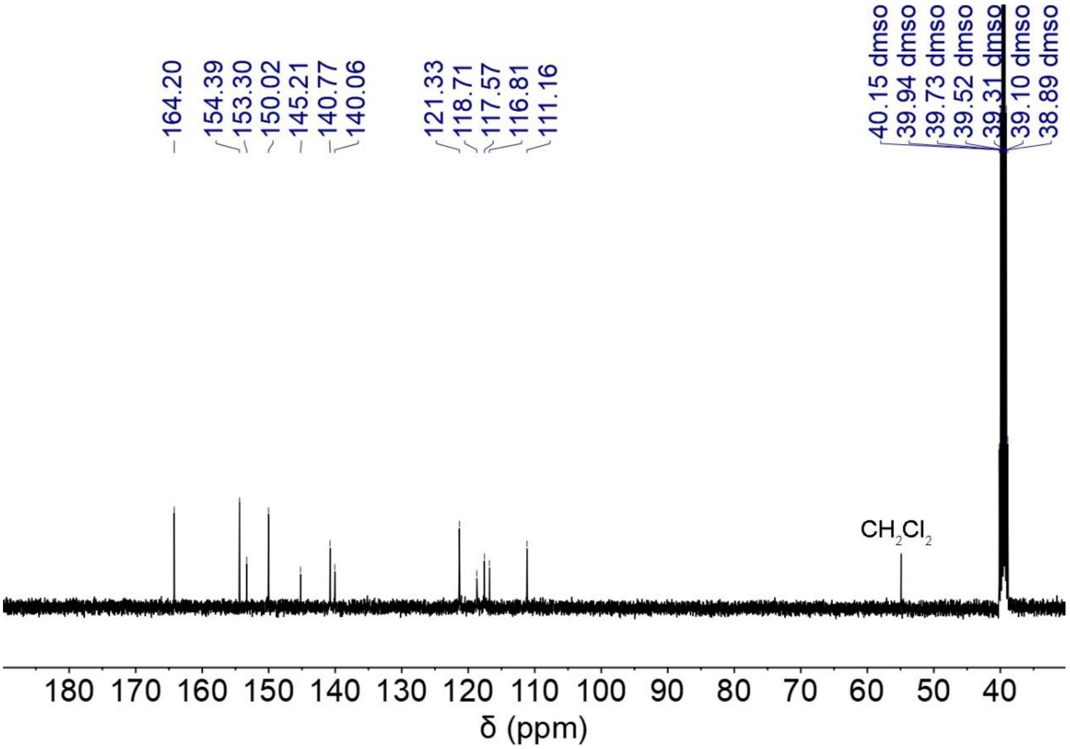

Supplementary Figure 9. ^{13}C NMR spectrum (100 MHz, DMSO-d_6, 298K) of compound **3**.

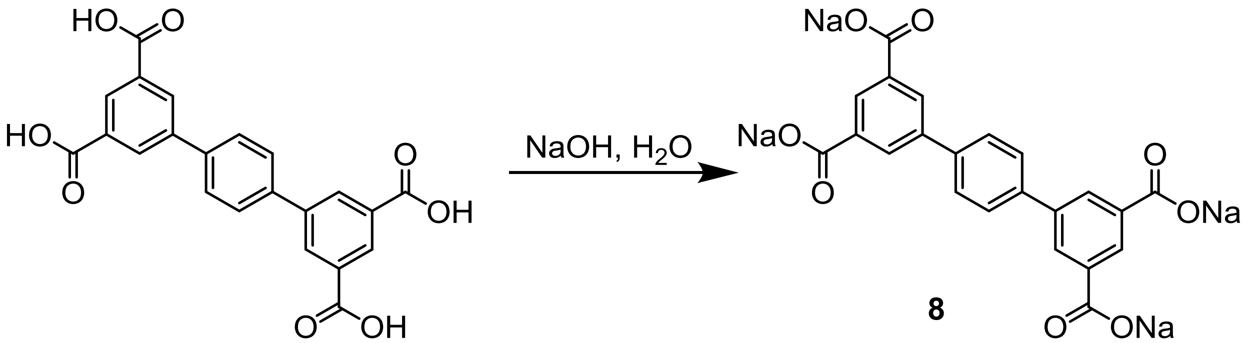

8

Supplementary Figure 10. The synthesis of compound **8**.

Synthesis of 8. *p*-Terphenyl-3,3'',5,5''-tetracarboxylic acid (68 mg, 0.17 mmol) and NaOH (27 mg, 0.67 mmol) were added into a 2-dram vial with 5 mL water. The mixture was stirred at room temperature for 3 h. All solvent was removed by N_2 flow and white solid **7** was dried under vacuum (79 mg, Yield: 99%). The ^{1}H NMR spectrum of **8** is shown in Supplementary Figure 11. ^{1}H NMR (400 MHz, D_2O, 298 K) δ (ppm): 8.36 (t, *J* = 1.3 Hz, 4H), 8.31 (q, *J* = 1.4 Hz, 2H), 7.96 (d, *J* = 1.0 Hz, 4H). The ^{13}C NMR spectrum of **8** is shown in Supplementary Figure 12. ^{13}C NMR (100 MHz, D_2O, 298 K) δ (ppm): 174.94, 139.87, 139.22, 137.17, 129.57, 128.01, and 127.64.

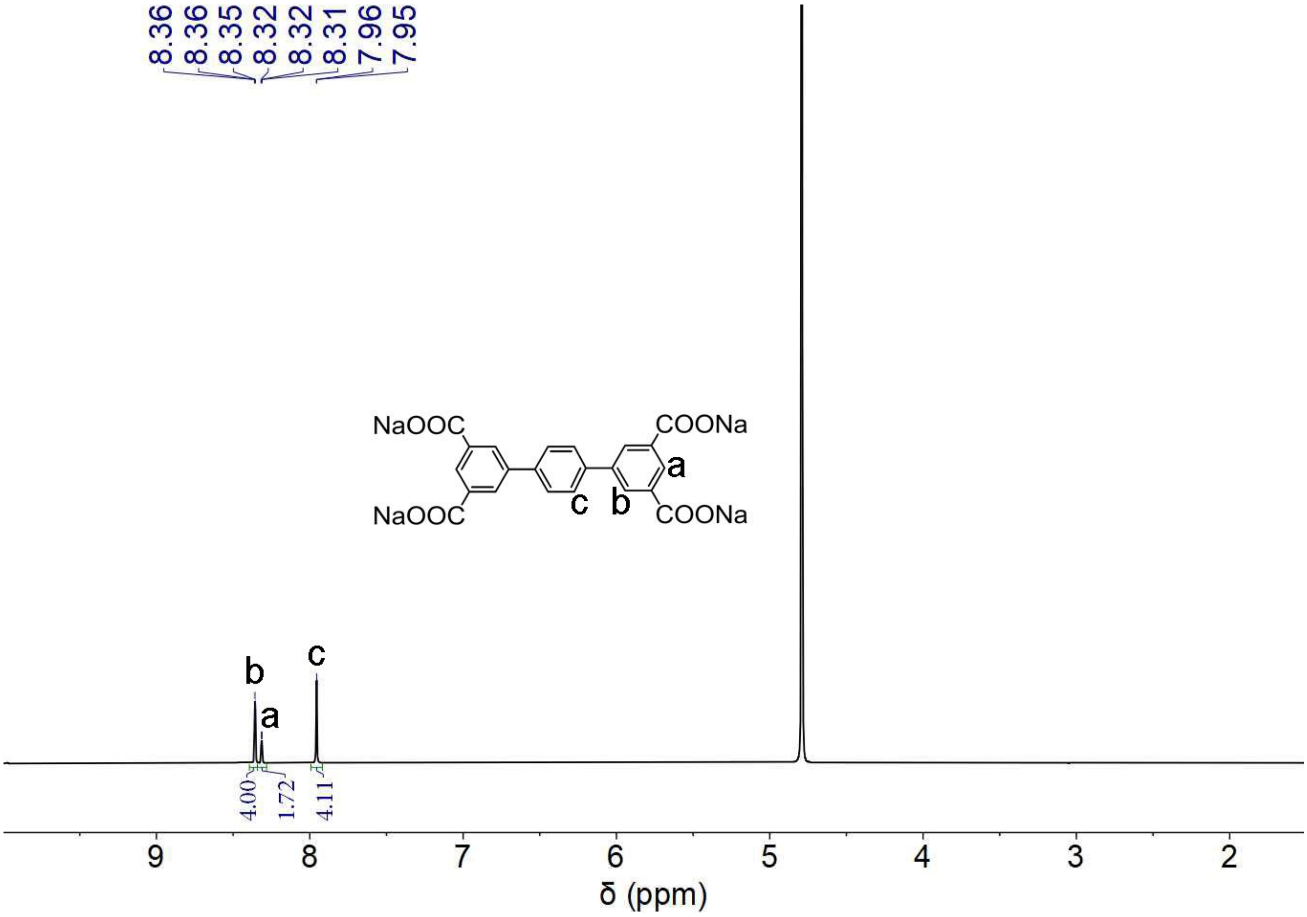

Supplementary Figure 11. ^{1}H NMR spectrum (400 MHz, D_2O, 298K) of compound **8**.

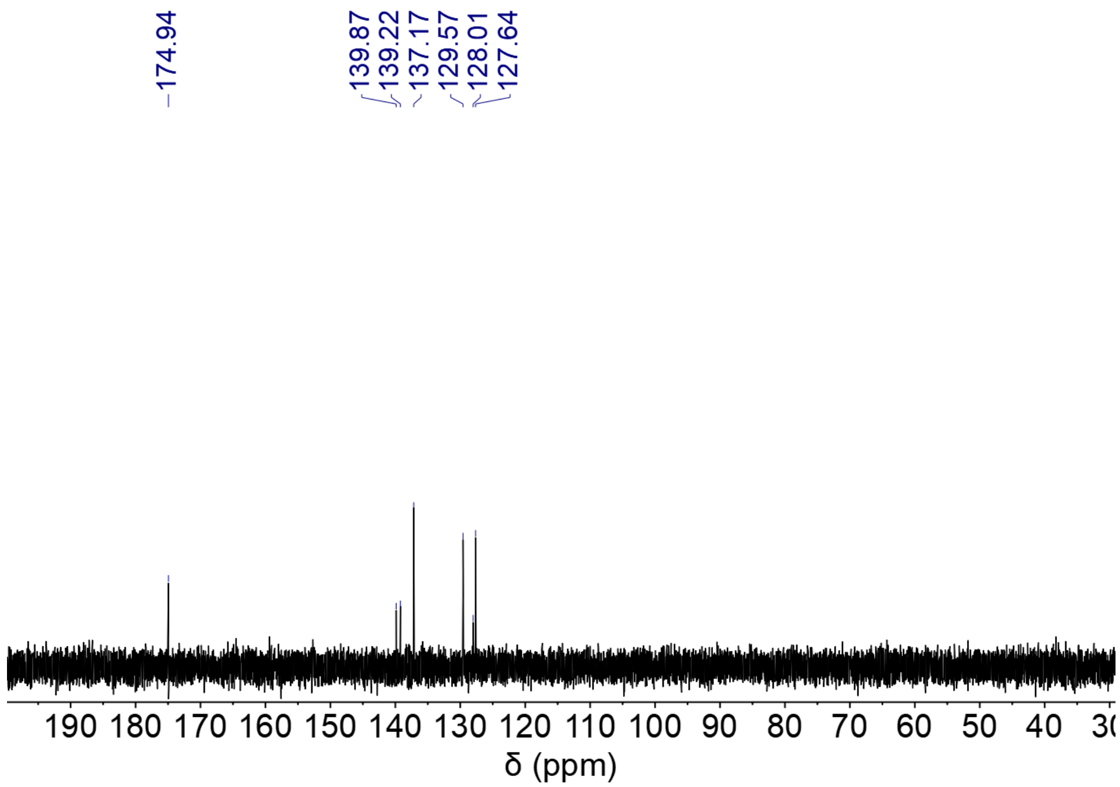

Supplementary Figure 12. ^{13}C NMR spectrum (100 MHz, D_2O, 298K) of compound **8**.

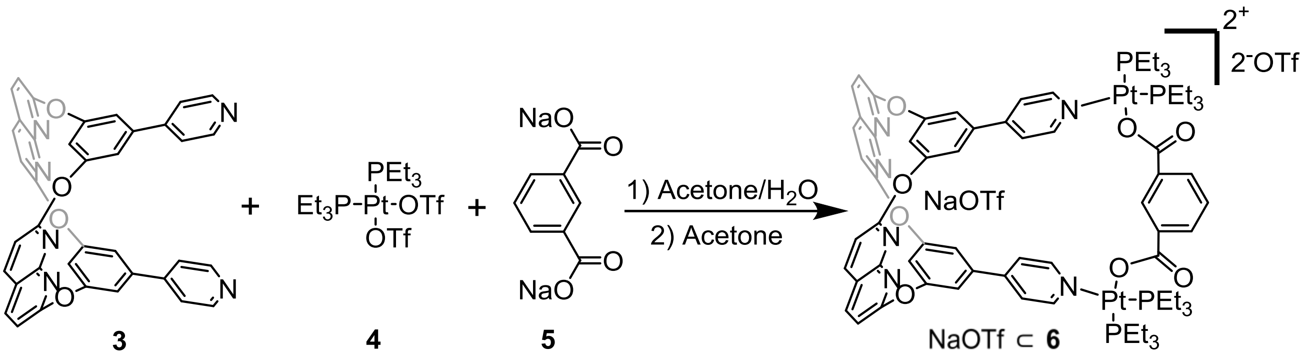

Supplementary Figure 13. The synthesis of NaOTf ⊂ **6**.

Synthesis of NaOTf ⊂ 6. 3 (6.27 mg, 10 μmol), *cis*-$Pt(PEt_3)_2(OTf)_2$ **4** (14.59 mg, 20 μmol) and dicarboxylate ligand **5** (2.10 mg, 10 μmol), were placed in a 2-dram vial, followed by addition of H_2O (0.2 mL) and acetone (1.0 mL). After 3 h of heating at 50 ^{o}C, all solvent was removed by N_2 flow. Acetone (1.0 mL) was then added into the resultant mixture, and the solution was stirred at 50 ^{o}C for 8 h. The resulting product NaOTf ⊂ **6** was precipitated with diethyl ether, isolated and dried under reduced pressure and dissolved in acetone-d_6 for characterization, white solid: 20.39 mg, Yield: 96%. The ^{1}H NMR spectrum of NaOTf ⊂ **6** is shown in Supplementary Figure 14. ^{1}H NMR (400 MHz, acetone-d_6, 298 K) δ (ppm): 9.04 (m, 4H), 8.55 (d, J = 8.7 Hz, 4H), 8.22 (t, J = 1.8 Hz, 1H), 8.06 (d, J = 6.0 Hz, 4H), 7.81 (dd, J = 7.6, 1.7 Hz, 2H), 7.73 (d, J = 2.0 Hz, 4H), 7.36 (d, J = 8.8 Hz, 6H), 7.18 (t, J = 7.7 Hz, 1H), 2.07 – 1.88 (m, 48H), 1.30 (m, 72H). The ^{31}P{^{1}H} NMR spectrum of NaOTf ⊂ **6** is shown in Supplementary Figure 15. ^{31}P{^{1}H} NMR (121 MHz, acetone-d_6, 298 K) δ (ppm): 6.96 (d, $^{2}J_{P-P}$ = 21.5 Hz, ^{195}Pt satellites, $^{1}J_{Pt-P}$ = 3229 Hz), and 1.66 (d, $^{2}J_{P-P}$ = 21.5 Hz, ^{195}Pt satellites, $^{1}J_{Pt-P}$ = 3428 Hz). ESI-TOF-MS is shown in Supplementary Figure 16: 912.23 $[M+NaOTf-2OTf^-]^{2+}$. 2D DOSY NMR spectrum (500 MHz, acetone-d_6, 30 mM, 298 K) of NaOTf ⊂

6 is shown in Supplementary Figure 17, $D = 9.00 \times 10^{-10}$ m^2s^{-1}.

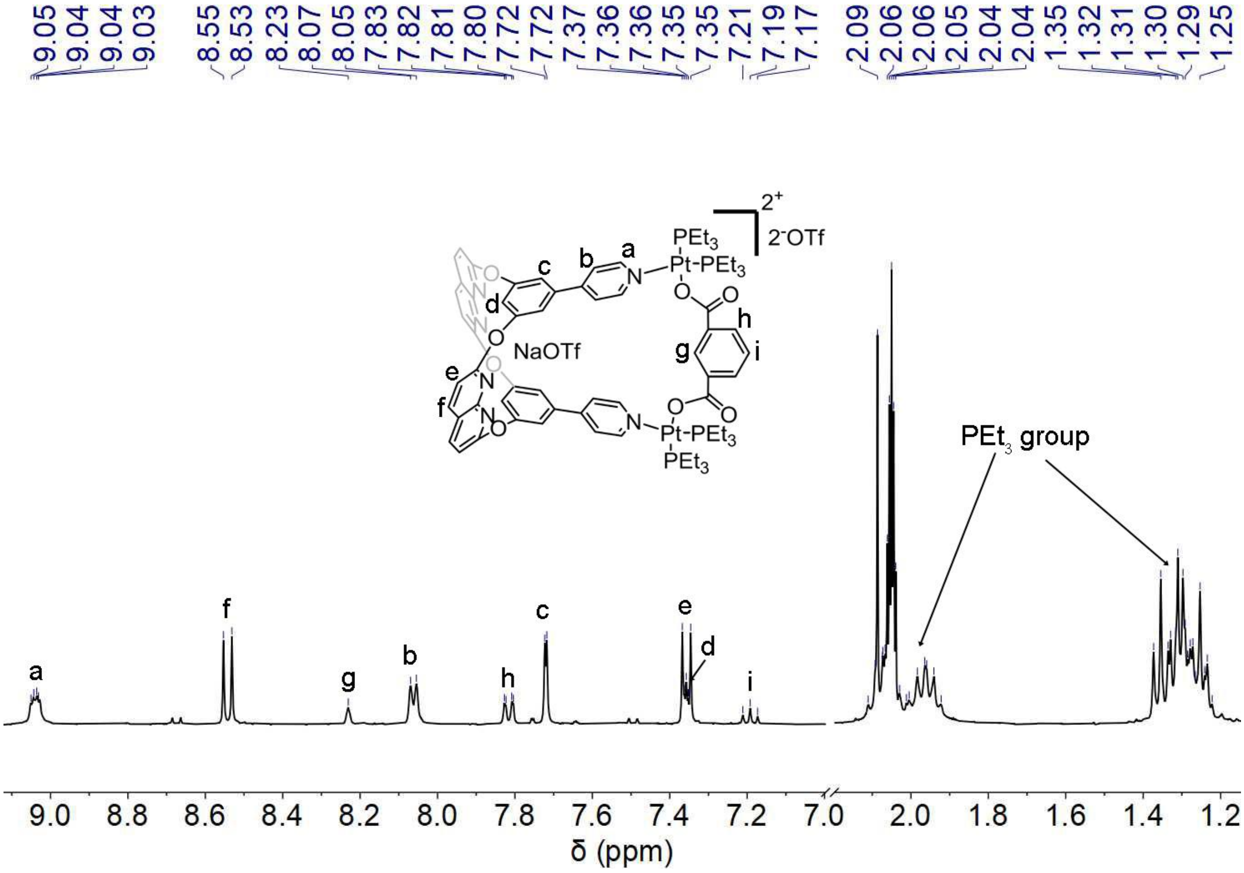

Supplementary Figure 14. ^{1}H NMR spectrum (400 MHz, acetone-d_6, 298K) of NaOTf ⊂ **6**.

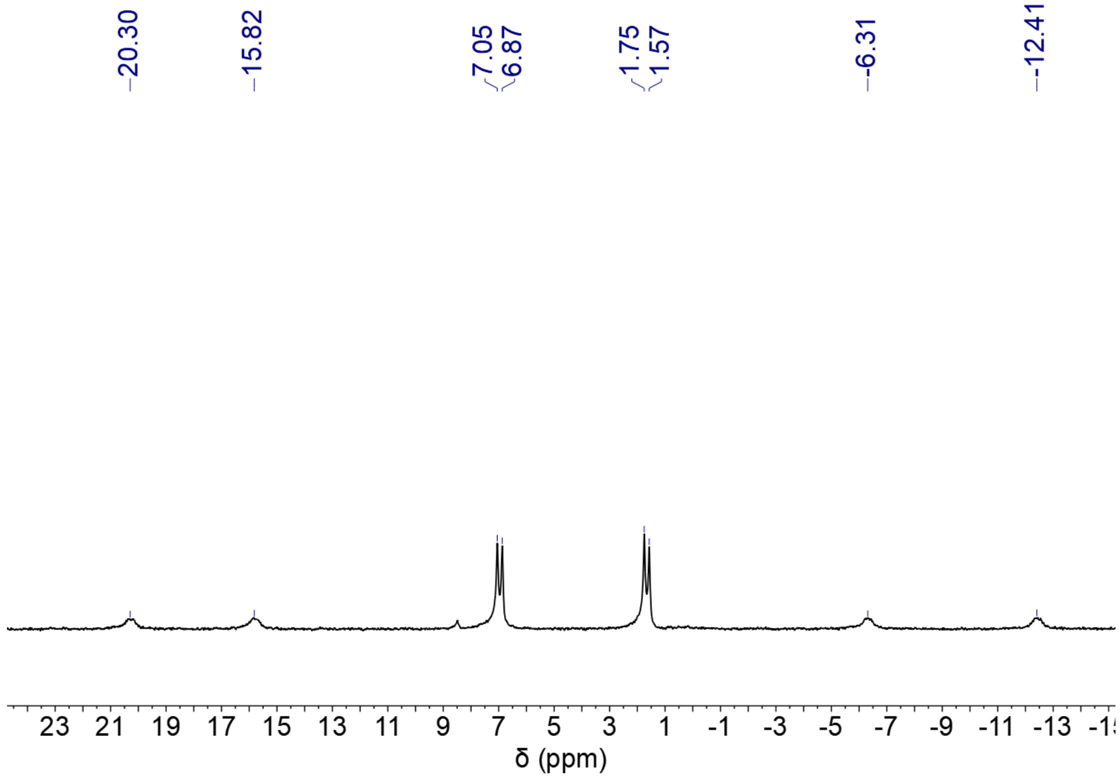

Supplementary Figure 15. ^{31}P{^{1}H} NMR spectrum (121 MHz, acetone-d_6, 298K) of NaOTf ⊂ **6**.

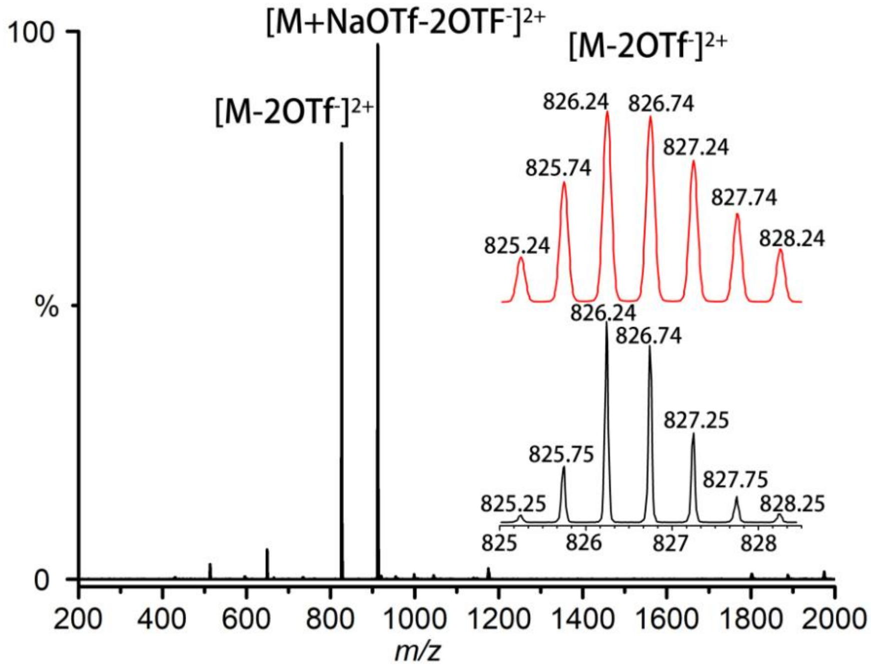

Supplementary Figure 16. Experimental (black) and calculated (red) electrospray ionization mass spectrum of NaOTf ⊂ **6**.

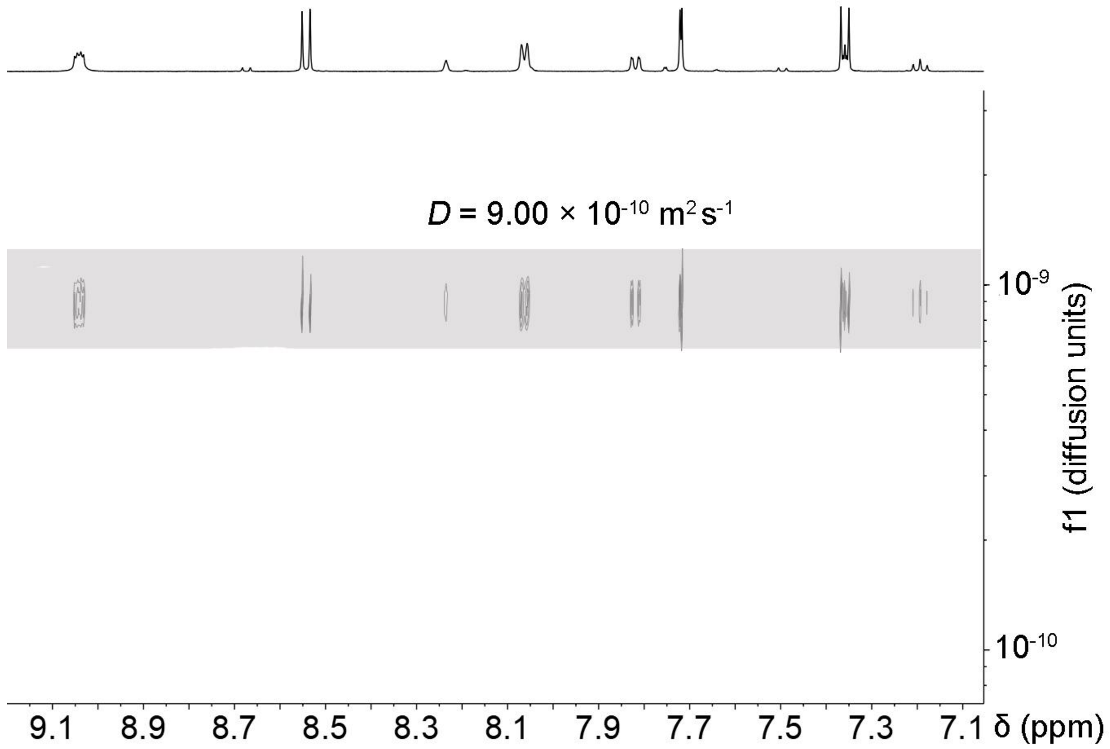

Supplementary Figure 17. DOSY NMR spectra (500 MHz, acetone-d_6, 298 K) of NaOTf ⊂ **6** at 2.0 mM.

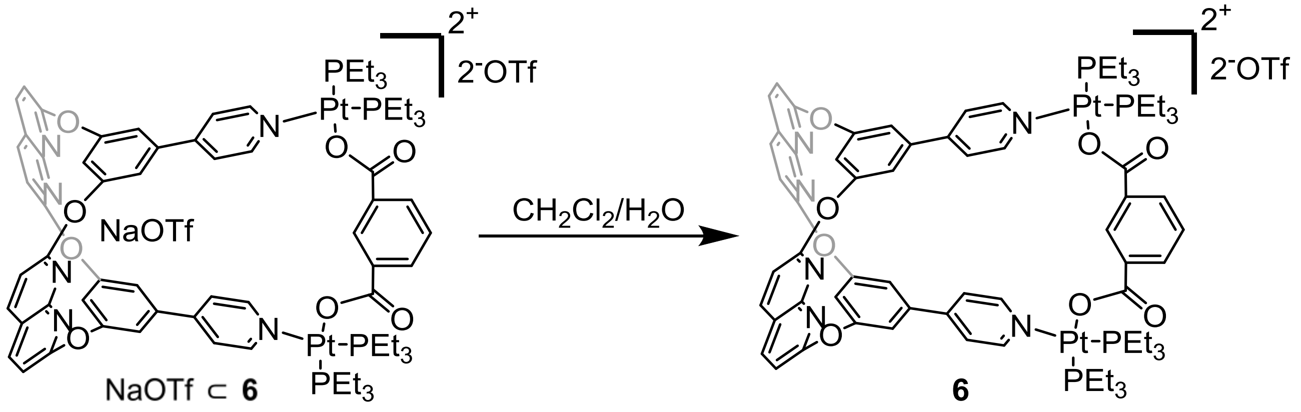

Supplementary Figure 18. The synthesis of **6**.

Synthesis of 6. NaOTf ⊂ **6** (21.24 mg, 10 μmol) was placed in a 2-dram vial, followed by addition of H_2O (2.0 mL) and CH_2Cl_2 (3.0 mL). The mixture was stirred at room temperature for 12 h. Then, organic phase was collected and dried over anhydrous $MgSO_4$, filtered. CH_2Cl_2 was removed by N_2 flow and the solid was dried under vacuum, white solid: 19.40 mg, Yield: 99%. The solid was dissolved in CD_2Cl_2 for characterization. The ^1H NMR spectrum of **6** is shown in Supplementary Figure 19. ^1H NMR (400 MHz, CD_2Cl_2, 298 K) δ (ppm): 8.76 (m, 4H), 8.20 – 8.08 (m, 5H), 7.74 (d, J = 5.9 Hz, 4H), 7.69 (m, 2H), 7.35 (d, J = 2.1 Hz, 4H), 7.14 (d, J = 8.6 Hz, 4H), 7.09 (t, J = 2.1 Hz, 2H), 7.04 (t, J = 7.7 Hz, 1H), 1.98 – 1.65 (m, 48H), 1.29 – 1.19 (m, 72H). The $^{31}P\{^1H\}$ NMR spectrum of **6** is shown in Supplementary Figure 20. $^{31}P\{^1H\}$ NMR (121 MHz, CD_2Cl_2, 298 K) δ (ppm): 5.35 (d, $^2J_{P\text{-}P}$ = 21.4 Hz, ^{195}Pt satellites, $^1J_{Pt\text{-}P}$ = 3216 Hz), and -0.22 (d, $^2J_{P\text{-}P}$ = 21.7 Hz, ^{195}Pt satellites, $^1J_{Pt\text{-}P}$ = 3415 Hz). ESI-TOF-MS is shown in Supplementary Figure 21: m/z = 826.24 $[M-2OTf]^{2+}$. 2D DOSY NMR spectrum (500 MHz, CD_2Cl_2, 30 Mm, 298 K) of **6** is shown in Supplementary Figure 22, $D = 6.47 \times 10^{-10}$ m^2s^{-1}.

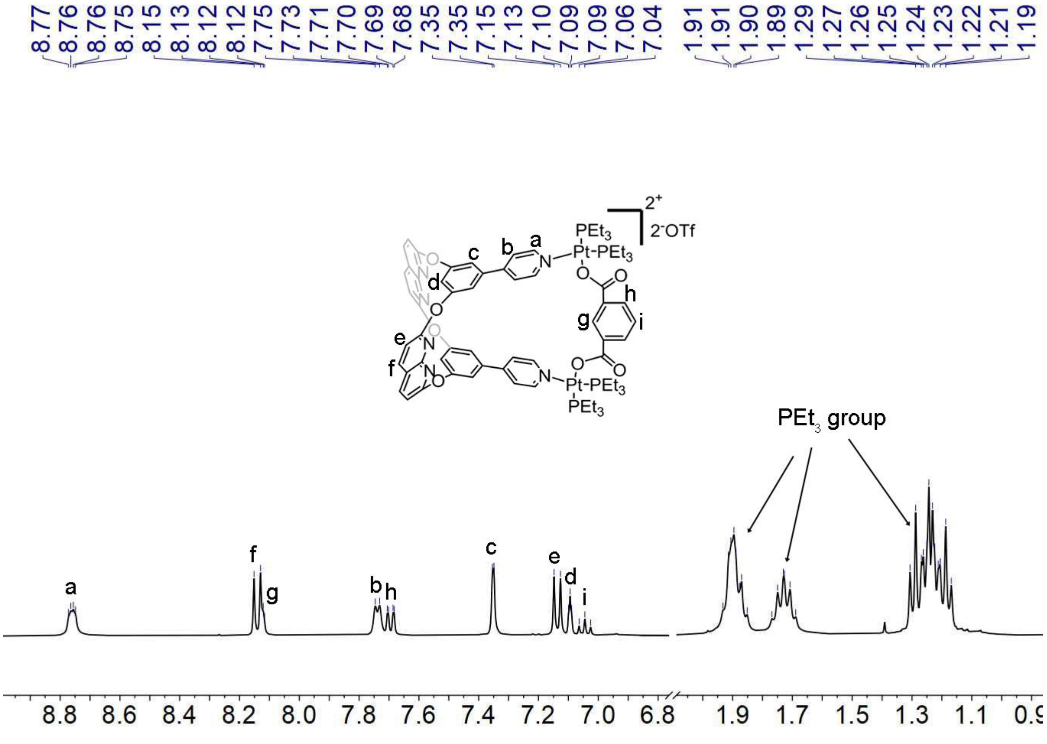

Supplementary Figure 19. ^1H NMR spectrum (400 MHz, CD_2Cl_2, 298K) of **6**.

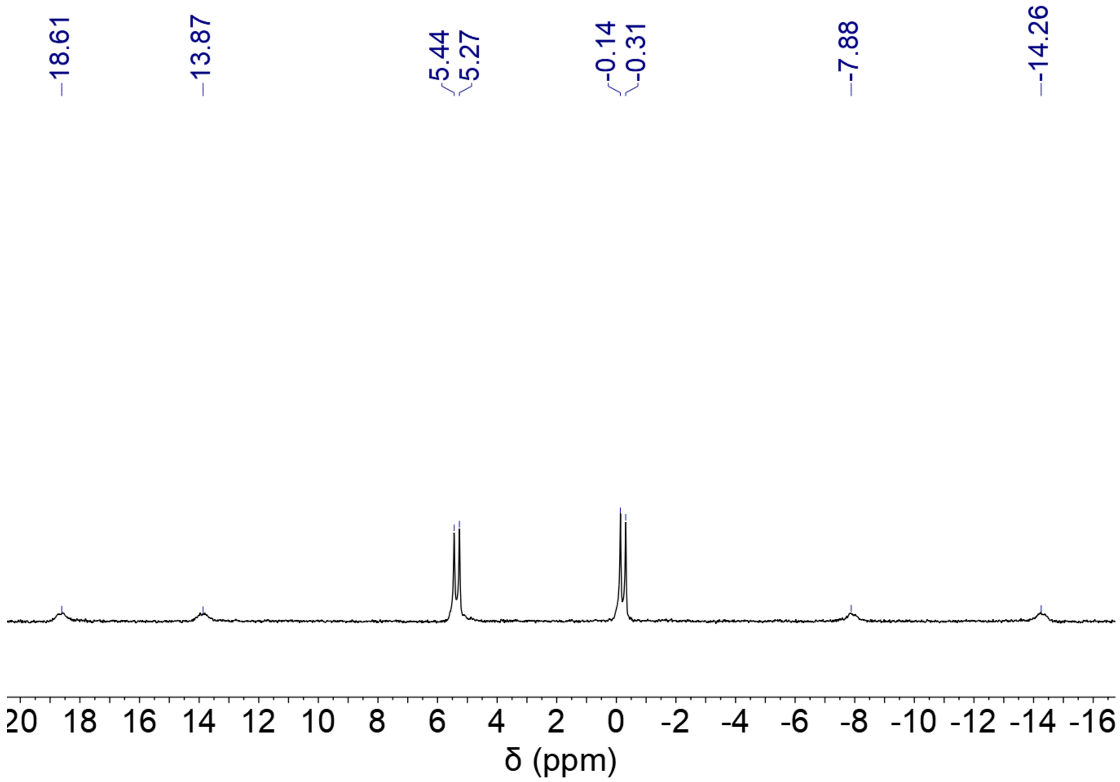

Supplementary Figure 20. $^{31}P\{^{1}H\}$ NMR spectrum (121 MHz, CD_2Cl_2, 298K) of **6**.

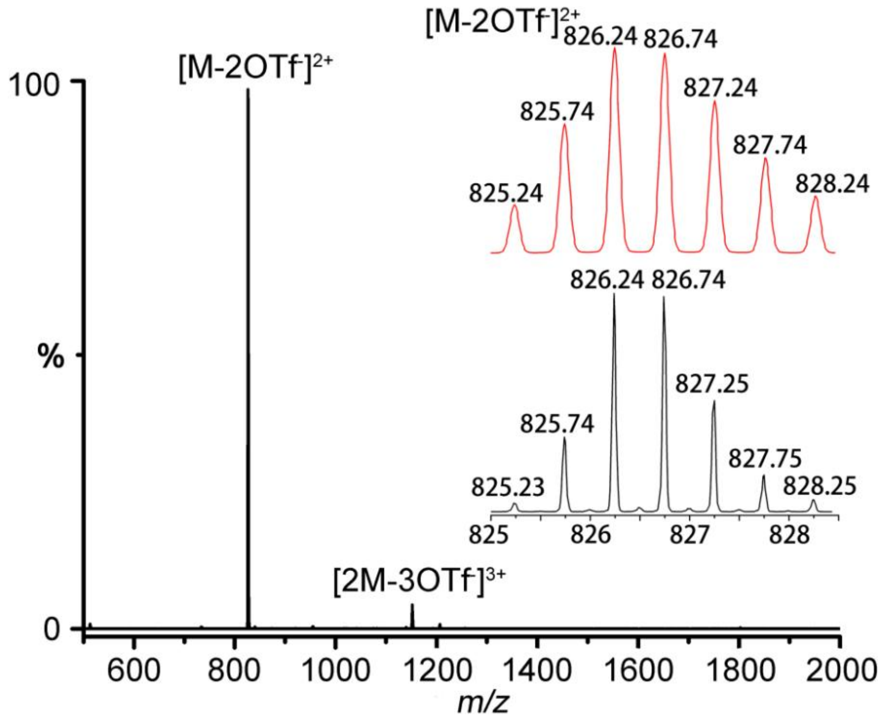

Supplementary Figure 21. Experimental (black) and calculated (red) electrospray ionization mass spectrum of **6**/**7**. $[M\text{-}2OTf^-]^{2+}$ represents the ESI-TOF-MS of monomeric cage **6**. For the experimental and calculated ESI-TOF-MS data of $[2M\text{-}3OTf^-]^{3+}$ (corresponding to catenane **7**), please see Figure 3b in the manuscript.

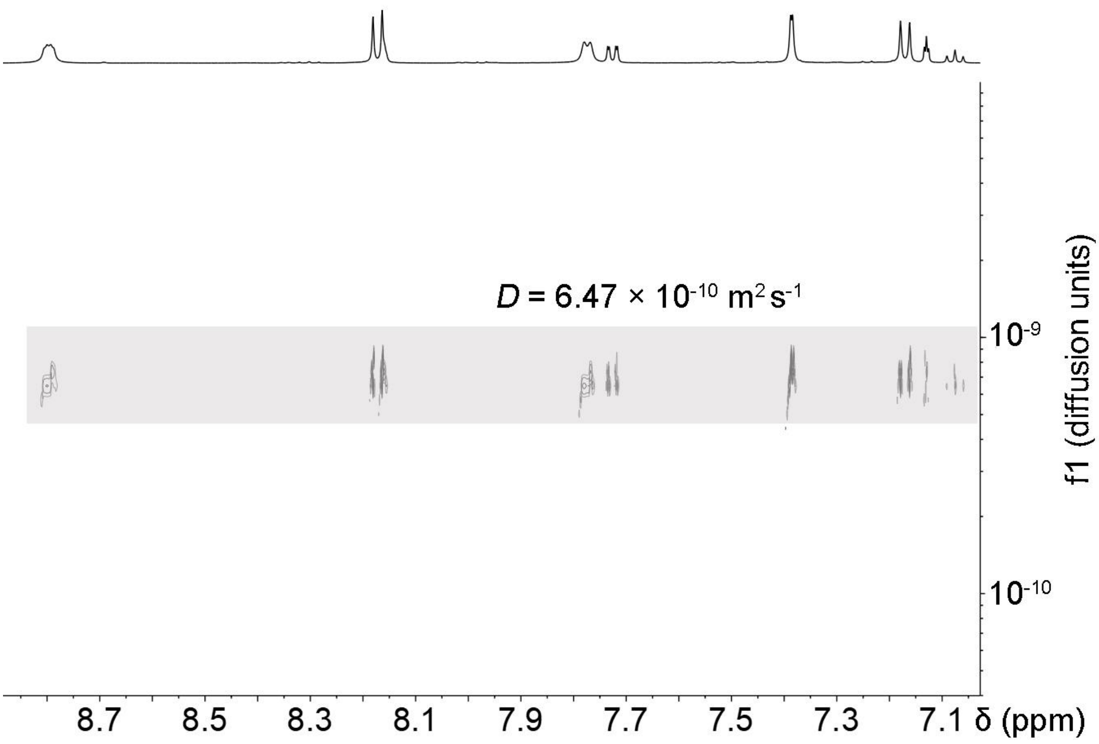

Supplementary Figure 22. DOSY NMR spectra (500 MHz, CD_2Cl_2, 298 K) of **6** at 2.0 mM.

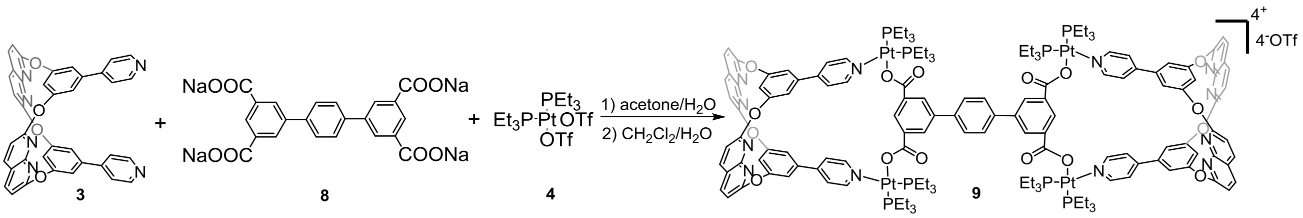

Supplementary Figure 23. The synthesis of **9**.

Synthesis of 9. 3 (6.27 mg, 10 μmol), *cis*-$Pt(PEt_3)_2(OTf)_2$ **4** (14.59 mg, 20 μmol) and tetracarboxylate ligand **8** (2.47 mg, 5 μmol), were placed in a 2-dram vial, followed by addition of H_2O (0.2 mL) and acetone (1.0 mL). After 3 h of heating at 50 ^{o}C, all solvent was removed by N_2 flow. Acetone (1.0 mL) was then added into the resultant mixture, and the solution was stirred at 50 ^{o}C for 8 h. The resulting product was precipitated with diethyl ether, isolated and dried under reduced pressure and dissolved in CH_2Cl_2 (3.0 mL). Above solution was placed in a 2-dram vial, followed by addition of H_2O (2.0 mL), and stirred at room temperature for 12 h. Organic phase was collected and dried over anhydrous $MgSO_4$, filtered. CH_2Cl_2 was removed by N_2 flow and the solid was dried under vacuum, white solid: 18.27 mg, Yield: 92%. The solid was dissolved in CD_2Cl_2 for characterization. The ^{1}H NMR spectrum of **9** is shown in Supplementary Figure 24. ^{1}H NMR (400 MHz, CD_2Cl_2, 298 K) δ (ppm): 8.98 – 8.65 (8H), 8.27 – 8.07 (6H), 7.93 (s, 6H), 7.85 – 7.66 (8H), 7.50 – 7.28 (8H), 7.26 (s, 6H), 7.19 – 6.89 (12H), 2.05 – 1.67 (m, 96H), 1.41 – 1.07 (m, 144H). The ^{31}P{^{1}H} NMR spectrum of **9** is shown in Supplementary Figure 25. ^{31}P{^{1}H} NMR (121 MHz, CD_2Cl_2, 298 K) δ (ppm): 5.48 (d, $^{2}J_{P-P}$ = 21.8 Hz, ^{195}Pt satellites, $^{1}J_{Pt-P}$ = 3250 Hz), and -0.10 (d, $^{2}J_{P-P}$ = 21.6 Hz, ^{195}Pt satellites, $^{1}J_{Pt-P}$ = 3461 Hz). ESI-TOF-MS is shown in Supplementary Figure 26: 845.13 $[M - 4OTf^{-}]^{4+}$. 2D DOSY NMR spectrum (500 MHz, CD_2Cl_2, 15 mM, 298 K) of **9** is shown in Supplementary Figure 27, $D = 3.91 \times 10^{-10}$ m^2s^{-1}.

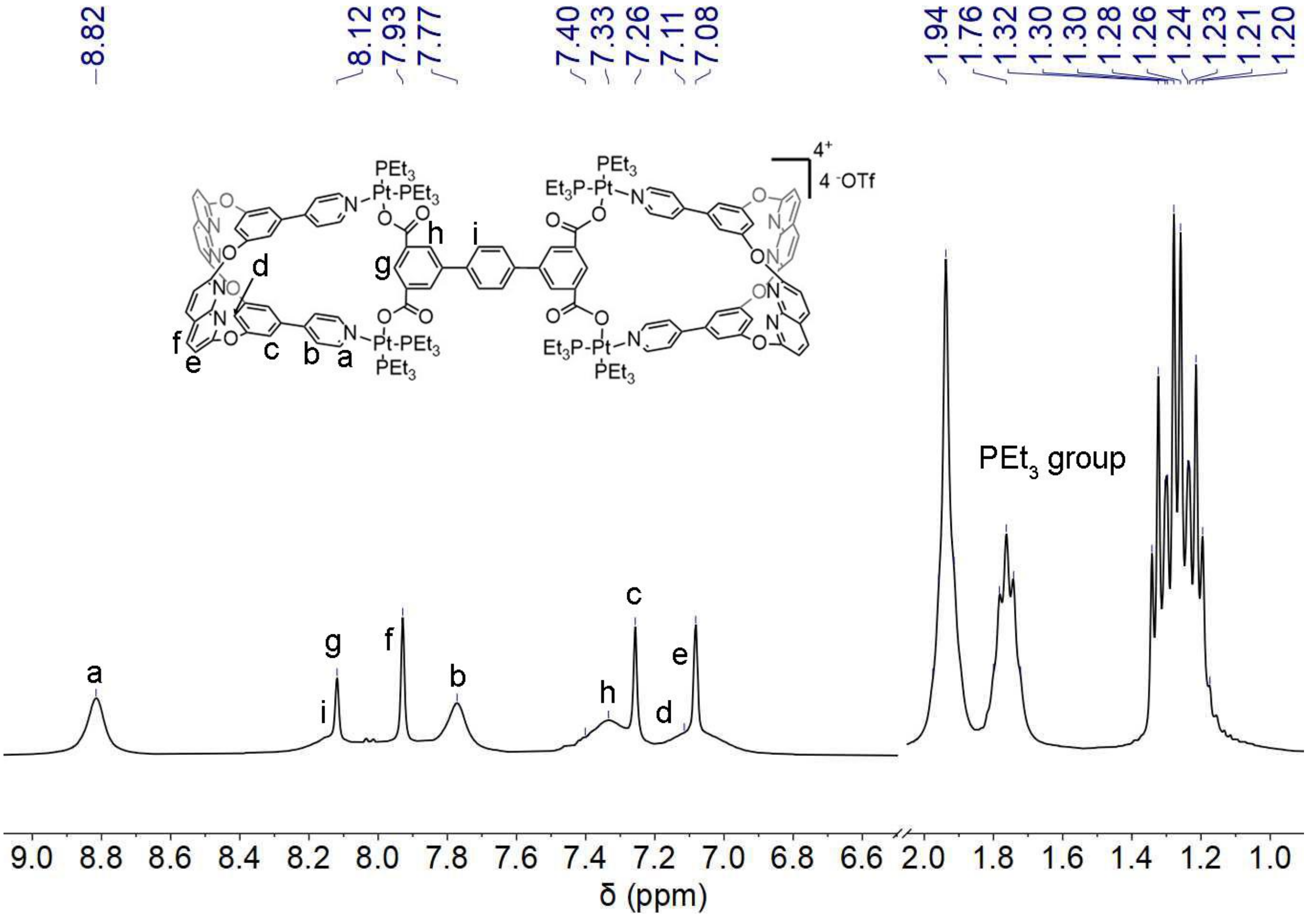

Supplementary Figure 24. ^{1}H NMR spectrum (400 MHz, CD_2Cl_2, 298K) of **9**.

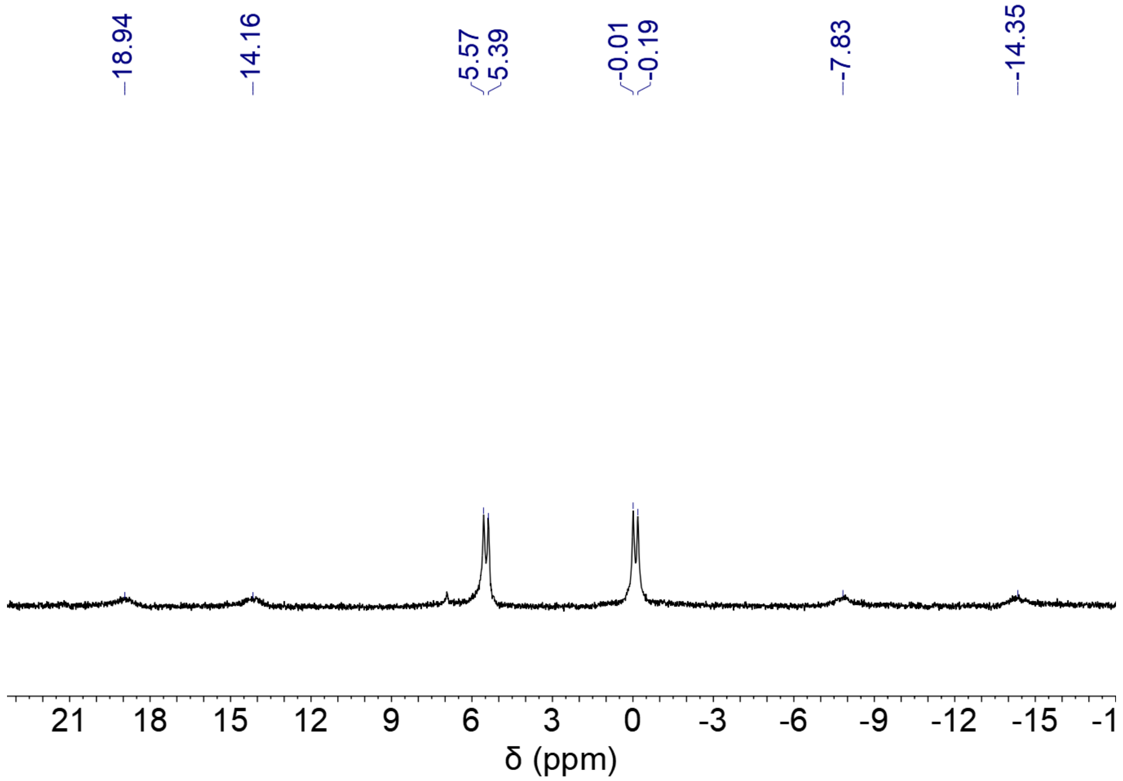

Supplementary Figure 25. $^{31}P\{^{1}H\}$ NMR spectrum (121 MHz, CD_2Cl_2, 298K) of **9**.

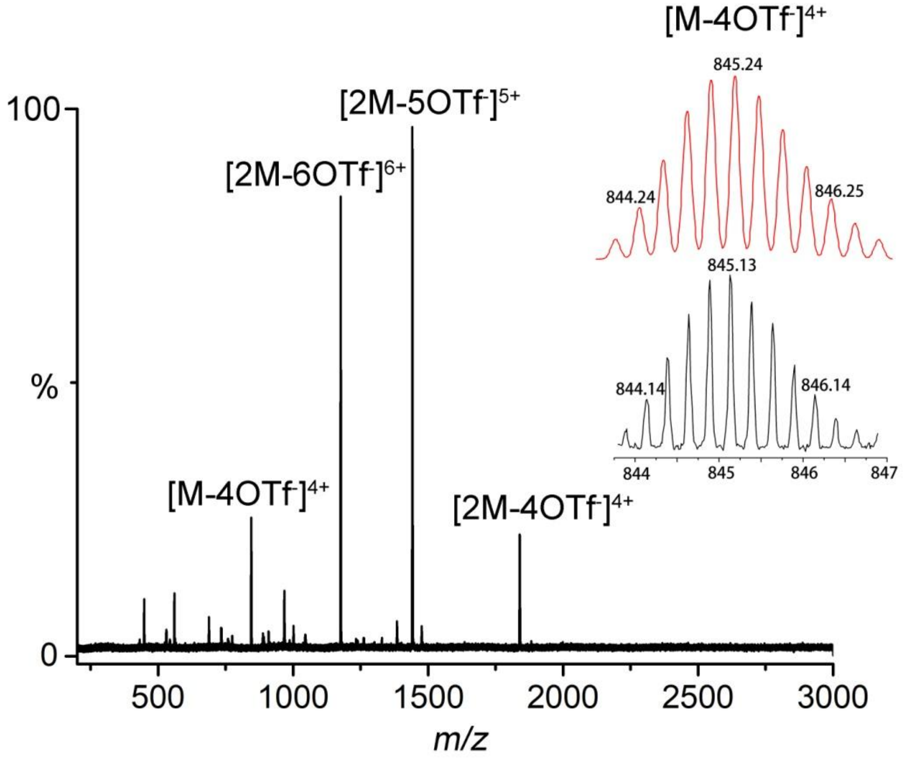

Supplementary Figure 26. Experimental (black) and calculated (red) electrospray ionization mass spectrum of **9**/**10**. $[M\text{-}4OTf^{-}]^{4+}$ represents the ESI-TOF-MS of bis-metallacage **9**. For the experimental and calculated ESI-TOF-MS data of $[2M\text{-}6OTf^{-}]^{6+}$, $[2M\text{-}5OTf^{-}]^{5+}$, and $[2M\text{-}4OTf^{-}]^{4+}$ (corresponding to cyclic bis[2]catenane **10**), please see Figure 3c in the manuscript.

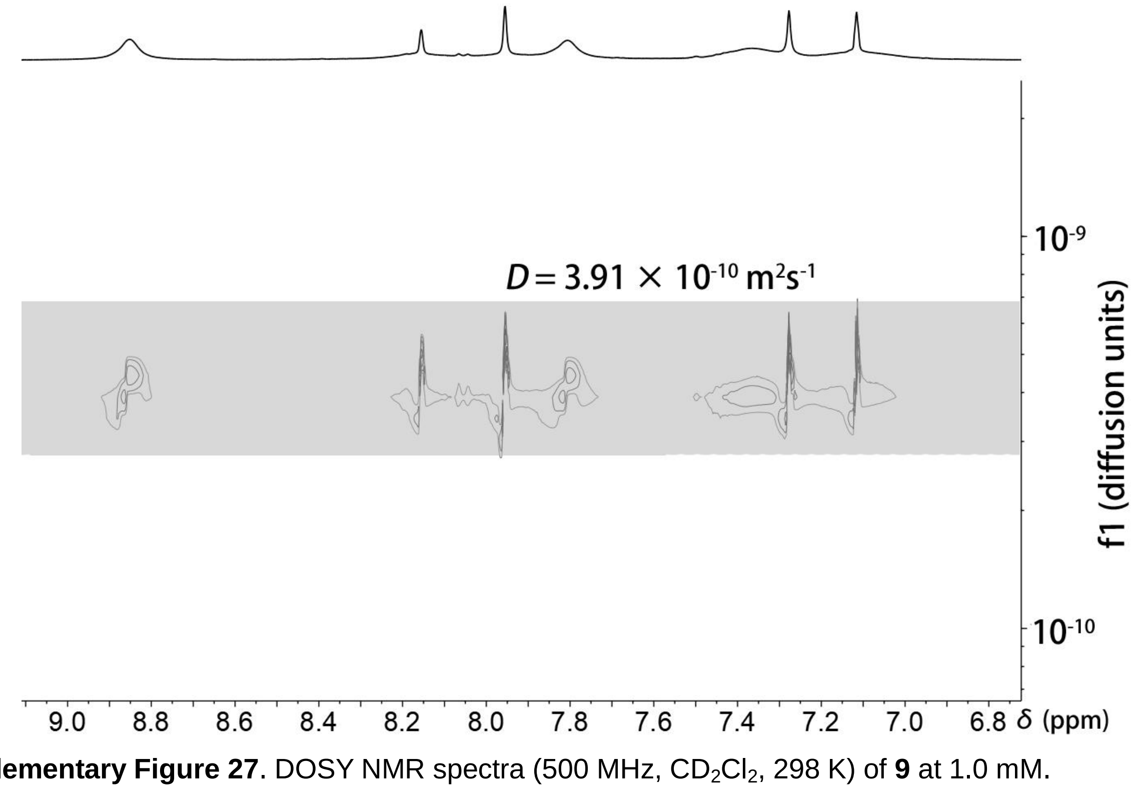

Supplementary Figure 27. DOSY NMR spectra (500 MHz, CD_2Cl_2, 298 K) of **9** at 1.0 mM.

Supplementary Table 1. Crystal data and structure refinement for **3**

Empirical formula	$C_{40}H_{26}Cl_4N_6O_4$
Formula weight	796.47
Temperature	173(2) K
Wavelength	0.71073 Å
Crystal system	Orthorhombic
Space group	Cmca
Unit cell dimensions	*a* = 20.2564(11) Å *α* = 90°.
	b = 19.0787(11) Å *β* = 90°.
	c = 19.8420(11) Å *γ* = 90°.
Volume	7668.3(7) $Å^3$
Z	8
Density (calculated)	1.380 Mg/m^3
Absorption coefficient	0.358 mm^{-1}
F(000)	3264
Crystal size	0.220 x 0.190 x 0.160 mm^3
Theta range for data collection	2.369 to 25.008°.
Index ranges	-24<=h<=24, -16<=k<=22, -23<=l<=22
Reflections collected	22404
Independent reflections	3480 [R(int) = 0.0341]
Refinement method	Full-matrix least-squares on F^2
Data / restraints / parameters	3480 / 0 / 256
Goodness-of-fit on F^2	1.035
Final *R* indices [*I*>2sigma(*I*)]	R_1 = 0.0467, wR_2 = 0.1294
R indices (all data)	R_1 = 0.0555, wR_2 = 0.1369
Extinction coefficient	n/a
Largest diff. peak and hole	0.923 and -0.574 e.$Å^{-3}$
CCDC	1939272

Crystal Structure Determination and refinements (For **3**, NaOTf ⊂ **6** and **7**)**.** Single-crystal X-ray diffraction data was collected on a Nonius KappaCCD diffractometer equipped with Mo K-alpha radiation (λ = 0.71073 Å) and a BRUKER APEXII CCD. Throughout data collection, the crystal was cooled with an Oxford Cryosystem. The APEX3 software suite was used to manage data collection, reduction (SAINT V8.38A1), and absorption correction by the Multi-scan method (SADABS), structure determination via direct methods (SHLEXT) and model refinement (SHELXL). All non-hydrogen atoms were refined anisotropically though many atoms required anisotropic displacement parameter restraints. All hydrogen atoms were refined with isotropic displacement coefficients and their positions ideally constrained.

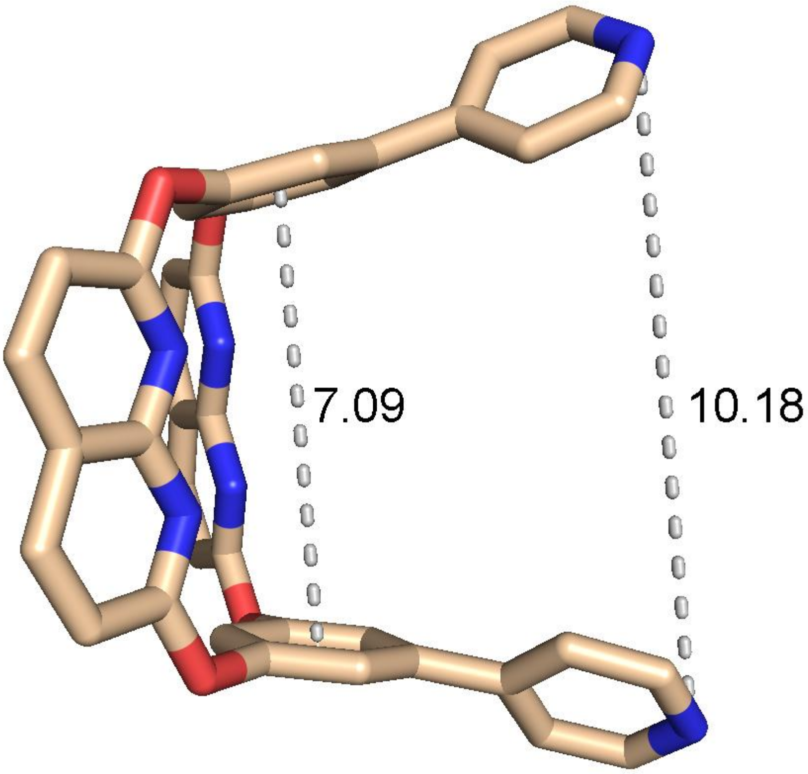

Supplementary Figure 28. Centroid–centroid distance and N···N distance (Å) of two benzene-pyridine arms from tweezer **3** in solid state (hydrogen atoms are omitted).

Supplementary Table 2. Crystal data and structure refinement for NaOTf ⊂ **6**

Empirical formula	$C_{88}H_{116}F_9N_6NaO_{22}P_4Pt_2S_3$
Formula weight	2414.09
Temperature	103(2) K
Wavelength	0.71073 Å
Crystal system	monoclinic
Space group	P 1 21/n 1
Unit cell dimensions	a = 19.2590(5) Å α = 90°.
	b = 20.6064(5) Å β = 100.6880(10)°.
	c = 26.1521(6) Å γ = 90°.
Volume	10198.6(4) Å^3
Z	4
Density (calculated)	1.572 Mg/m^3
Absorption coefficient	2.955 mm^{-1}
F(000)	4872
Crystal size	0.060 x 0.145 x 0.165 mm^3
Theta range for data collection	2.13 to 31.01°.
Index ranges	-27<=h<=27, -29<=k<=29, -37<=l<=34
Reflections collected	161925
Independent reflections	32493 [R(int) = 0.0735]
Refinement method	Full-matrix least-squares on F^2
Data / restraints / parameters	32493 / 1579 / 1460
Goodness-of-fit on F^2	1.014
Final R indices [I>2sigma(I)]	R_1 = 0.00365, wR_2 = 0.0656
R indices (all data)	R_1 = 0.0670, wR_2 = 0.0735
Extinction coefficient	n/a
Largest diff. peak and hole	1.182 and -0.923 e.Å^{-3}
CCDC	1939276

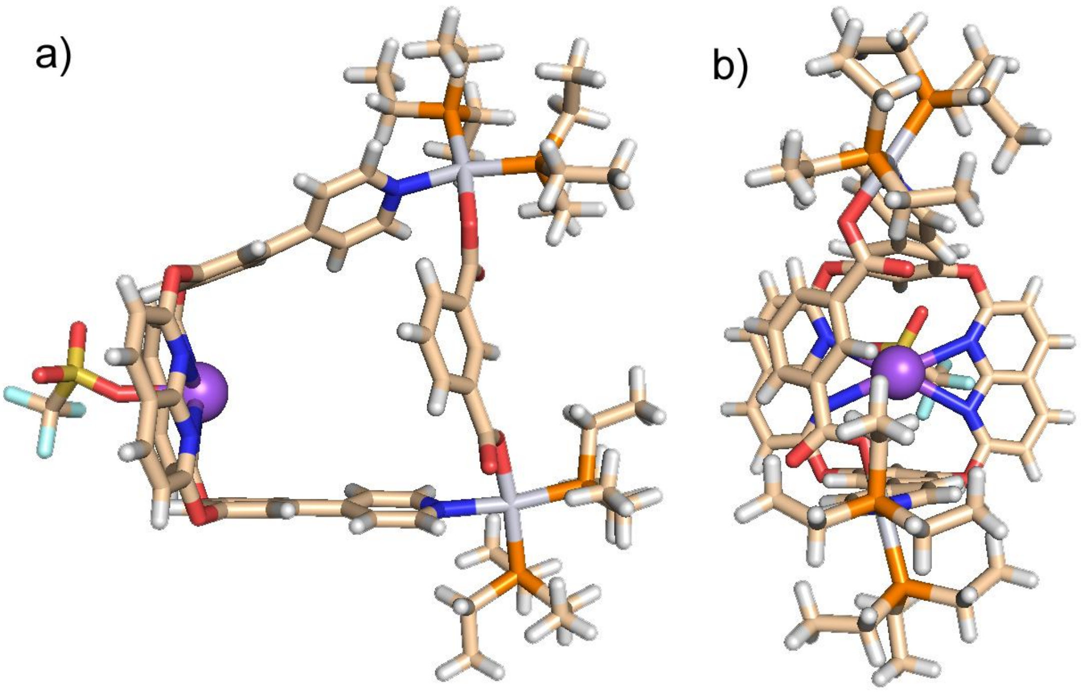

Supplementary Figure 29. Solid state structures of NaOTf ⊂ **6**: (a) side view and (b) front view (acetone and some of OTf^{-} are omitted).

Supplementary Table 3. Crystal data and structure refinement for **7**

Empirical formula	$C_{81}H_{104}F_6N_6O_{17}P_4Pt_2S_2$
Formula weight	2125.88
Temperature	103(2) K
Wavelength	0.71073 Å
Crystal system	monoclinic
Space group	C 1 2/c 1
Unit cell dimensions	*a* = 36.6185(19) Å α = 90°.
	b = 16.1960(9) Å β = 119.833(2)°.
	c = 35.828(3) Å γ = 90°.
Volume	18433.0(2) Å^3
Z	8
Density (calculated)	1.532 Mg/m^3
Absorption coefficient	3.224 mm^{-1}
F(000)	8560
Crystal size	0.135 x 0.195 x 0.400 mm^3
Theta range for data collection	1.42 to 33.14°.
Index ranges	-54<=h<=56, -24<=k<=24, -54<=l<=55
Reflections collected	161477
Independent reflections	35120 [R(int) = 0.0593]
Refinement method	Full-matrix least-squares on F^2
Data / restraints / parameters	35120 / 86 / 1078
Goodness-of-fit on F^2	1.232
Final *R* indices [*I*>2sigma(*I*)]	R_1 = 0.0617, wR_2 = 0.1141
R indices (all data)	R_1 = 0.0839, wR_2 = 0.1198
Extinction coefficient	n/a
Largest diff. peak and hole	2.447 and -3.097 e.Å^{-3}
CCDC	1946245

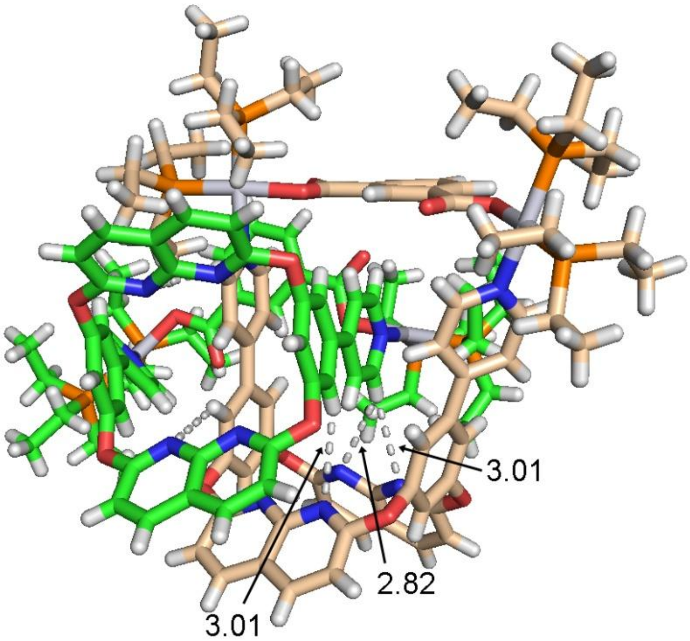

Supplementary Figure 30. Multiple C-H···N hydrogen bonds between H atoms from aromatic rings and N atoms from naphthyridyl groups of **7** in solid state: C-H···N distances (Å).

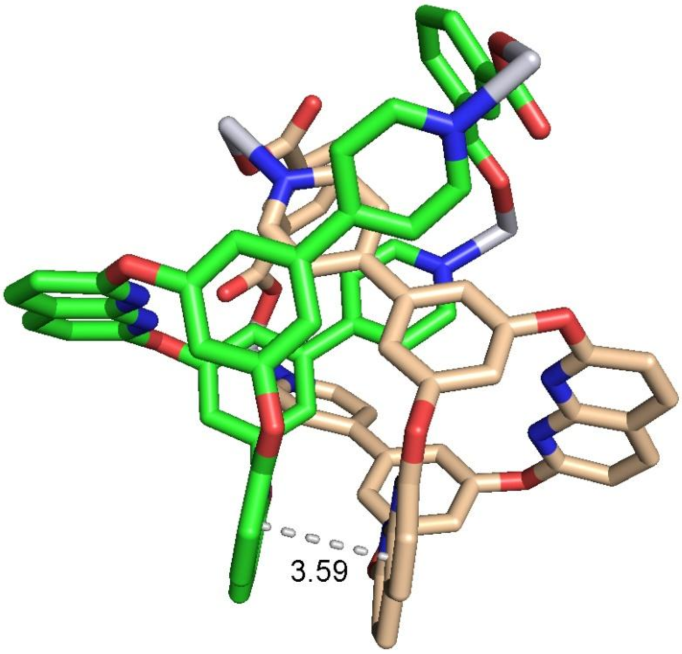

Supplementary Figure 31. π···π stacking between two of four naphthyridine rings of **7** in solid state: centroid···centroid distance (Å) (hydrogen atoms and PEt_3 groups are omitted).

Supplementary Table 4. Containing the Check cif alerts obtained from the checkcif.iucr.org webpage and the authors responses

Check cif alerts	Response
The value of sine(theta_max)/wavelength is less than 0.550	The diffraction limit was found to be 1.5 Å and the data was trimmed accordingly
Non-Solvent Resd Ueq(max)/Ueq(min) Range	Disordered moieties remained unmodelled due to the low data/parameter ratio.
MainMol' Ueq as Compared to neighbours	Disordered moieties remained unmodelled due to the low data/parameter ratio.

Supplementary Table 5. Crystal data and structure refinement for **10**

Empirical formula	$C_{147}F_3N_{12}O_{19}P_8Pt_4S$
Formula weight	3354.73
Temperature	103.15 K
Wavelength	0.71073 Å
Crystal system	monoclinic
Space group	P 1 21/c 1
Unit cell dimensions	*a* = 23.2555(8) Å α = 90°.
	b = 55.5910(19) Å β = 94.909(3)°.
	c = 29.0761(10) Å γ = 90°.
Volume	37452(2) $Å^3$
Z	8
Density (calculated)	1.190 Mg/m^3
Absorption coefficient	3.112 mm^{-1}
F(000)	12744
Crystal size	0.50 x 0.10 x 0.05 mm^3
Theta range for data collection	1.015 to 13.342°.
Index ranges	-15<=h<=15, -36<=k<=36, -18<=l<=18
Reflections collected	106205
Independent reflections	10717 [R(int) = 0.1762]
Refinement method	Full-matrix least-squares on F^2
Data / restraints / parameters	10717 / 1472 / 1089
Goodness-of-fit on F^2	1.031
Final *R* indices [*I*>2sigma(*I*)]	R_1 = 0.0947, wR_2 = 0.2340
R indices (all data)	R_1 = 0.1232, wR_2 = 0.2633
Extinction coefficient	0.000112(15)
Largest diff. peak and hole	3.239 and -1.457 e.$Å^{-3}$
CCDC	1947413

Crystal Structure Determination and refinements (for **10**). Single crystals of **10** were obtained by slow diffusion of *n*-hexane into an acetone solution of **10**. Single-crystal X-ray diffraction data was collected on a Nonius KappaCCD diffractometer equipped with Mo K-alpha radiation (λ = 0.71073 Å) and a BRUKER APEXII CCD. Throughout data collection, the crystal was cooled with an Oxford Cryosystem. The crystal structure was solved and refined against all F^2 values using the SHELX and Olex 2 suite of

programmes[5,6]. Crystals of bis[2]catenane presented a diffraction resolution of 1.5 Å. Only the platinium atoms were refined anisotropically. The rest of the atoms were refined isotropically due to a poor reflexions/parameters ratio. Hydrogen atoms were not placed in the calculated positions. The phenyl, napthyridyl and pyridyl groups were restrained to have idealized geometries using SHELX AFIX commands. The C-O distances in the carboxylate groups, P-C distances and C-C distances in PEt_3 groups were restrained using SHELX DFIX commands. The atomic displacement parameters (adp) of the ligands have been restrained using SHELX DELU, SIMU and REGU commands.

Single crystal of bis[2]catenane presents large voids filled with a lot of scattered electron density. Solvent mask protocol inside Olex 2 software was used to account for the void electron density corresponding to the disordered OTf^- anions and solvent molecules placed in the intermolecular space in the crystal structure.

A large number of A-alerts and B-alerts were found due to poor resolution (1.5 Å). Rigid Body was used to solve the structure of the bis[2]catenane. Different moieties of the bis[2]catenane were restrained to have idealized geometries and allowed to refine their position and orientation.

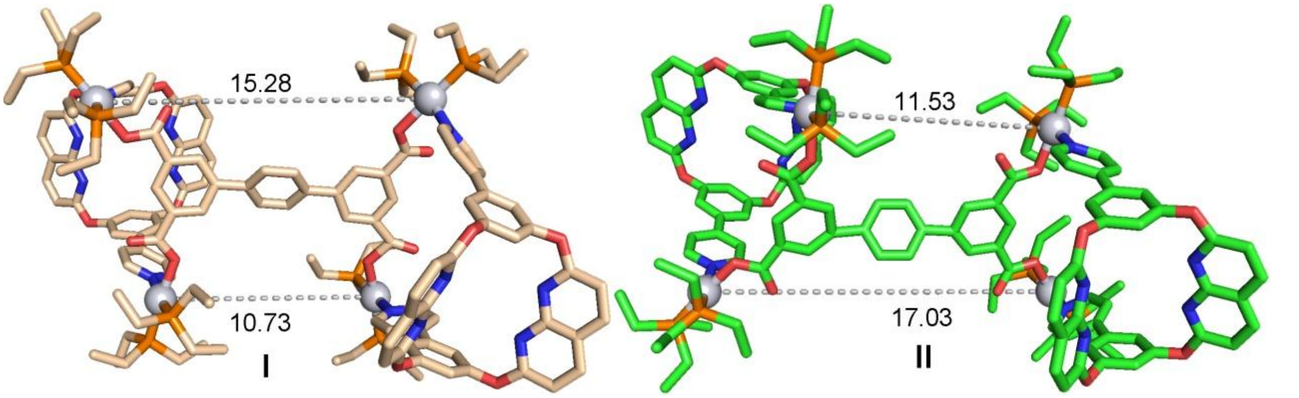

Supplementary Figure 32. Two conformations of bis-metallacage **9** (**I** & **II**) in solid state: Pt···Pt distances (Å).

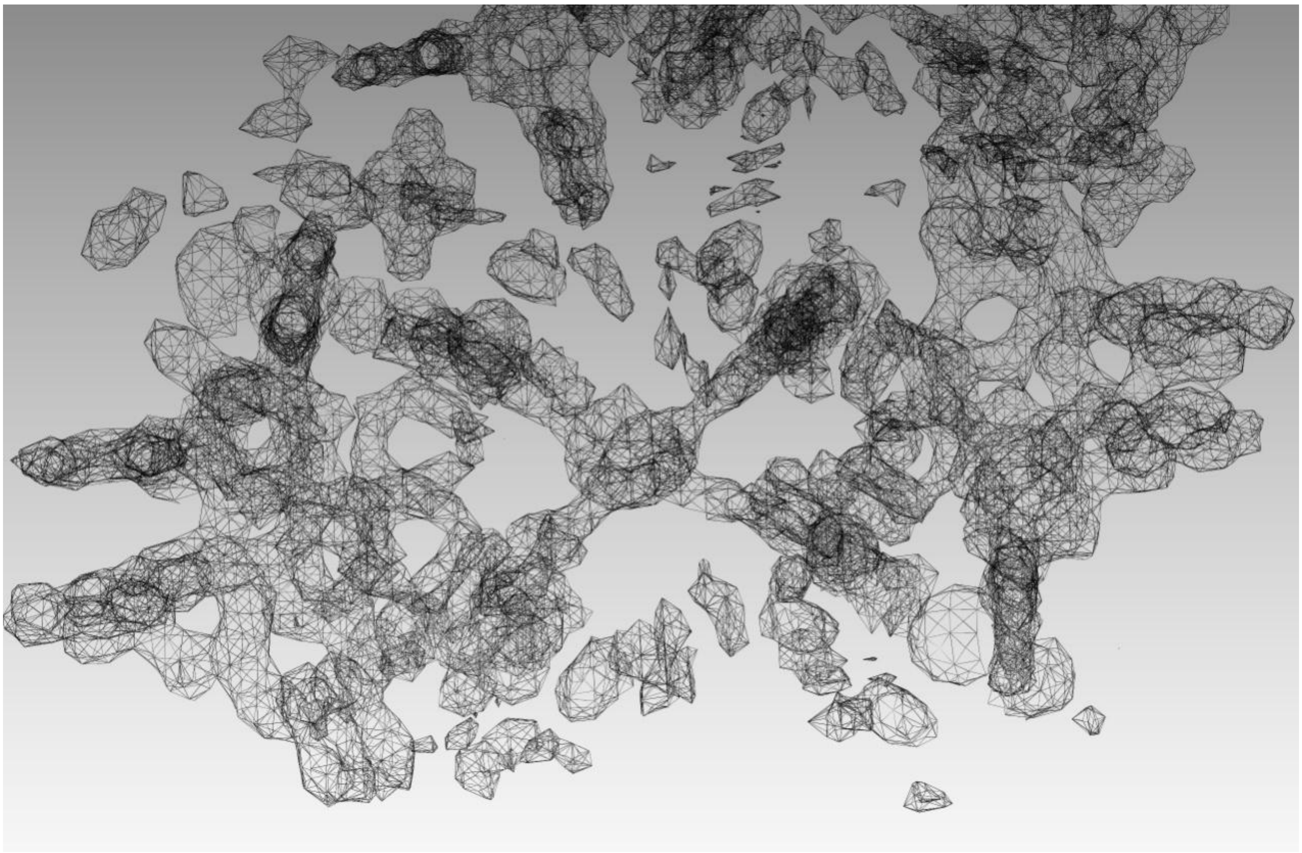

Supplementary Figure 33. $2F_o$-F_c electronic density map (1.50 electrons per $Å^3$) of **10**.

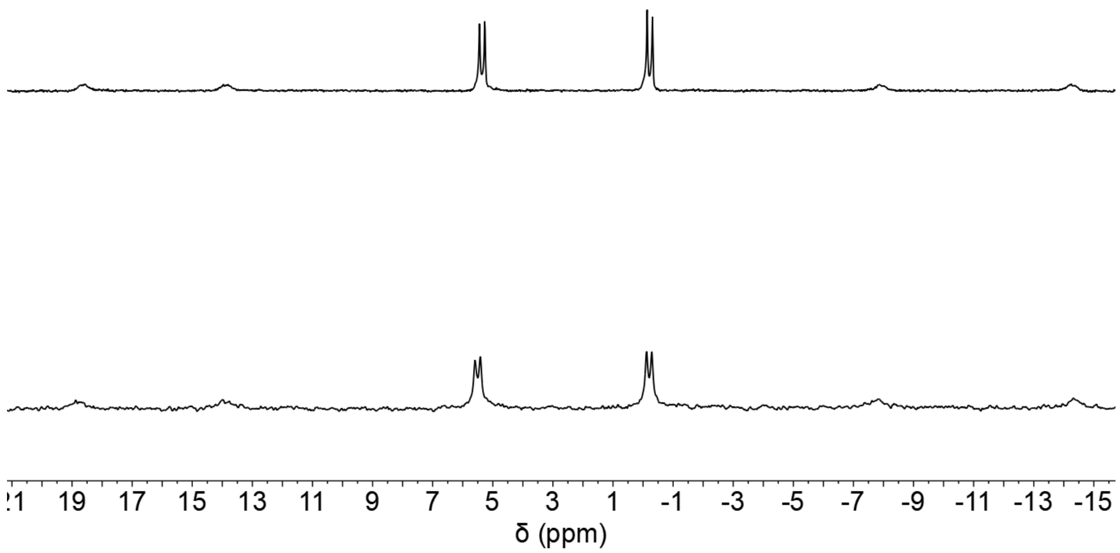

Supplementary Figure 34. $^{31}P\{^{1}H\}$ NMR spectra (121 MHz, CD_2Cl_2, 298K) of NaOTf ⊂ **6** (bottom) and **6** (up).

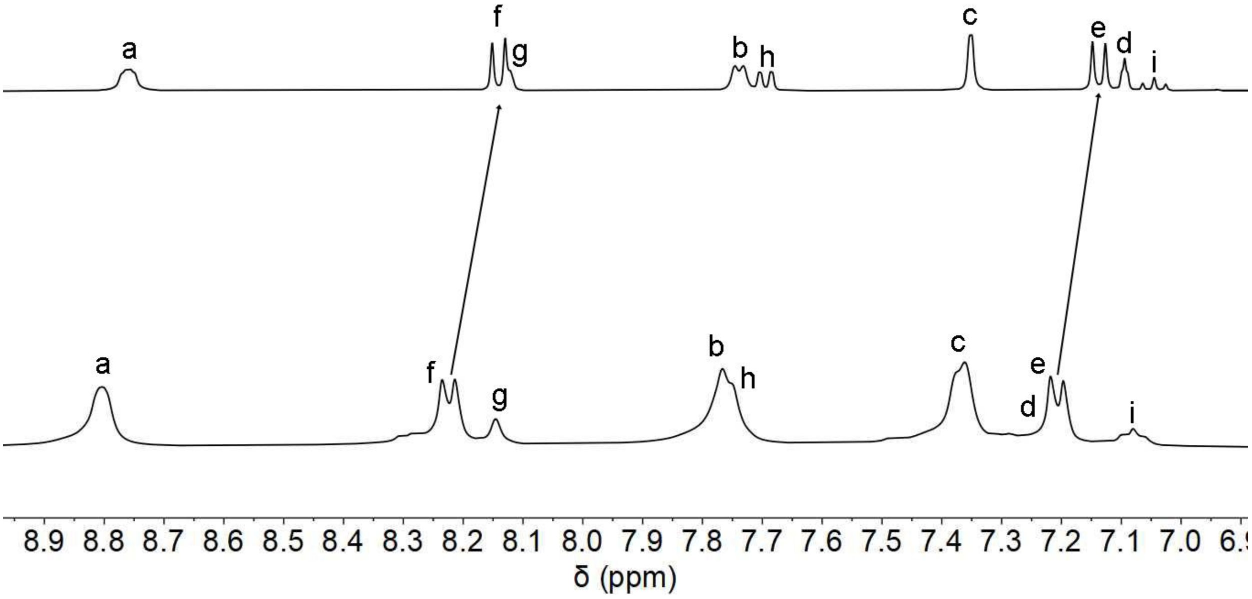

Supplementary Figure 35. ^{1}H NMR spectra (400 MHz, CD_2Cl_2, 298K) of NaOTf ⊂ **6** (bottom) and **6** (top).

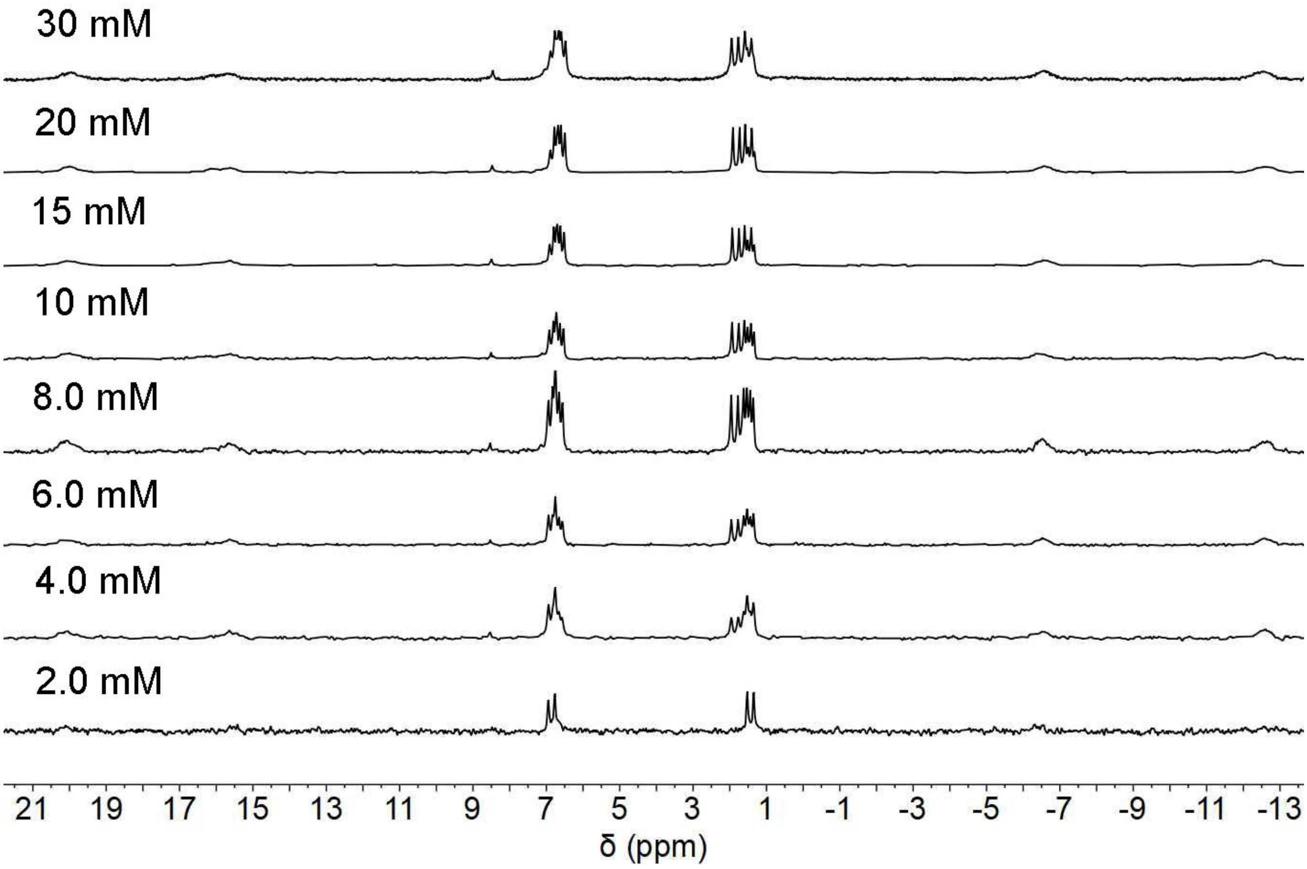

Supplementary Figure 36. $^{31}P\{^{1}H\}$ NMR (160 MHz, acetone-d_6, 298K) spectra of **6** at different concentrations.

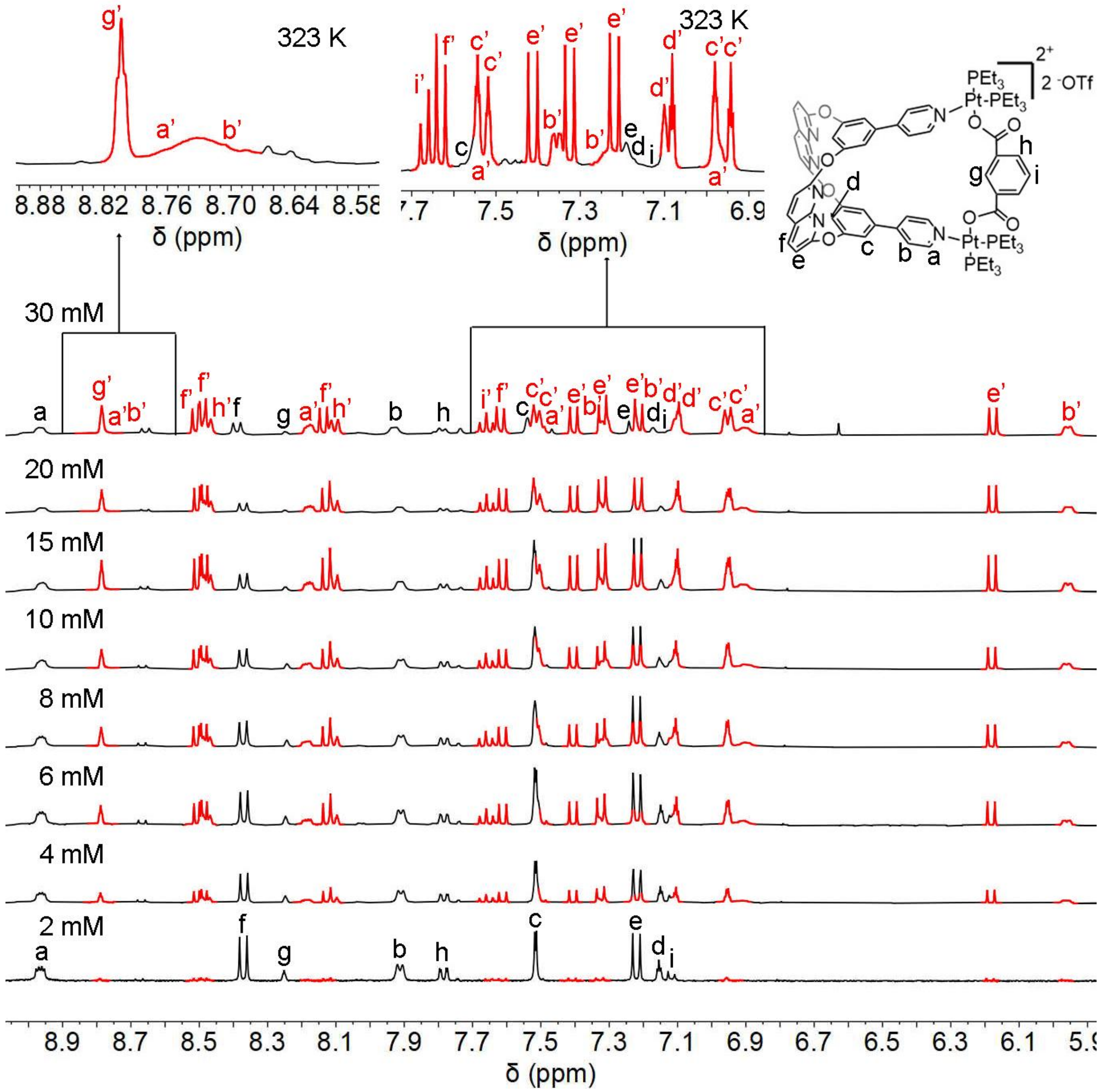

Supplementary Figure 37. Partial ^{1}H NMR (400 MHz, acetone-d_6, 298K) spectra of **6** at different concentrations.

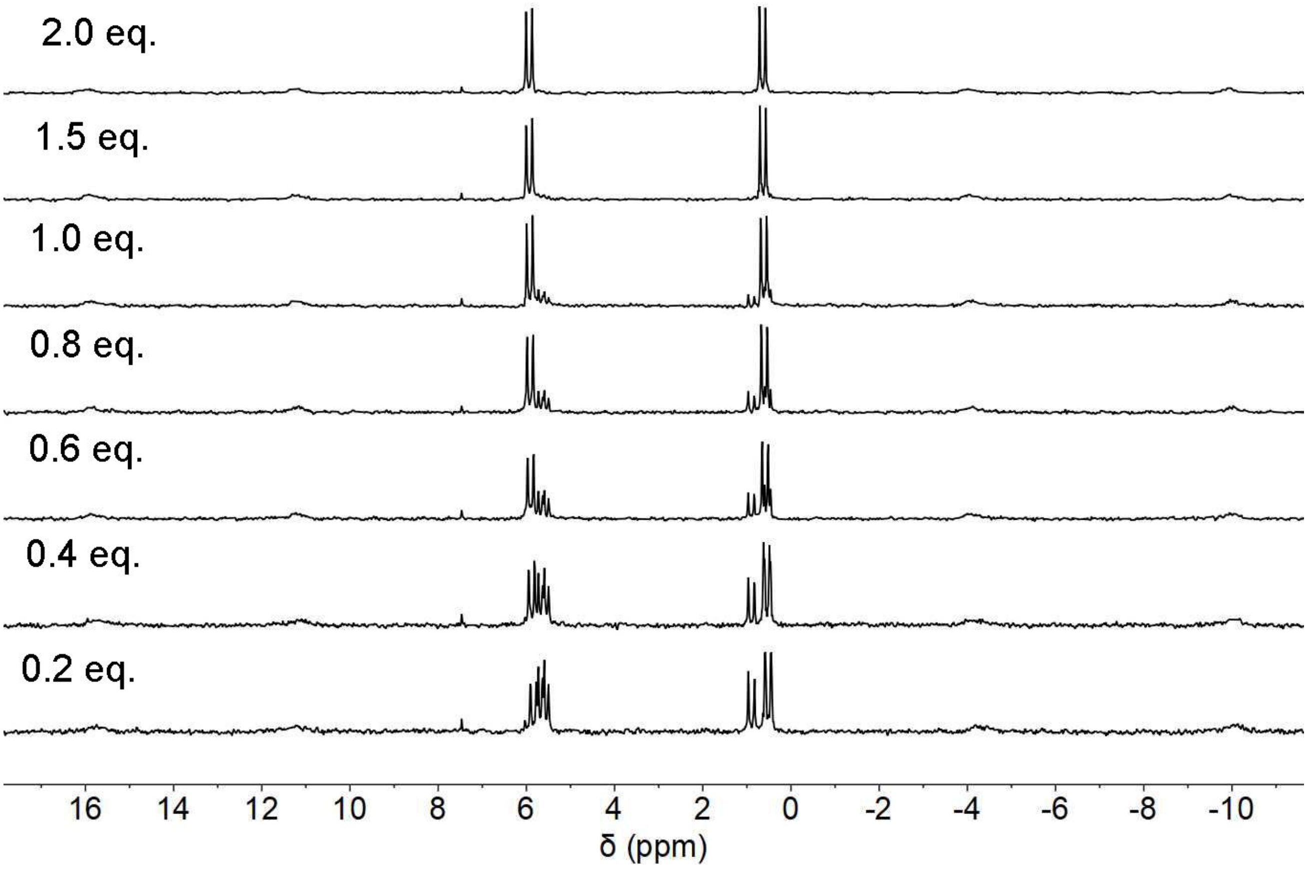

Supplementary Figure 38. $^{31}P\{^{1}H\}$ NMR (160 MHz, acetone-d_6, 298K) spectra of **6** (30 mM) with different eq. of NaOTf.

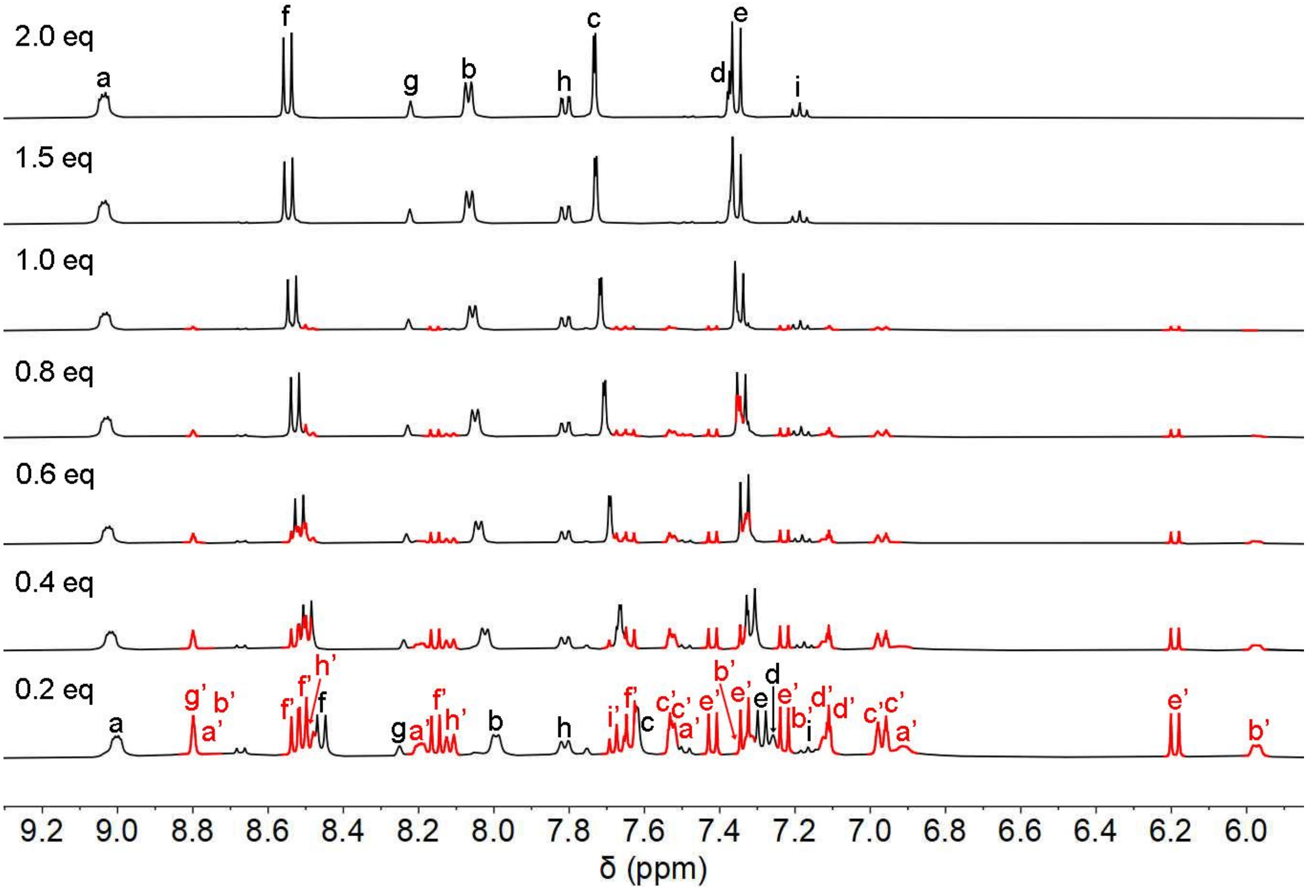

Supplementary Figure 39. Partial ^{1}H NMR (400 MHz, acetone-d_6, 298K) spectra of **6** (30 mM) with different eq. of NaOTf.

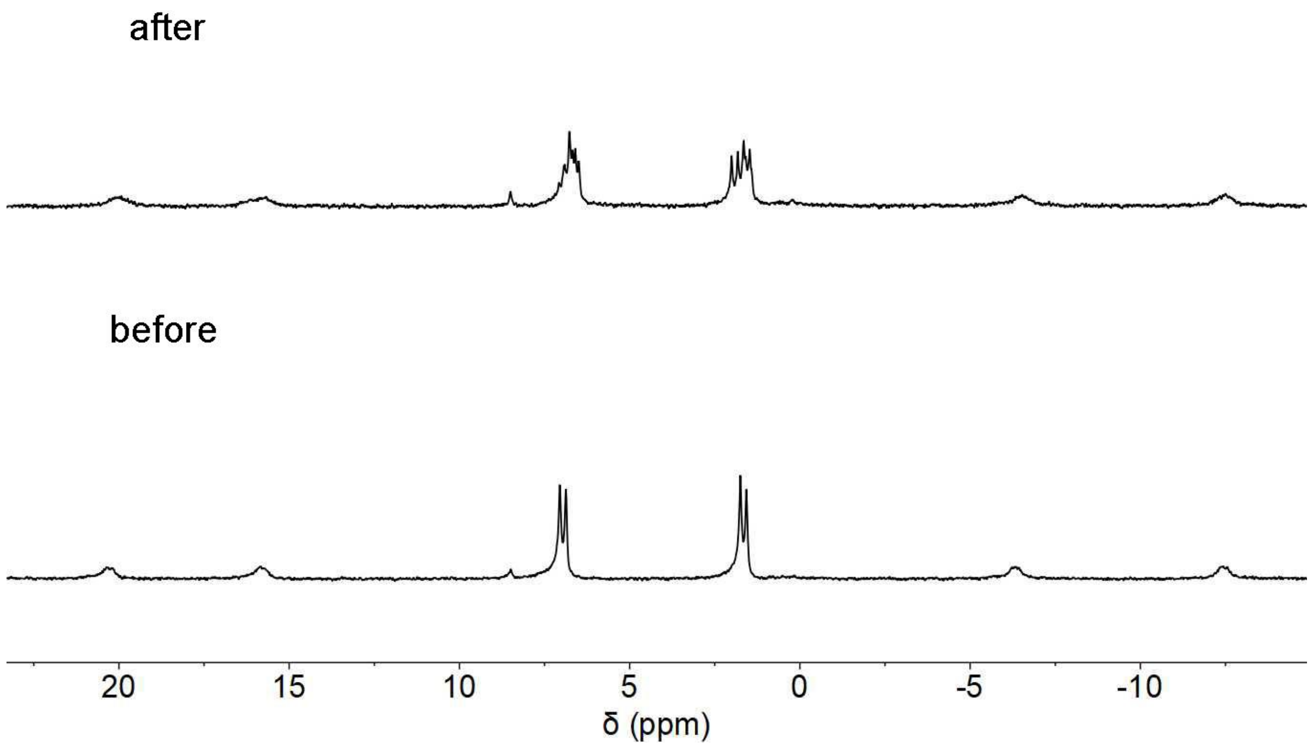

Supplementary Figure 40. $^{31}P\{^{1}H\}$ NMR (121 MHz, acetone-d_6, 298K) spectra of **6** (30 mM) with 1.5 eq. NaOTf: before and after the addition of 18C6 (1.8 eq.).

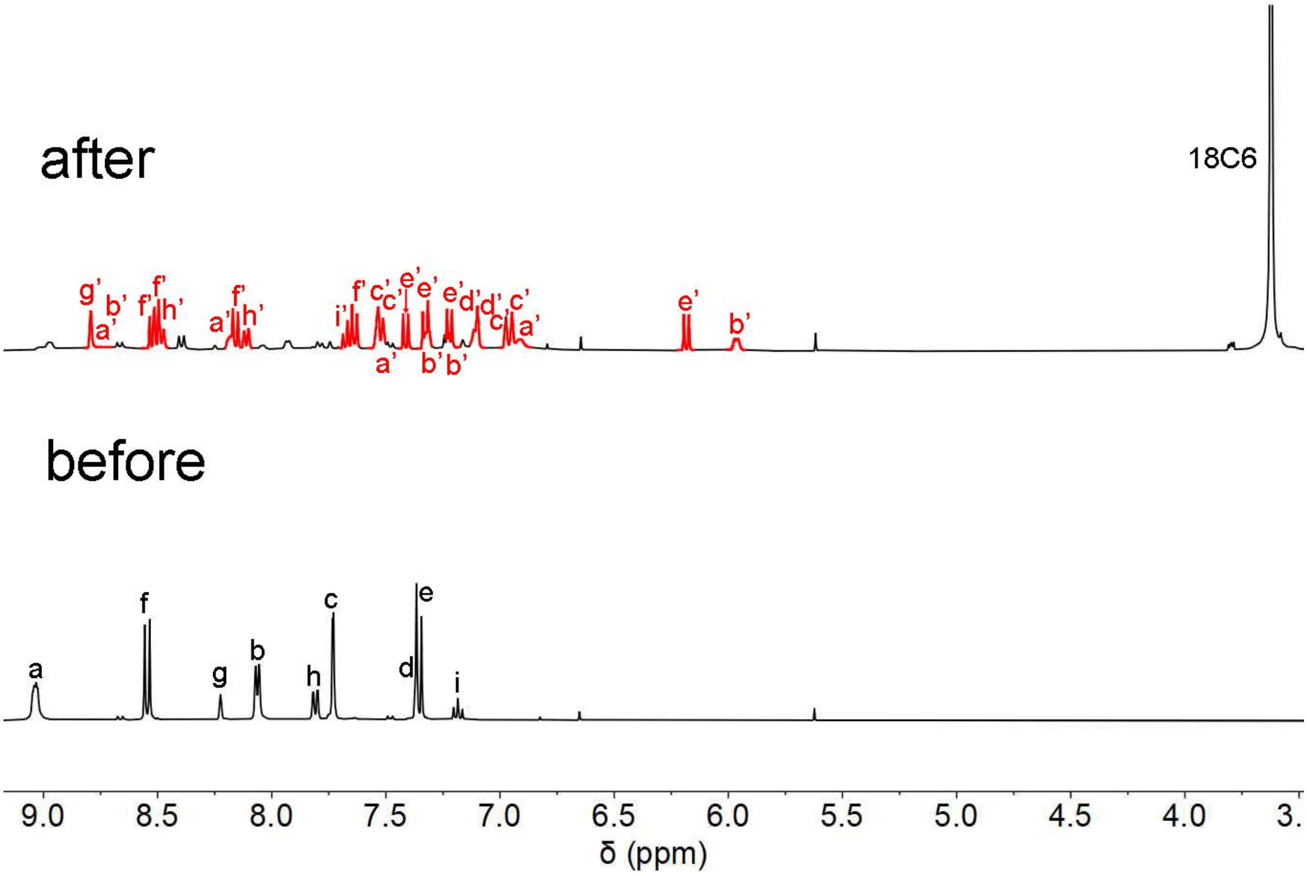

Supplementary Figure 41. Partial ^{1}H NMR (400 MHz, acetone-d_6, 298K) spectra of **6** (30 mM) with 1.5 eq. NaOTf: before and after the addition of 18C6 (1.8 eq.).

Upon addition of 1.5 eq. of NaOTf, the ^{31}P{^{1}H} NMR (Supplementary Figure 38) spectra show a clear transformation from four sets of coupled doublets for **7**, into two sets of coupled doublets for NaOTf ⊂ **6**. And the ^{1}H NMR (Supplementary Figure 39) spectra also indicate a structural transformation from **7** to NaOTf ⊂ **6**. Then, we expected that the sodium can be removed from the cavity of **6** with the assistance of 18-crown-6-ether (18C6), resulting in the reassembly of **7**. When we added 1.8 eq. 18C6 to the mixture of **6** and NaOTf, the NMR signals almost recovered the resonances of **7** (Supplementary Figure 40 & Supplementary Figure 41), indicating that the NaOTf- and 18C6-triggered structural inversions are reversible.

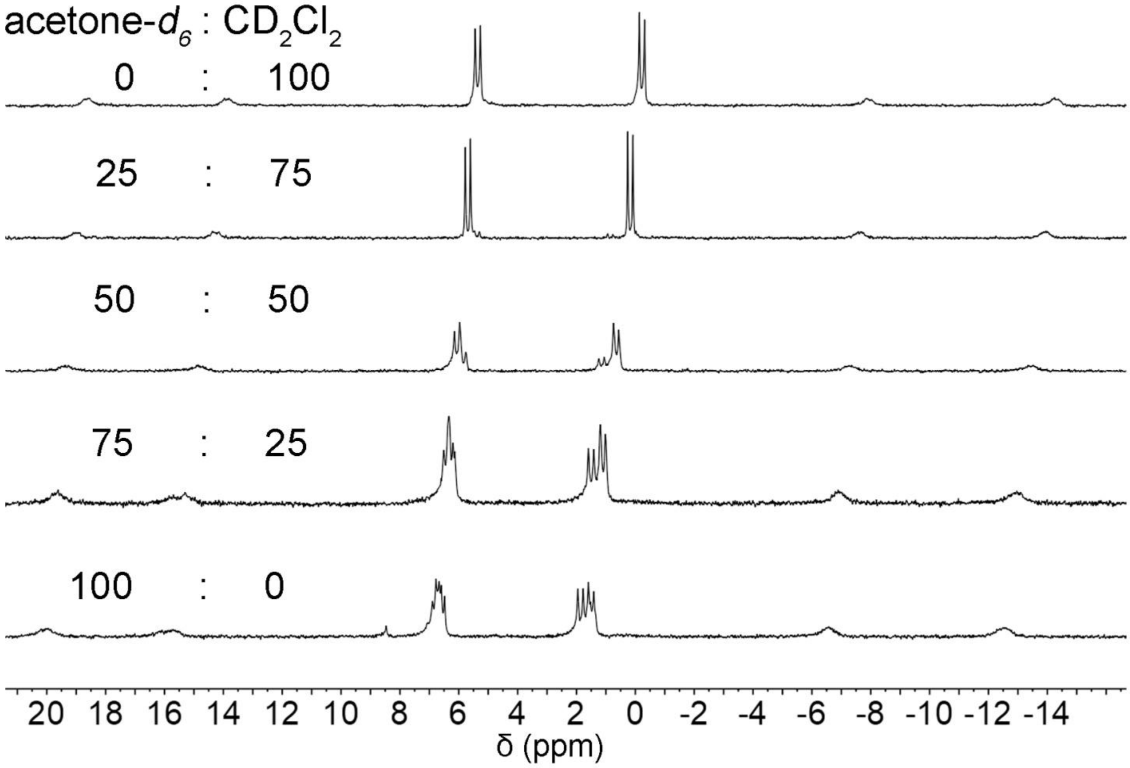

Supplementary Figure 42. $^{31}P\{^{1}H\}$ NMR (121 MHz, 298K) spectra of **6** (30 mM) in mixing solvent of acetone-d_6 and CD_2Cl_2.

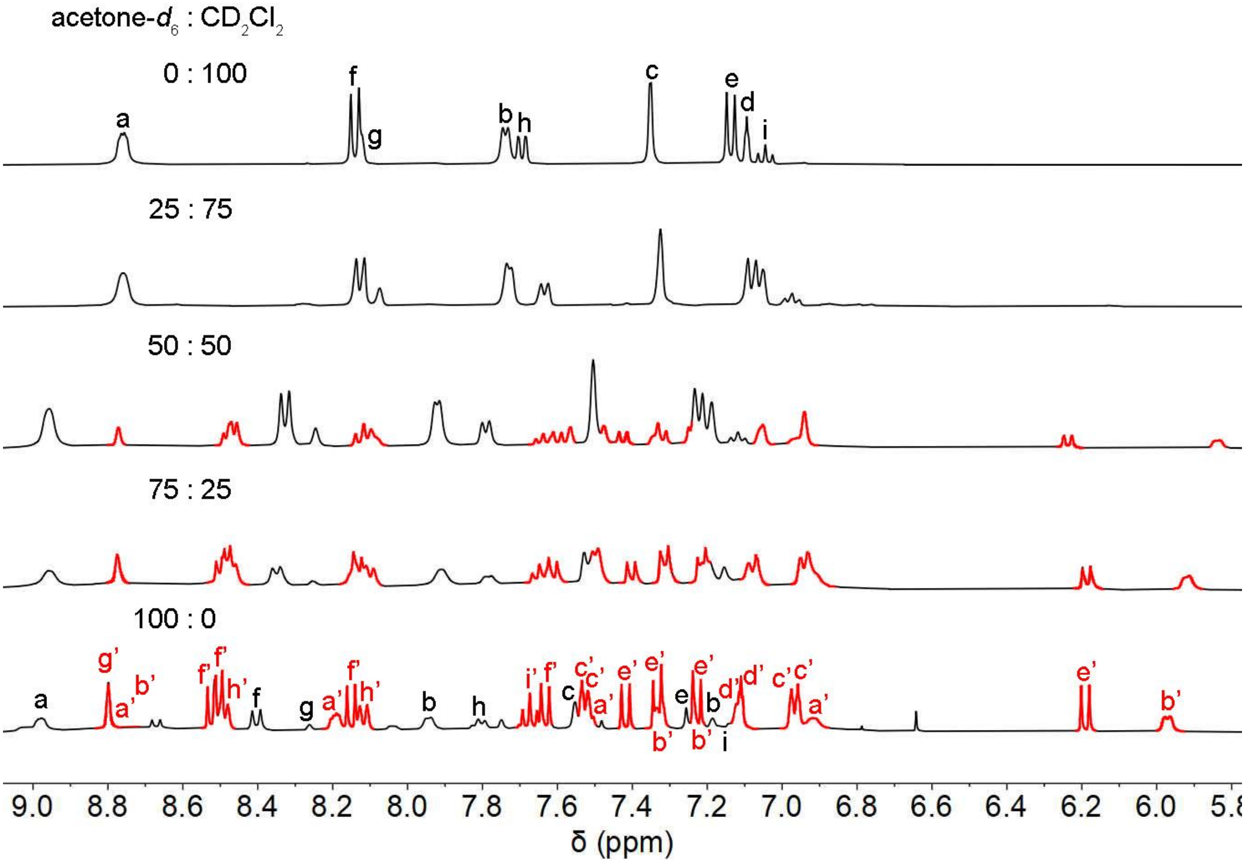

Supplementary Figure 43. Partial ^{1}H NMR (400 MHz, 298K) spectra of **6** (30 mM) in mixing mixing solvent of acetone-d_6 and CD_2Cl_2.

When the proportion of CD_2Cl_2 in a acetone-d_6/CD_2Cl_2 solution of **7** increase, the $^{31}P\{^1H\}$ NMR (Supplementary Figure 42) and ^1H NMR (Supplementary Figure 43) spectra clearly show that the nuclear resonances of **7** are gradually replaced by the signals for **6**. While **6** is dissolved in CD_2Cl_2 at 30 mM, only the resonances for the monomeric metallacage **6** is observed by NMR spectroscopy (Supplementary Figure 42 & Supplementary Figure 43), indicating that **7** could not be formed in CD_2Cl_2.

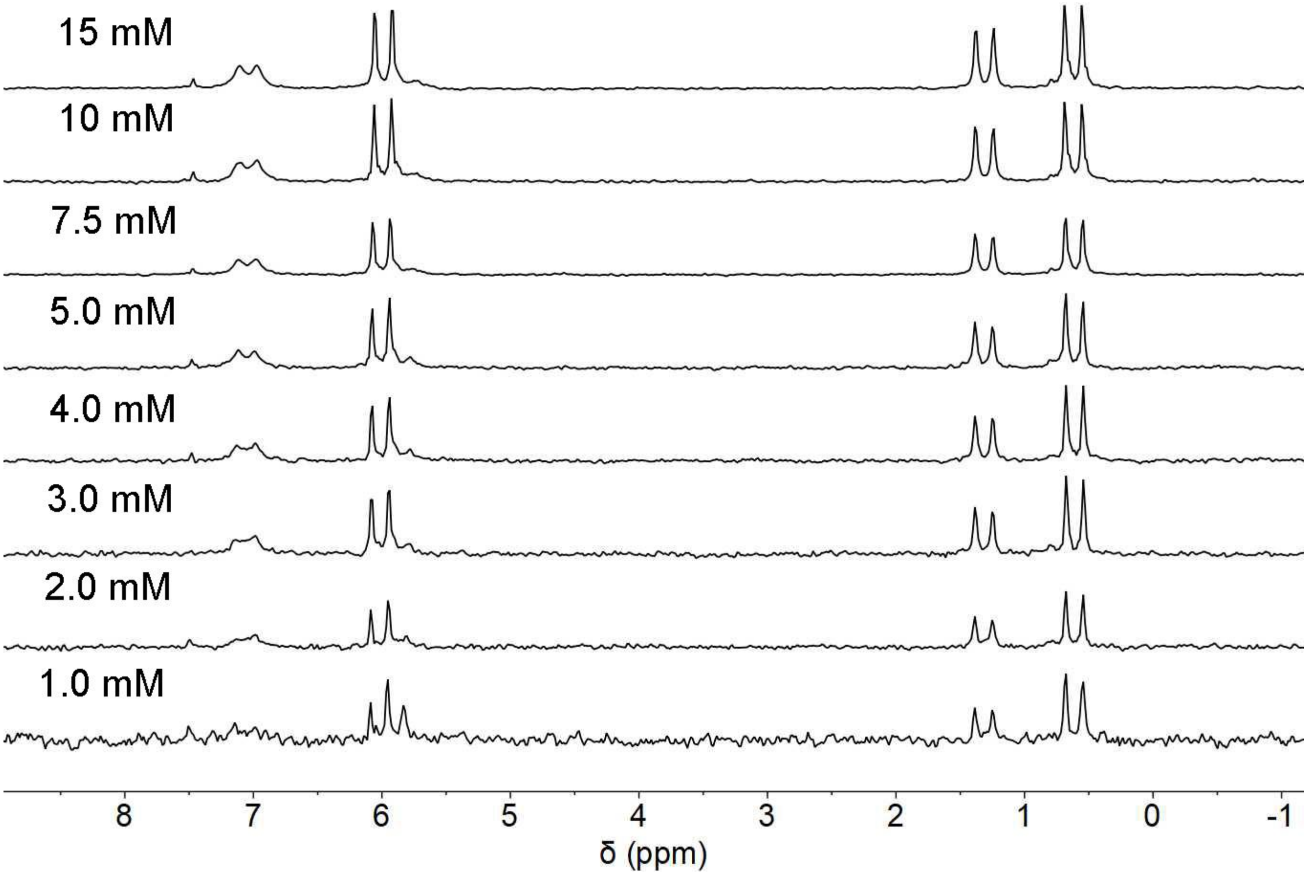

Supplementary Figure 44. $^{31}P\{^1H\}$ NMR spectra (160 MHz, 298 K) of **9** in acetone-d_6 at different concentrations.

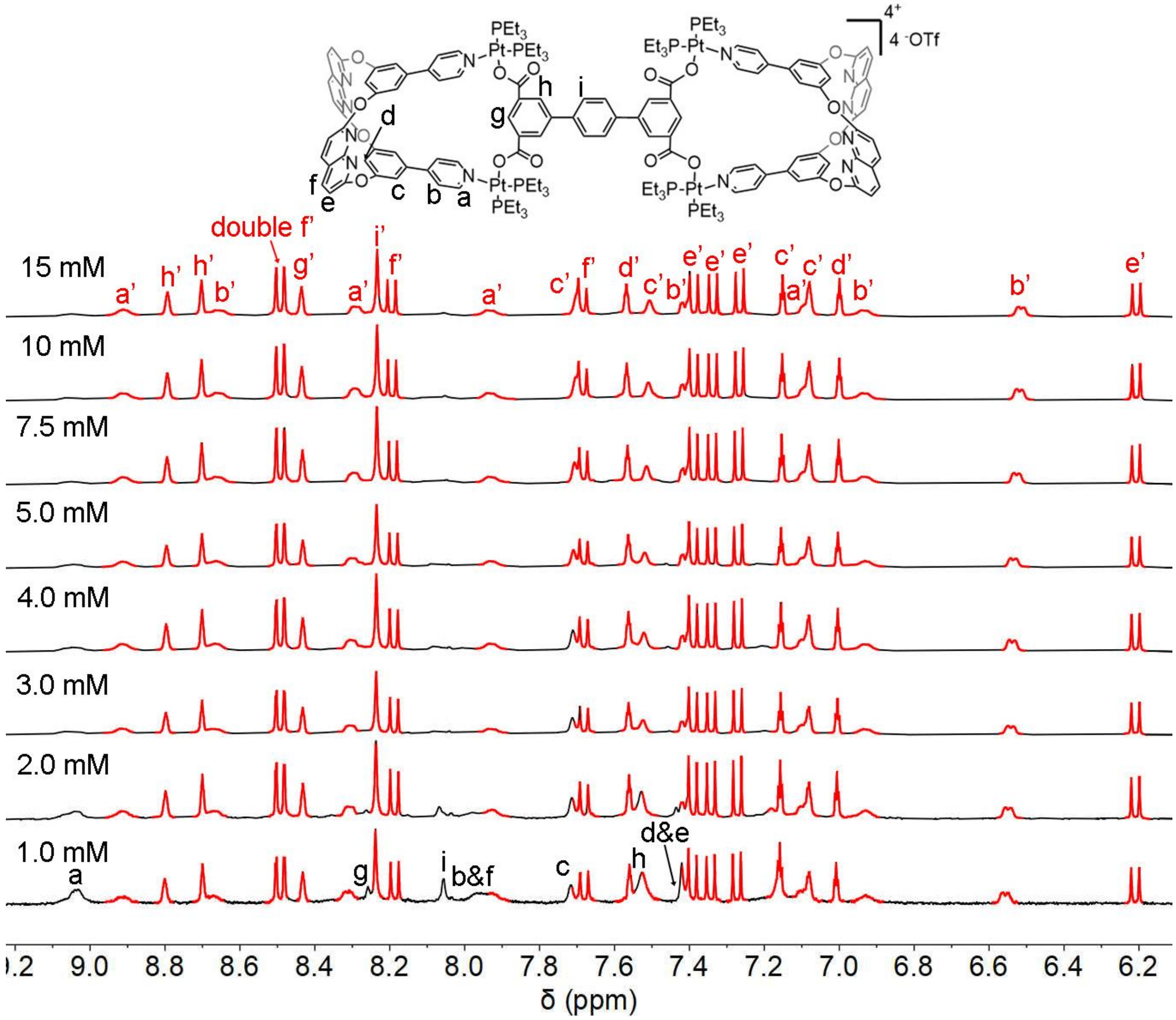

Supplementary Figure 45. Partial ^1H NMR (400 MHz, acetone-d_6, 298K) spectra of **9** in acetone-d_6 at different concentrations.

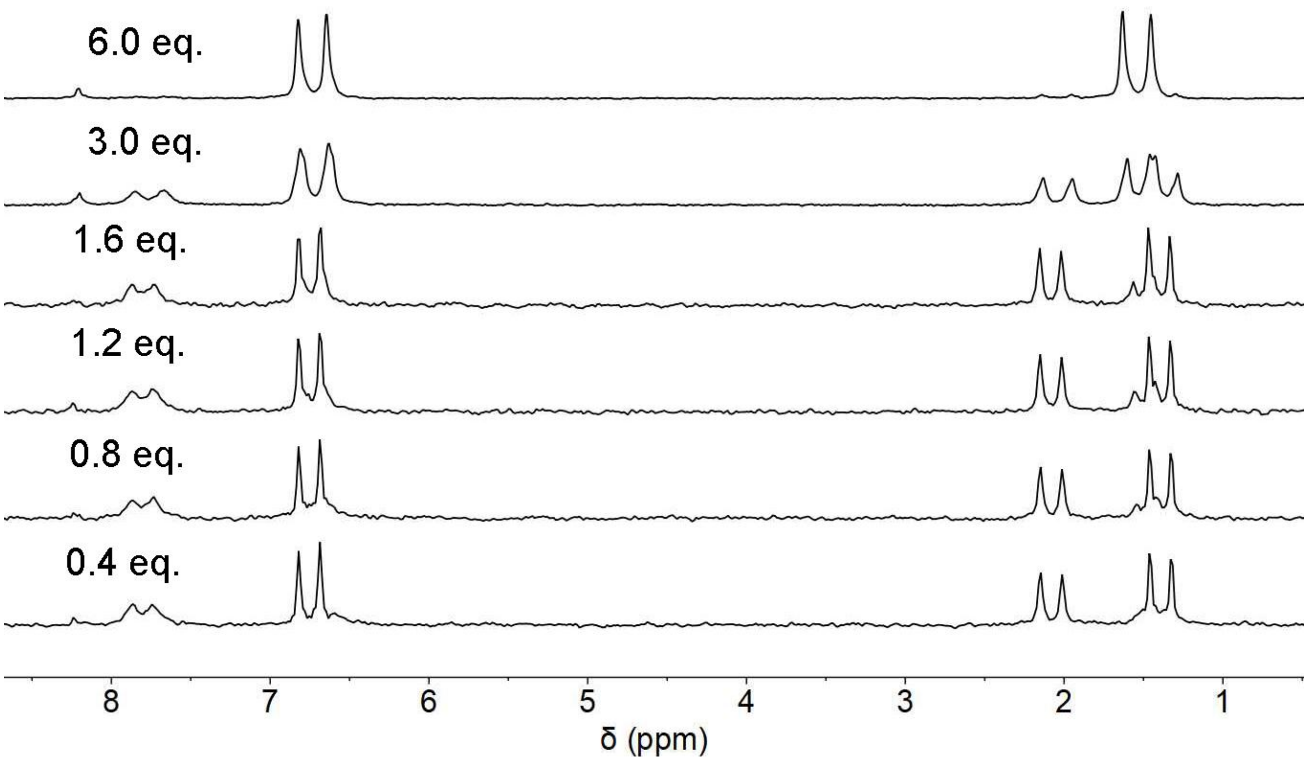

Supplementary Figure 46. $^{31}P\{^{1}H\}$ NMR spectra (160 MHz, acetone-d_6, 298 K) of **9** (15 mM) with different eq. of NaOTf.

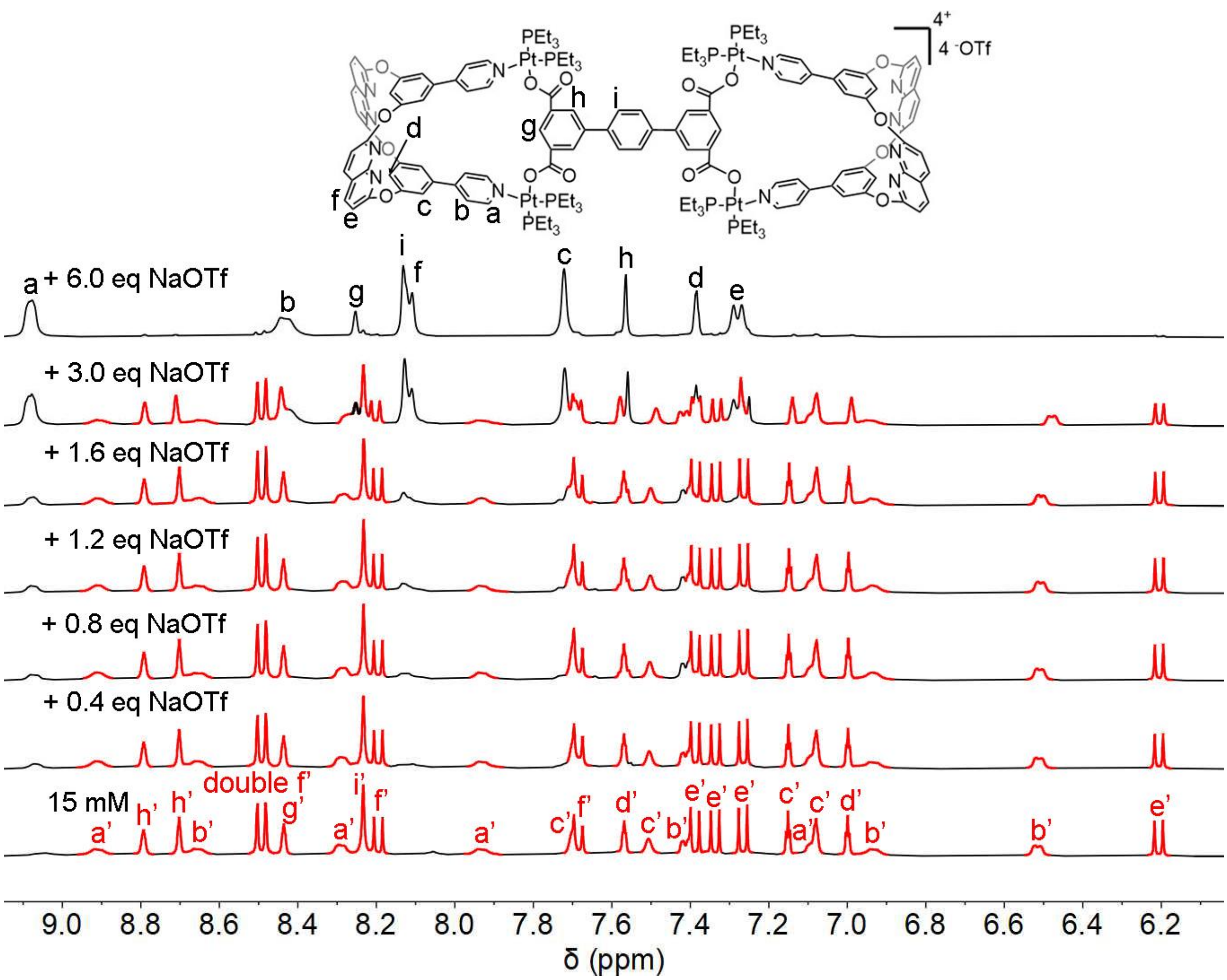

Supplementary Figure 47. Partial ^{1}H NMR (400 MHz, acetone-d_6, 298K) spectra of **9** (15 mM) with different eq. of NaOTf.

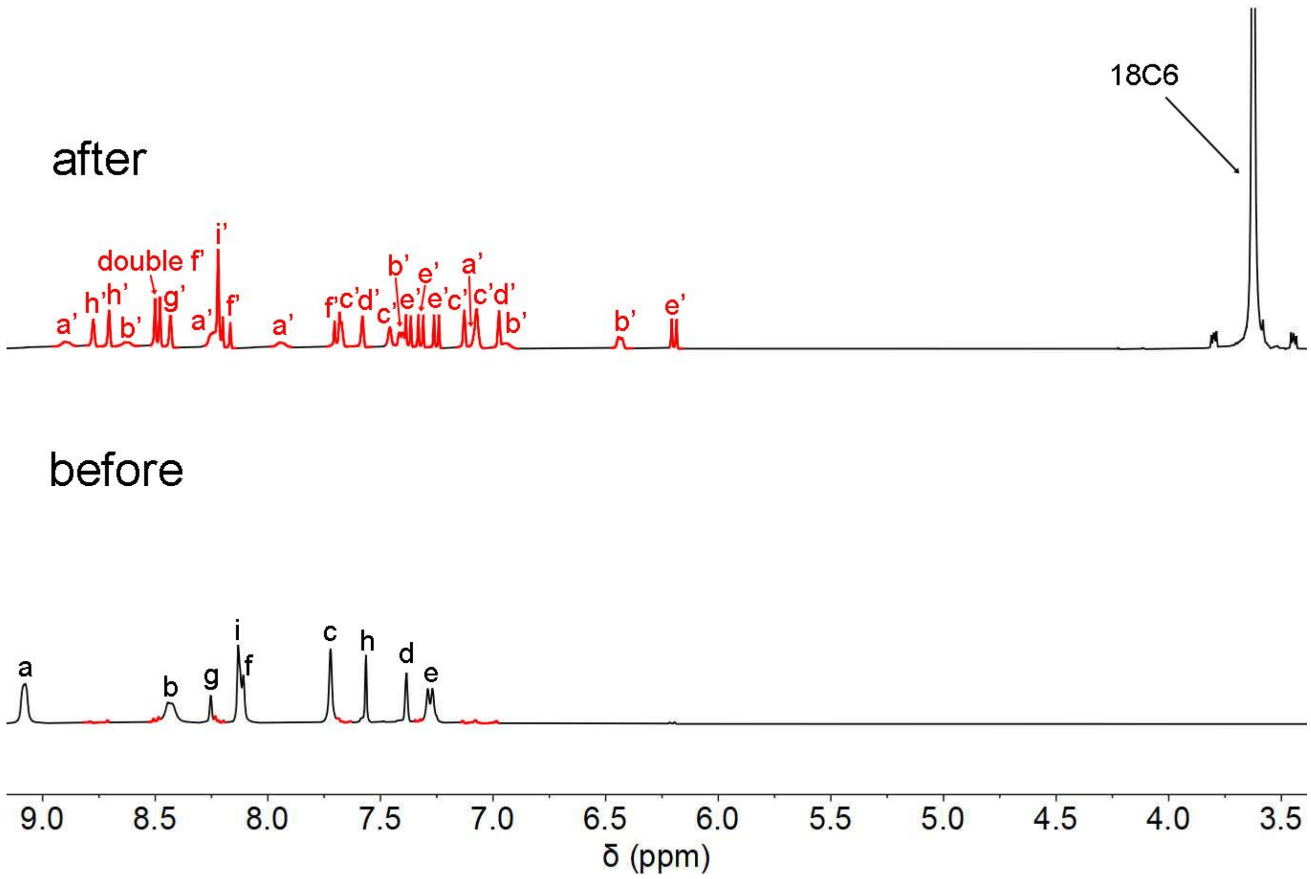

Supplementary Figure 48. Partial ^{1}H NMR (400 MHz, acetone-d_6, 298K) spectra of **9** (15 mM) with 6.0 eq. NaOTf: before and after the addition of 18C6 (7.0 eq.).

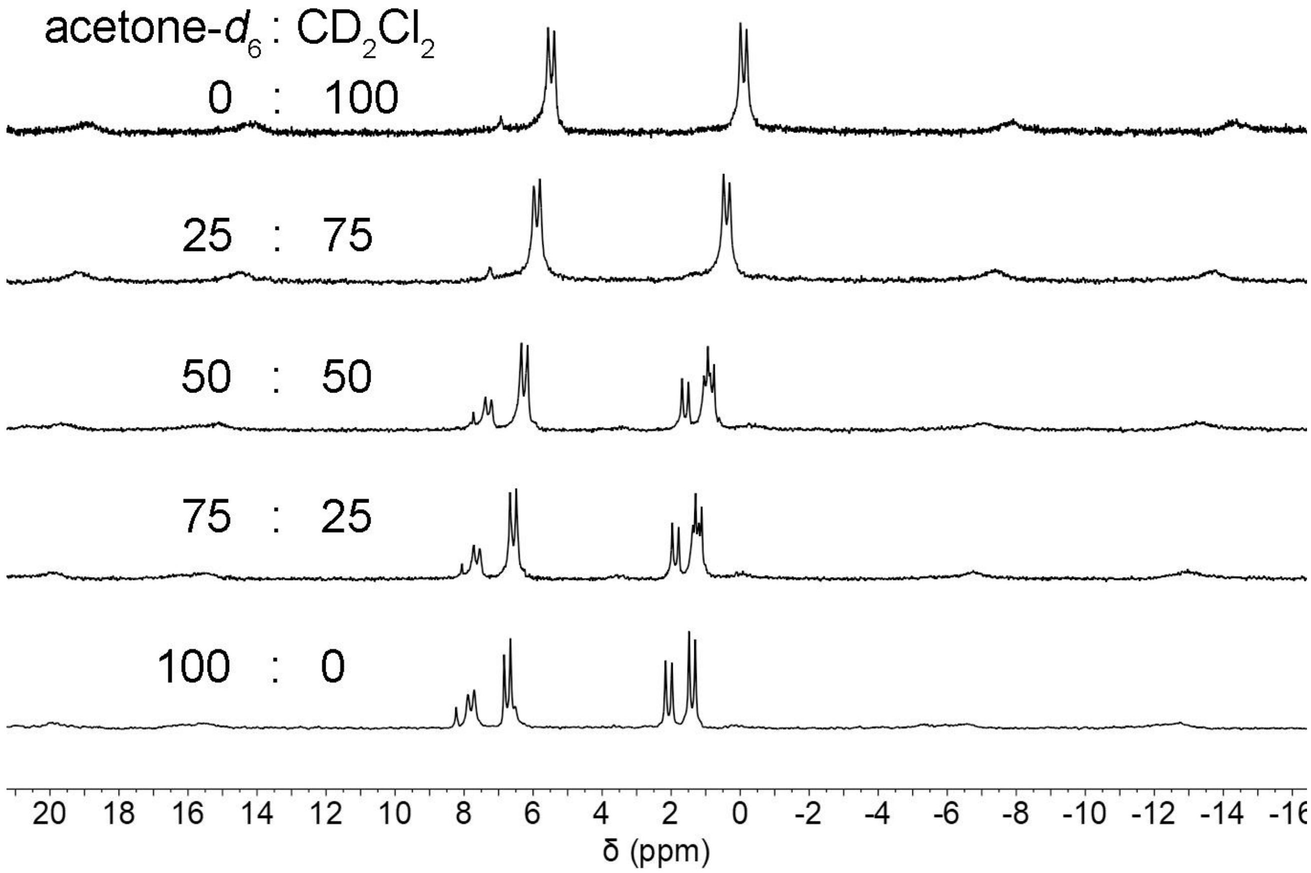

Supplementary Figure 49. $^{31}P\{^{1}H\}$ NMR (121 MHz, 298 K) spectra of **9** (15 mM) in a mixing solvent of acetone-d_6/CD_2Cl_2 (v/v).

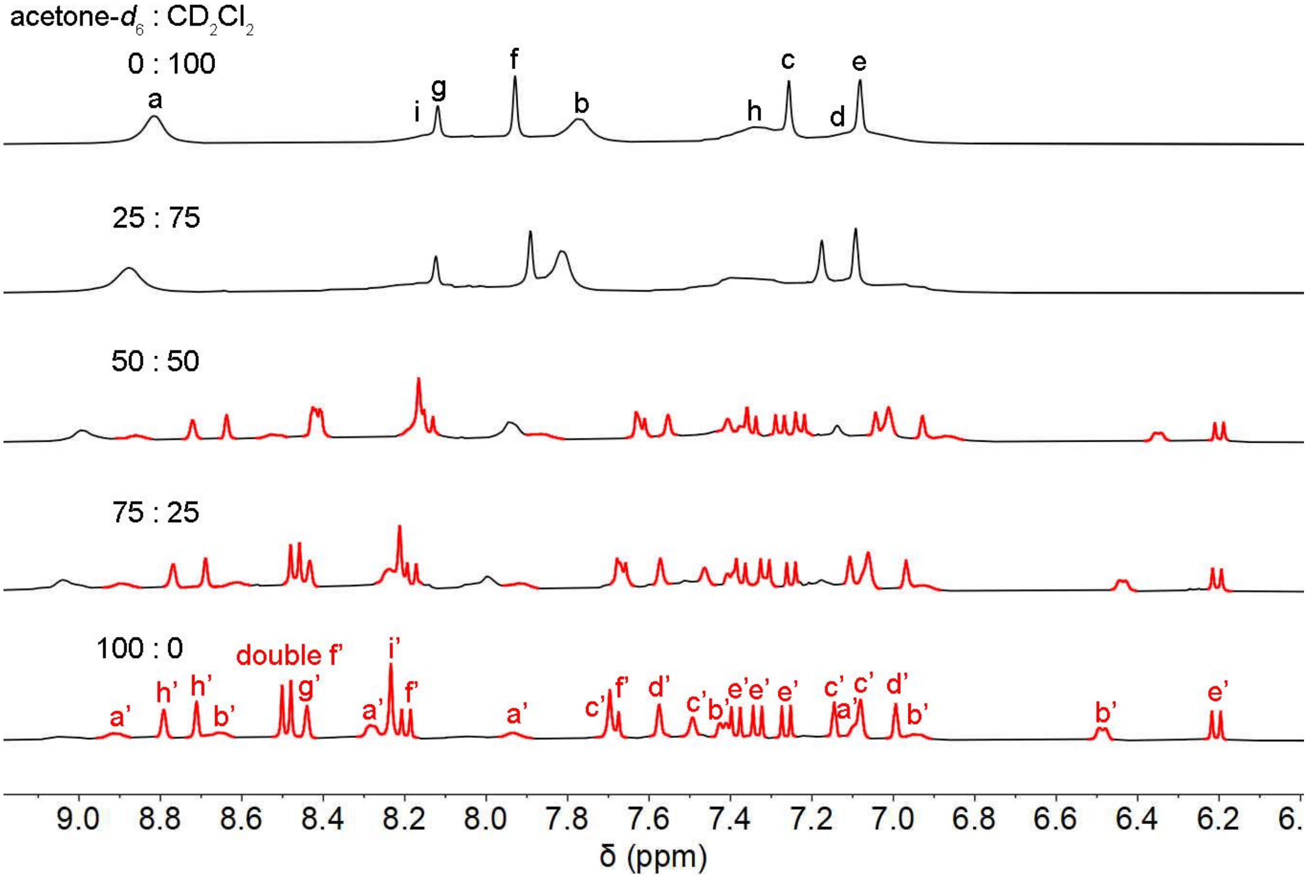

Supplementary Figure 50. Partial ^{1}H NMR (400 MHz, 298K) spectra of **9** (15 mM) in mixing solvent of acetone-d_6 and CD_2Cl_2.

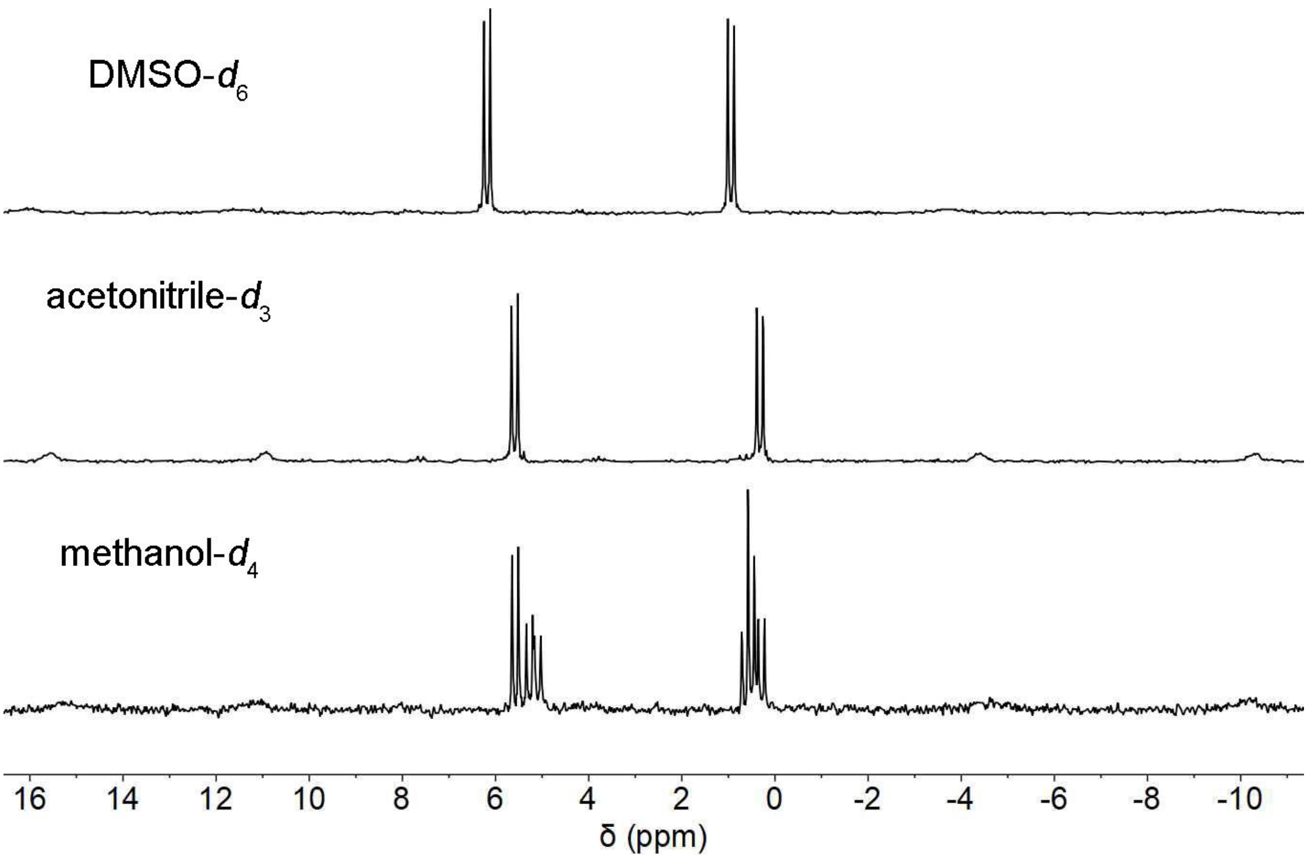

Supplementary Figure 51. $^{31}P\{^{1}H\}$ NMR (160 MHz, 298 K) spectra of **6** in DMSO-d_6 (30 mM), acetonitrile-d_3 (30 mM), and methanol-d_4 (10 mM). Due to the low solubility, **6** in methanol-d_4 was tested at the concentration of 10 mM.

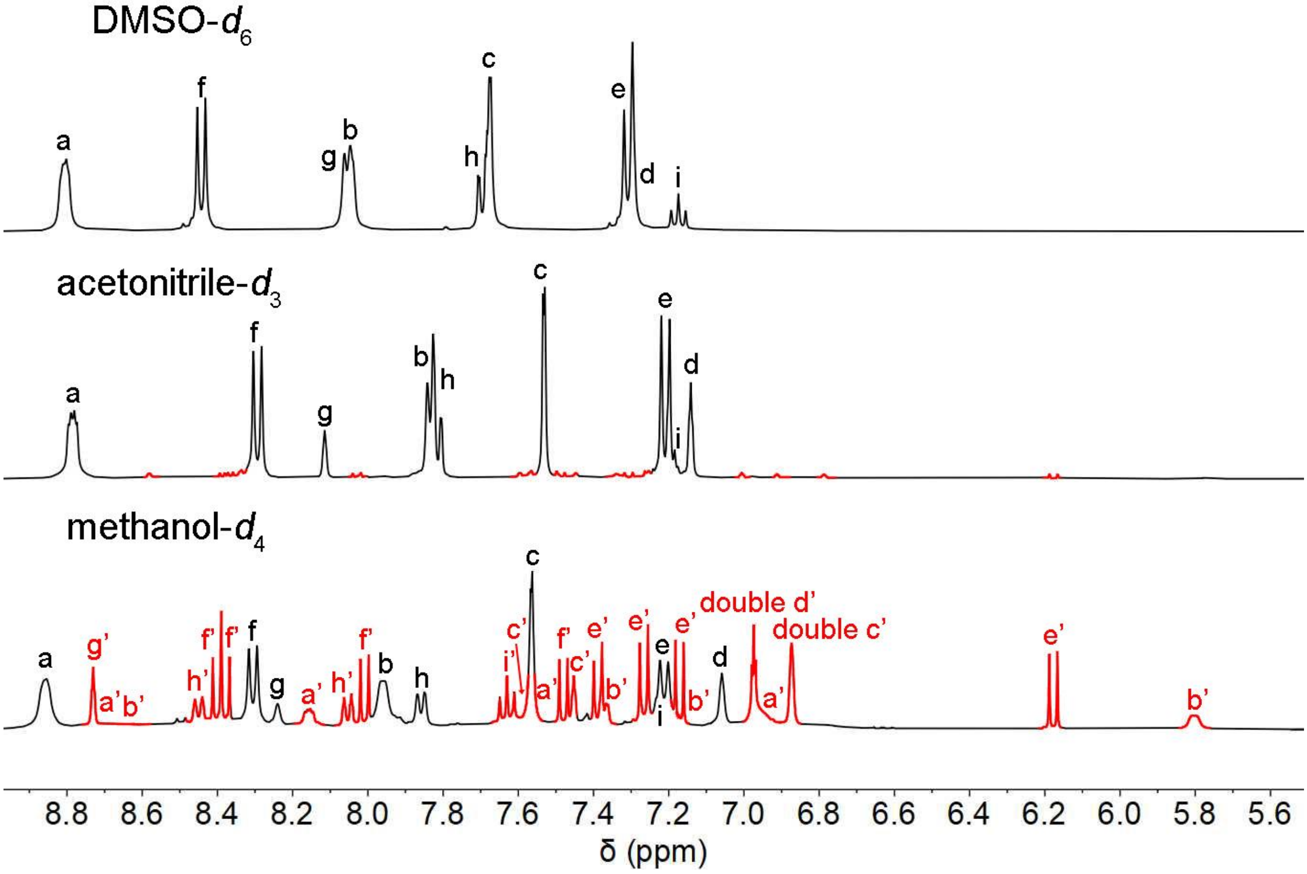

Supplementary Figure 52. Partial ^{1}H NMR (400 MHz, 298K) spectra of **6** in DMSO-d_6 (30 mM), acetonitrile-d_3 (30 mM), and methanol-d_4 (10 mM). Due to the low solubility, **6** in methanol-d_4 was tested at the concentration of 10 mM.

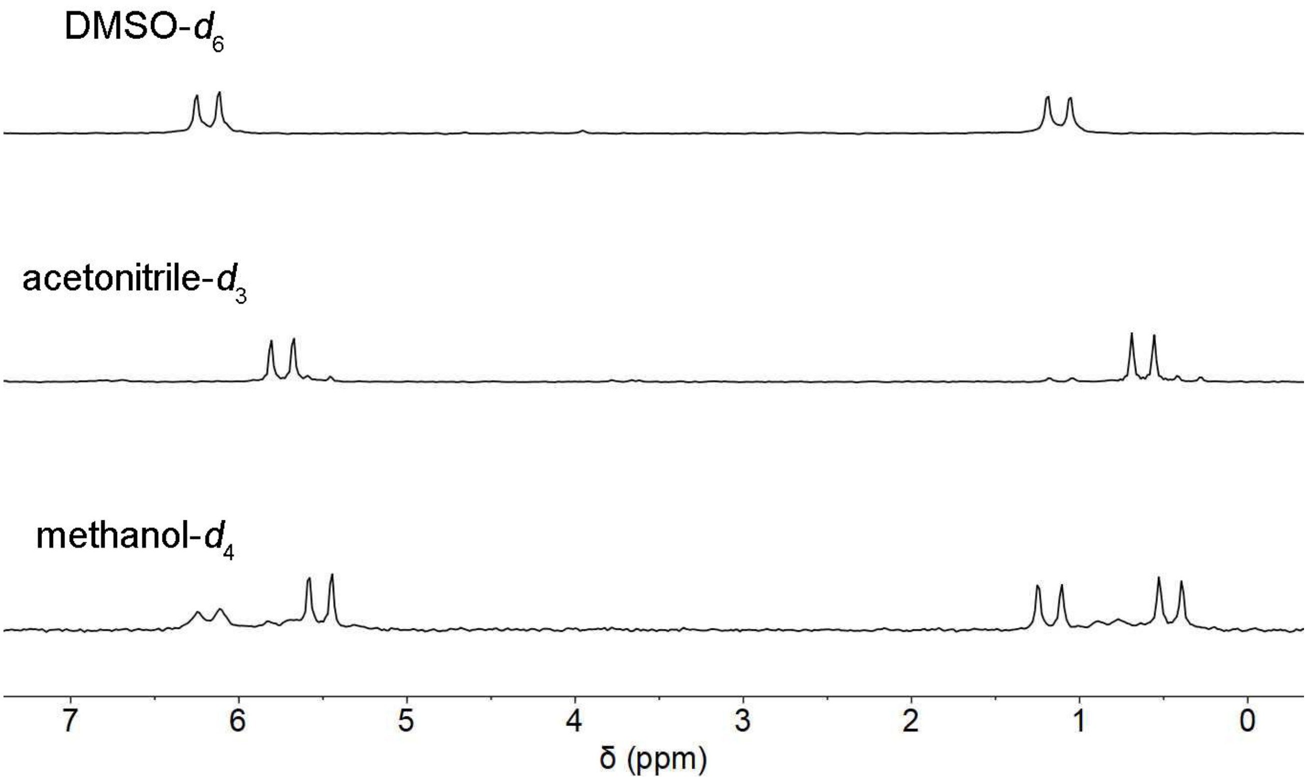

Supplementary Figure 53. $^{31}P\{^1H\}$ NMR (160 MHz, 298 K) spectra of **9** (15 mM) in DMSO-d_6, acetonitrile-d_3, and methanol-d_4.

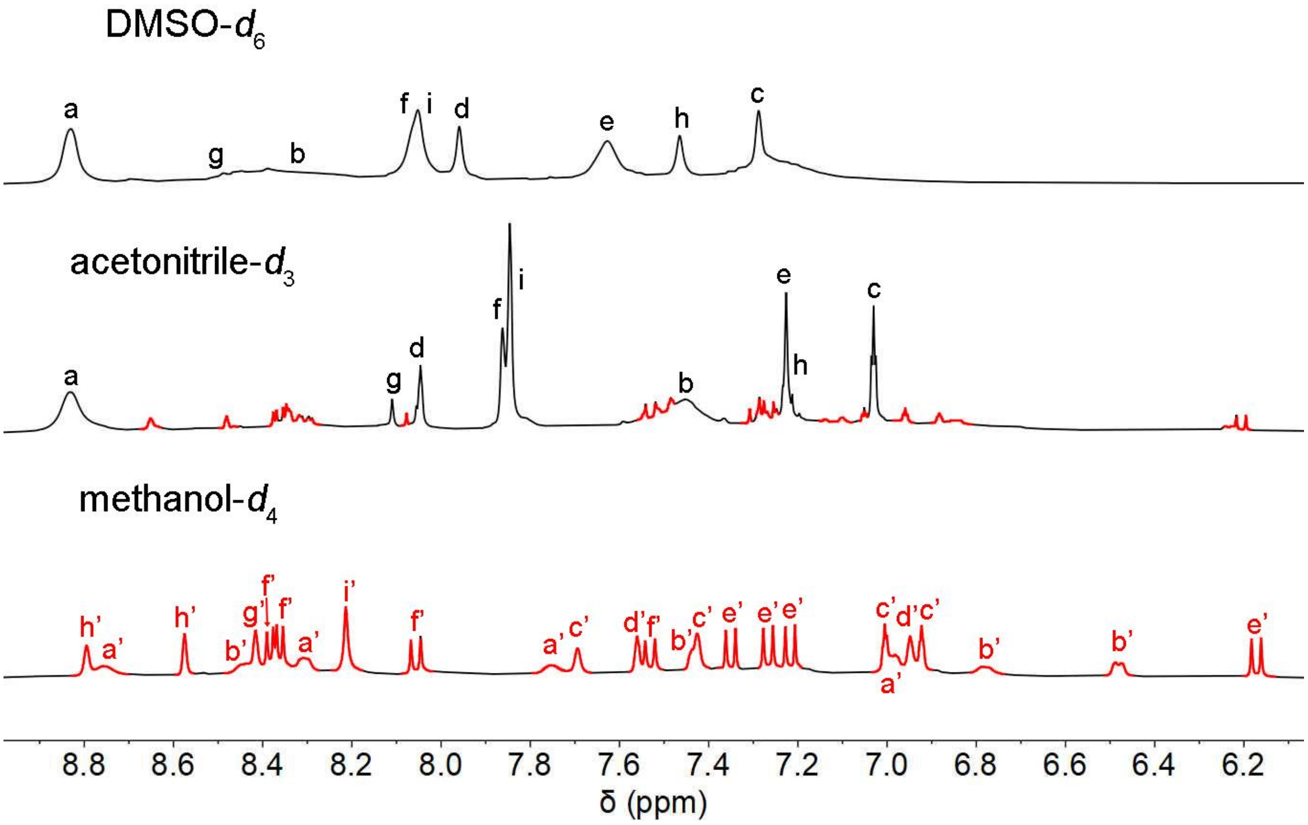

Supplementary Figure 54. Partial ^{1}H NMR (400 MHz, 298K) spectra of **9** (15 mM) in DMSO-d_6, acetonitrile-d_3, and methanol-d_4.

Supplementary Table 6. Proportions of **6**/**7** at different concentrations of **6** in acetone-d_6.

Concentration of **6** (mM) in acetone-d_6	Percentage of **6**	Percentage of **7**
2	89.3	10.7
4	70.7	29.3
6	60.1	39.9
8	47.2	52.8
10	32.1	67.9
15	23.1	76.9
20	19.5	80.5
30	18.2	81.8

Supplementary Table 7. Proportions of **6**/**7** in acetone-d_6 solution of **6** at 30 mM in the presence of NaOTf.

Addition of NaOTf (eq.)	Percentage of **6**	Percentage of **7**
0.2	37.9	62.1
0.4	54.3	45.7
0.6	73.5	26.5
0.8	80.6	19.4

1	83.3	16.7
1.5	92.6	7.4
2	>95.0	<5.0

Supplementary Table 8. Proportions of **6**/**7** in different solvents of **6** at 30 mM.

Solvents	Percentage of **6**	Percentage of **7**
Acetone-d_6/CD_2Cl_2 (v/v, 100/0)	18.2	81.8
Acetone-d_6/CD_2Cl_2 (v/v, 75/25)	38.5	61.5
Acetone-d_6/CD_2Cl_2 (v/v, 50/50)	61.0	39.0
Acetone-d_6/CD_2Cl_2 (v/v, 25/75)	92.6	7.4
Acetone-d_6/CD_2Cl_2 (v/v, 0/100)	>95.0	<5.0
methanol-d_4[a]	54.3	45.7
acetonitrile-d_3	92.6	7.4
DMSO-d_6	>95.0	<5.0

[a]: Due to the lack of solubility, the concentration of **6** in methanol-d_4 is 10 mM.

Supplementary Table 9. Proportions of **9**/**10** at different concentrations of **9** in acetone-d_6.

Concentration of **9** (mM) in acetone-d_6	Percentage of **9**	Percentage of **10**
1	35.7	64.3
2	22.3	77.7
3	16.2	83.8
4	13.8	86.2
5	12.5	87.5
7.5	9.5	90.5
10	7.7	92.3
15	5.1	94.9

Supplementary Table 10. Proportions of **9**/**10** in acetone-d_6 solution of **9** at 15 mM in the presence of NaOTf.

Addition of NaOTf (eq.)	Percentage of **9**	Percentage of **10**
0.4	9.9	90.1
0.8	14.6	85.4
1.2	16.0	84.0
1.6	20.5	79.5
3	78.1	21.9
6	>95.0	<5.0

Supplementary Table 11. Proportions of **9**/**10** in different solvents of **9** at 15 mM.

Solvents	Percentage of **9**	Percentage of **10**
Acetone-d_6/CD_2Cl_2 (v/v, 100/0)	5.1	94.9
Acetone-d_6/CD_2Cl_2 (v/v, 75/25)	29.8	70.2
Acetone-d_6/CD_2Cl_2 (v/v, 50/50)	35.2	64.8
Acetone-d_6/CD_2Cl_2 (v/v, 25/75)	>95.0	<5.0

Acetone-d_6/CD_2Cl_2 (v/v, 0/100)	>95.0	<5.0
methanol-d_4	<5.0	>95.0
acetonitrile-d_3	84.7	15.3
DMSO-d_6	>95.0	<5.0

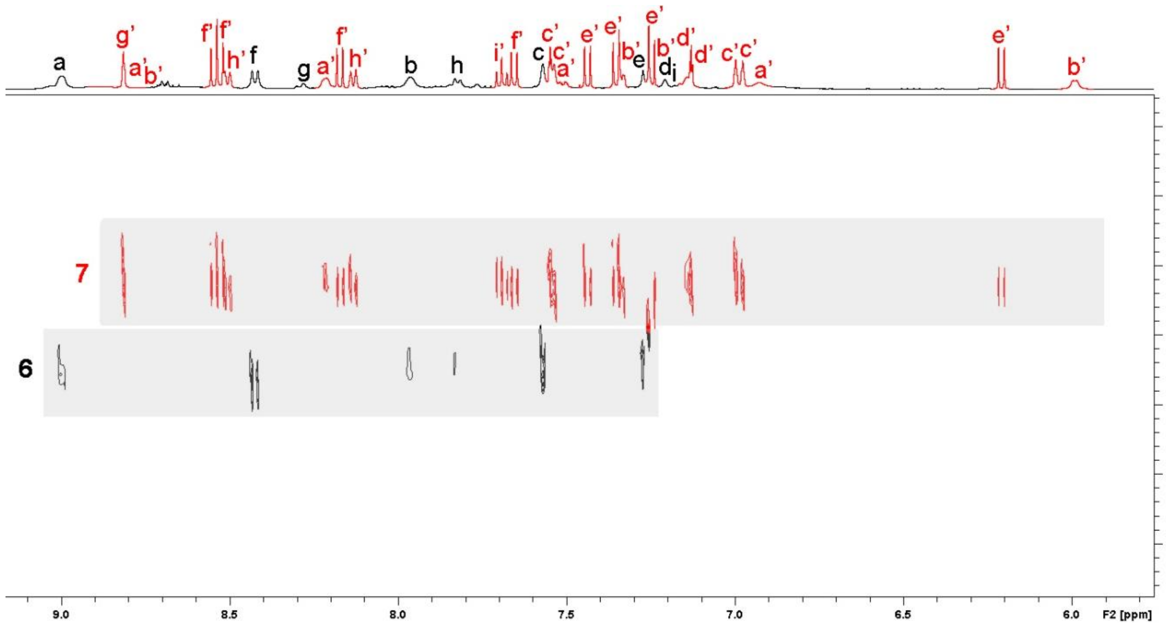

Supplementary Figure 55. DOSY NMR spectrum (500 MHz, acetone-d_6, 298 K) of **6/7**. (Concentration of **6**: 30 mM)

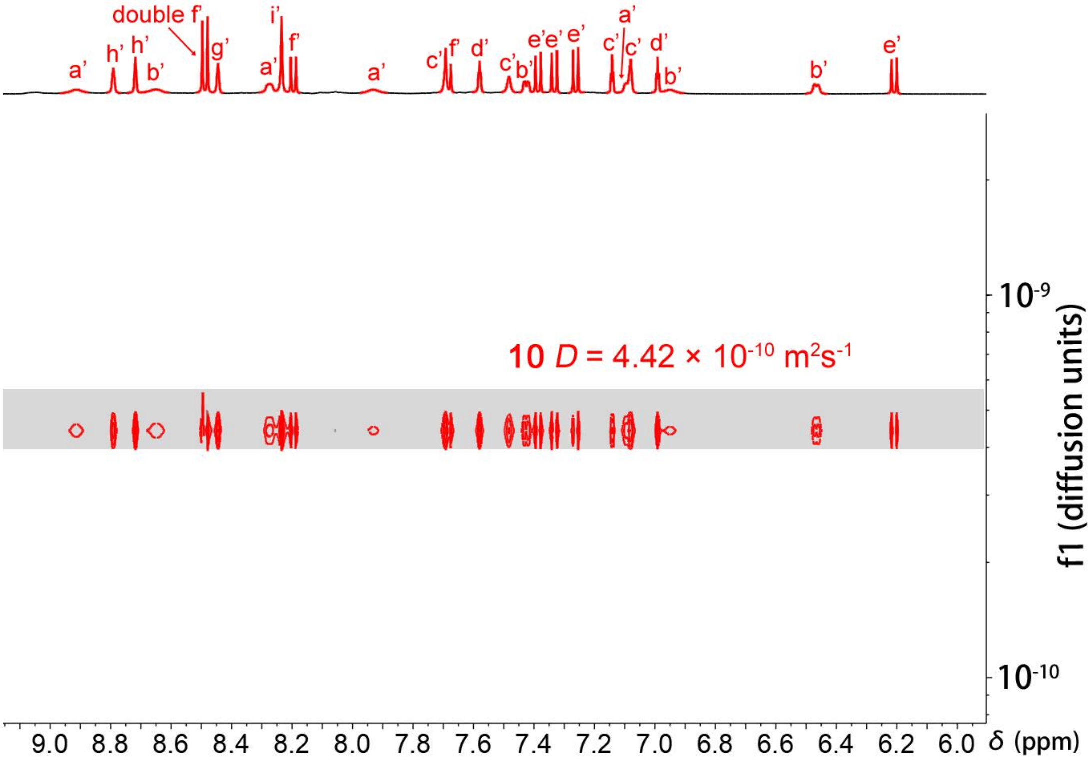

Supplementary Figure 56. DOSY NMR spectrum (500 MHz, acetone-d_6, 298 K) of **10**. (Concentration of **9**: 15 mM)

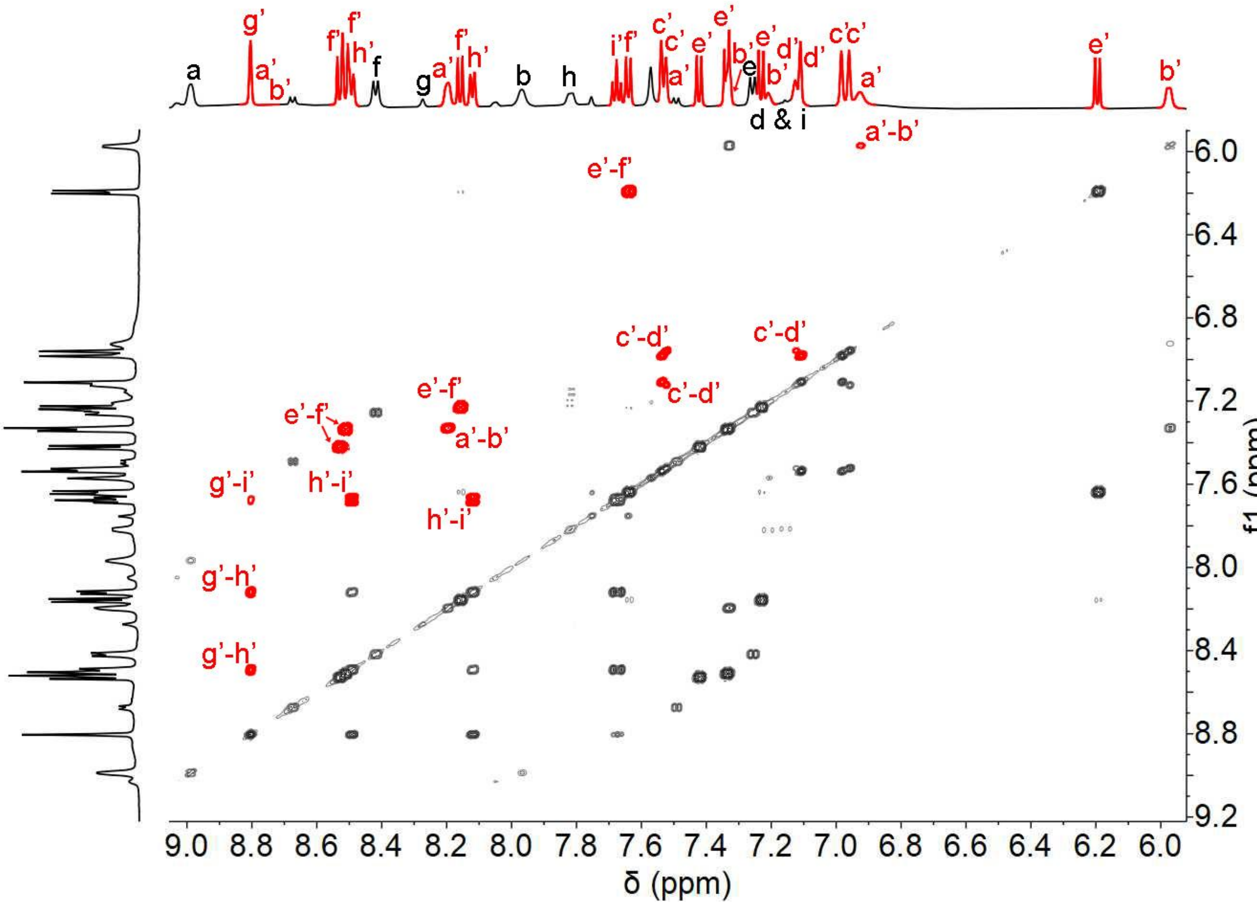

Supplementary Figure 57. ^{1}H-^{1}H COSY NMR spectrum (600 MHz, acetone-d_6, 298 K) of **6** at 30 mM.

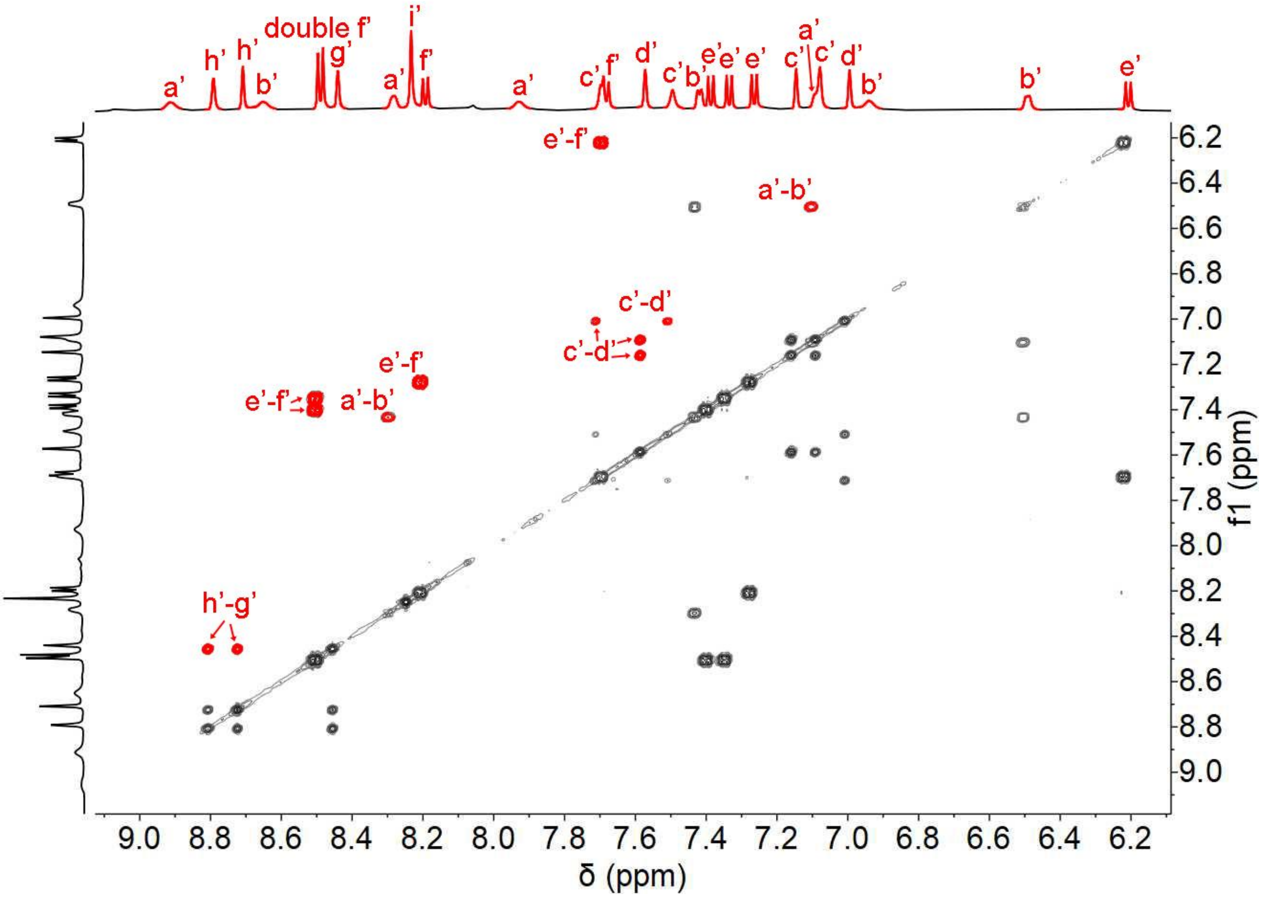

Supplementary Figure 58. ^{1}H-^{1}H COSY NMR spectrum (600 MHz, acetone-d_6, 298 K) of **9** at 15 mM.

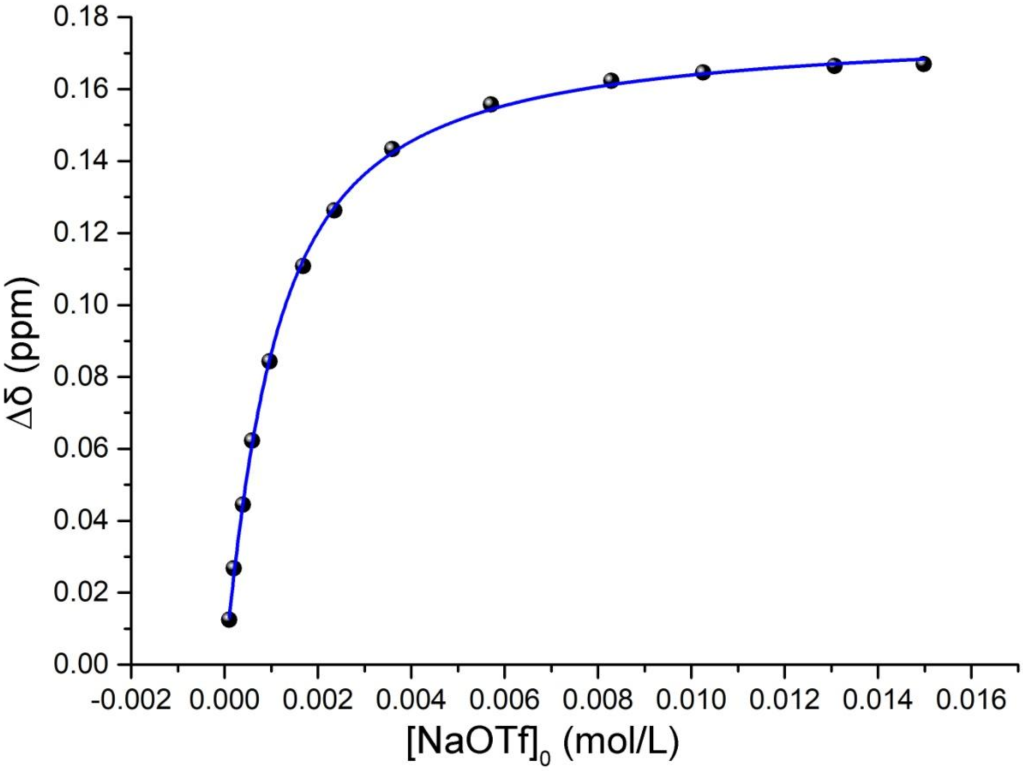

Supplementary Figure 59. The non-linear curve-fitting (NMR titrations) for the complex of NaOTf ⊂ **6**, K_a = (1270 ± 30) M^{-1}.

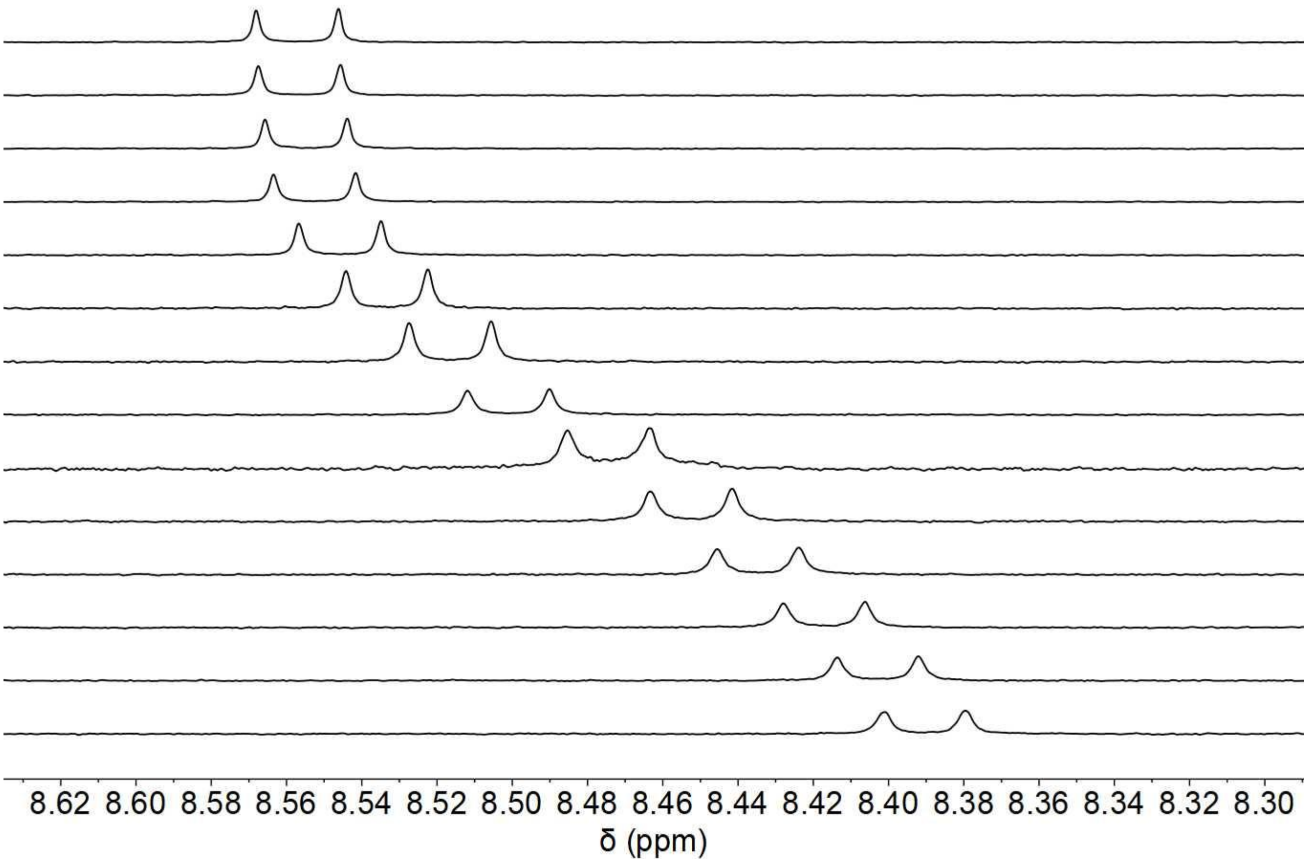

Supplementary Figure 60. Partial ^{1}H NMR spectra (400 MHz, in acetone-d_6 at 298 K) of **6** (H_f) at a concentration of 0.50 mM upon addition of NaOTf. From bottom to top, the concentration of NaOTf was 0.00, 0.10, 0.20, 0.39, 0.59, 0.96, 1.68, 2.36, 3.60, 5.71, 8.29, 10.3, 13.1 and 15.0 mM.

For the host−guest pairs, chemical exchange is fast on the NMR time scale. To determine the association constant, NMR titrations were done with solutions which had a constant concentration of **6** and varying concentrations of NaOTf. Using the nonlinear curve-fitting method, the association constant was obtained for each host-guest combination from the following equation:

$A = (A_\infty/[G]_0)\ (0.5[H]_0 + 0.5([G]_0+1/K_a)-(0.5\ ([H]_0^2+(2[H]_0(1/K_a - [G]_0)) + (1/K_a + [G]_0)^2)\ ^{0.5}))$ (Supplementary equation 1)

Where A is the chemical shift change of **6**, A_∞ is the chemical shift change when the NaOTf is completely complexed, $[H]_0$ is the fixed initial concentration of the **6**, and $[G]_0$ is the initial concentration of NaOTf. The association constants (K_a) was calculated by using the nonlinear curve-fitting method (Supplementary Figures 59 and 60).

Supplementary References

(1) Newkome, G. R., Garbis, S. J., Majestic, V. K., Fronczek, F. R. & Chiari, G. Chemistry of heterocyclic compounds. 61. Synthesis and conformational studies of macrocycles possessing 1,8- or 1,5-naphthyridino subunits connected by carbon-oxygen bridges. *J. Org. Chem.* **46**, 833-839 (1981)
(2) Wang, M., Zheng, Y.-R., Ghosh, K. & Stang, P. J. Metallosupramolecular tetragonal prisms via multicomponent coordination-driven template-free self-assembly. *J. Am. Chem. Soc.* **130**, 6282−6283 (2010).

(3) Park, J., Wang, Z. U., Sun, L.-B., Chen, Y.-P. & Zhou, H.-C. Introduction of Functionalized Mesopores to Metal–Organic Frameworks via Metal–Ligand–Fragment Coassembly. *J. Am. Chem. Soc.* **134**, 20110-20116 (2012).
(4) Krzyzanowski, A., Saleeb, M. & Elofsson, M. Synthesis of Indole-, Benzo[*b*]thiophene-, and Benzo[*b*]selenophene-Based Analogues of the Resveratrol Dimers Viniferifuran and (±)-Dehydroampelopsin B. *Org. Lett.* **20**, 6650-6654 (2018).
(5) Sheldrick, G. M. Crystal structure refinement with *SHELXL*. *Acta Crystallogr. C*, **71**, 3–8 (2015).
(6) Dolomanov, O. V., Bourhis, L. J., Gildea, R. J., Howard, J. A. K. & Puschmann, H. *OLEX2*: a complete structure solution, refinement and analysis program. *J. Appl. Cryst.* **42**, 339–341 (2009).
